# Computational approaches for drug–drug interaction prediction: a systematic review of data sources, modeling strategies, and evaluation frameworks

**DOI:** 10.3389/fphar.2026.1816394

**Published:** 2026-04-29

**Authors:** Qadeer Hashir, Muhammad Asfand E Yar, Asad Ullah, Shahid Kamal, Fasee Ullah, Zul Hilmi Abdullah

**Affiliations:** 1 Center of Excellence in Artificial Intelligence (CoE-AI), Department of Computer Science, Bahria University, Islamabad, Pakistan; 2 Center for Advanced Analytics, CoE for Artificial Intelligence, Faculty of Computing and Informatics, Multimedia University, Cyberjaya, Malaysia; 3 Department of Computing, Universiti Teknologi PETRONAS, Seri Iskandar, Malaysia; 4 Faculty of Computing and Informatics, Multimedia University, Cyberjaya, Malaysia

**Keywords:** computational prediction, drug–drug interactions, explainable AI, GNNS, multimodal learning

## Abstract

**Introduction:**

Drug-drug interactions (DDIs) are a major cause of preventable harm in polypharmacy and remain difficult to anticipate as formularies, indication profiles, and interaction labels evolve. Over the last few years, the DDI modeling landscape has shifted rapidly toward graph-native, multimodal, and contrastive or self-supervised learning, alongside renewed interest in extraction, decision support, and pharmacovigilance pipelines.

**Objective:**

This systematic literature review (SLR) synthesizes computational work on DDI prediction, event-type classification, text extraction, and safety signal detection published between 2022 and 2025. We aim to (i) organize recent methods into a feature–method taxonomy, (ii) compare their evaluation setups and reported performance, and (iii) assess progress on generalization, explainability, and clinical translation.

**Methods:**

Using a prespecified review protocol and PRISMA 2020 reporting guidance, we searched major bibliographic databases and screened peer-reviewed studies that proposed or evaluated computational methods for DDIs or closely related interaction tasks. Eligible work spans molecular graph and descriptor models, multimodal pharmacological representations, heterogeneous and knowledge graphs, text-based extraction and retrieval, and real-world evidence from EHRs, FAERS, and similar sources. We grouped methods into similarity and matrix-factorization baselines, conventional machine learning, deep neural architectures (CNNs, RNNs, and Transformers), graph neural networks and knowledge-graph representation learning, multimodal fusion, contrastive/self-supervised objectives, and emerging LLM-based frameworks. For each study, we extracted feature modalities, tasks, datasets and splits, metrics, explainability tools, and any form of clinical or user-centred evaluation.

**Results:**

Recent work consistently reports improved AUROC/AUPR on DrugBank-derived, TWOSIDES-like, and DDIExtraction benchmarks, driven by substructure-aware GNNs, KG-augmented architectures, multimodal fusion, and inductive or out-of-distribution training regimes. However, most models still rely on a small set of public datasets, heterogeneous and sometimes optimistic split protocols, and limited external or prospective validation. Event-level and long-tailed risk modeling, prompt- or prototype-based learning, and LLM-assisted extraction strengthen coverage of rare but clinically important interaction types, yet uncertainty quantification, label quality assessment, and end-to-end integration into prescribing workflows remain underexplored.

**Discussion:**

Between 2022 and 2025, DDI modeling has moved decisively toward graph-centric, multimodal, and contrastive/self-supervised paradigms that clearly advance benchmark performance but only partially close the gap to reliable, mechanism-aware clinical decision support. We distill design guidelines and a research agenda around transparent dataset construction, realistic and standardized evaluation protocols, mechanism- and direction-aware modeling, robustness to novel drugs and regimens, and prospective, clinician-in-the-loop validation.

## 1 Introduction

### 1.1 Motivation and clinical impact

Patients with complex conditions are often exposed to long drug lists, managed by multiple prescribers, and treated with regimens that change over time. In these settings, DDIs can attenuate efficacy, trigger serious adverse events, or destabilize chronic disease control. Conventional strategies for managing this risk rely on a mix of *in vitro* studies, dedicated clinical DDI trials, spontaneous reporting systems, curated interaction compendia, and rule-based clinical decision support in electronic health records. These tools are valuable, but they struggle to keep pace with the growth of available drugs, indications, and off-label uses, and they are biased toward well-studied, high-signal interactions.

Computational methods offer a way to systematically mine and integrate diverse evidence sources: molecular structures and properties, drug–target and pathway networks, pharmacokinetic and pharmacodynamic data, biomedical literature, spontaneous reports, and longitudinal EHRs. Early work focused on similarity measures and matrix factorization over DrugBank-style graphs or co-prescription matrices. More recent models treat drugs and related entities as nodes in large heterogeneous networks, represent molecules as graphs or multimodal objects, and use neural architectures to capture complex interaction mechanisms, including polypharmacy side effects and dose- or context-dependent risks.

Between 2022 and 2025, this ecosystem changed again. Substructure-aware GNNs, heterogeneous and knowledge-graph models, multimodal fusion of chemistry, targets, pathways, and clinical context, and contrastive or self-supervised pretraining have become dominant. At the same time, new work targets event-level interaction types, long-tailed and rare but high-impact events, inductive generalization to unseen drugs, and integration of DDI awareness into recommender systems for prescriptions and medication reviews. Text-based pipelines increasingly rely on Transformer models and, more recently, LLM-augmented or prompt-driven extraction frameworks, while pharmacovigilance and EHR-based studies explore graph-based representations of patients and their medication histories.

**FIGURE 1:**
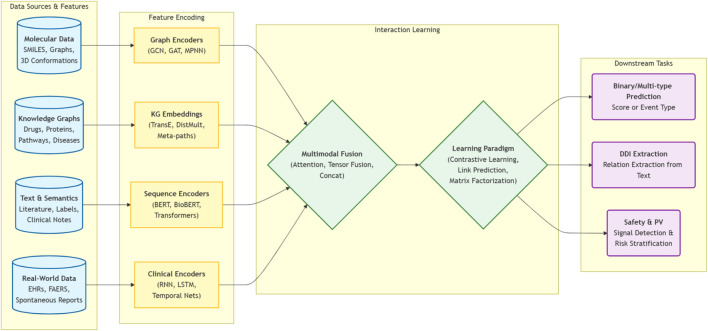
A generic computational framework for drug-drug interaction (DDI) prediction.

Yet clinical practice is still constrained by several gaps. Many models are evaluated only on a narrow set of public benchmarks, often with heterogeneous or optimistic split strategies. Mechanism and direction of interaction (for example, victim versus perpetrator, pharmacokinetic versus pharmacodynamic) are not always modeled explicitly. Real-world issues such as label noise, confounding, temporal drift in interaction knowledge, and workflow integration are only ly addressed. Explainability is often reduced to attention heatmaps or substructure visualizations, with limited connection to regulatory requirements or clinician mental models. This creates a need for an up-to-date, method-oriented SLR that looks beyond raw metrics to how recent methods are built, evaluated, and positioned for translation. Figure 1 shows a generic computational framework for drug-drug interaction (DDI) prediction.

### 1.2 Research questions

This review focuses on computational approaches to DDI prediction, event-type classification, text-based interaction extraction, and related safety-signal detection published between 2022 and 2025. We use a feature–method taxonomy to organize this work and address the following research questions:

- RQ1: What feature modalities are used to model and detect DDIs (molecular, pharmacological, network/knowledge-graph, textual, and real-world evidence), and how are they combined within multimodal pipelines?
- RQ2: Which computational method families are most prominent in recent work (e.g., similarity and matrix-factorization baselines, classical machine learning, deep neural models, GNN/knowledge-graph approaches, contrastive/self-supervised learning, and LLM- or prompt-driven methods), and how are they distributed across tasks and data settings?
- RQ3: How do dataset choices, label definitions, evaluation protocols (including random, temporal, scaffold, and cold-start/inductive settings), and reported metrics shape performance claims and generalization to unseen drugs or regimens under distribution shift?
- RQ4: What practices are reported to support reproducibility, interpretability, and clinical translation, including code/data availability, experimental transparency, explainability methods, uncertainty estimation, and user or expert evaluation?
- RQ5: Where do current approaches fall short in terms of label quality, mechanism- and direction-aware modelling, temporal and population generalization, and integration into real-world prescribing and pharmacovigilance workflows, and what concrete directions emerge for future research?

## 2 Methodology (PRISMA-2020)

### 2.1 Protocol and registration

**FIGURE 2:**
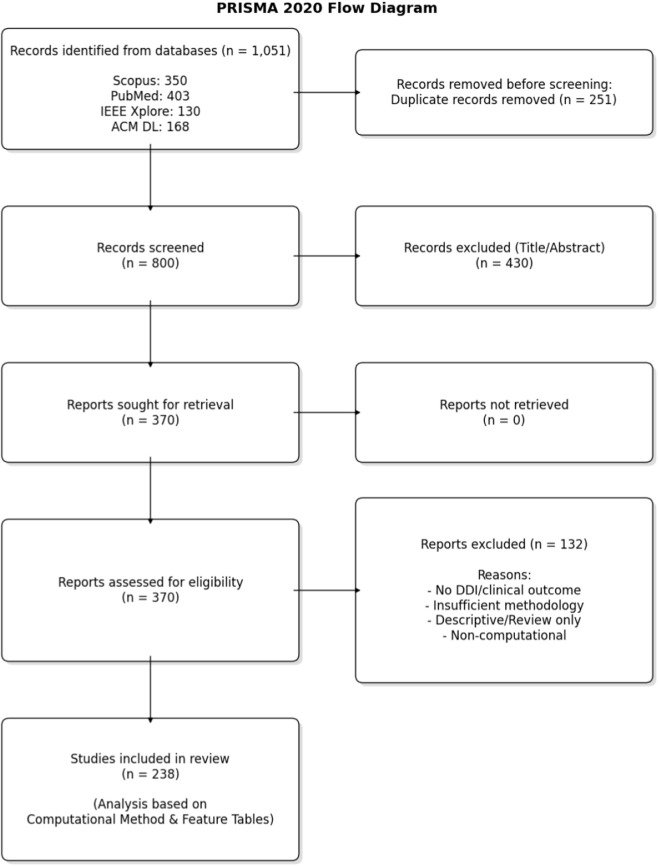
PRISMA 2020 flow diagram of study identification, screening, eligibility assessment, and inclusion for this review (Matthew et al., 2021).

This review used a prespecified protocol covering the research questions, eligibility criteria, screening plan, and extraction schema, which was finalized prior to screening. The review followed PRISMA 2020 (Matthew et al., 2021) reporting guidance for study identification, screening, eligibility assessment, and structured reporting. Because this work synthesizes heterogeneous computational and methodological DDI studies rather than intervention-effect studies, PRISMA 2020 was used as a reporting framework rather than as a claim of full item-by-item compliance in every respect. The review was not prospectively registered. Figure 2 presents the PRISMA 2020 flow diagram for study identification and selection.

### 2.2 Eligibility criteria

We targeted *computational* research related to drug-drug interaction (DDIs) and closely allied interaction tasks. Studies were included if they met all of the following criteria:

- Publication type and language: Peer-reviewed journal articles or full conference papers (including methodological reviews) written in English.
- Time window: Published or available online between 2022 and 2025 (inclusive).
- Computational focus: Proposed, evaluated, or synthesised a computational approach relevant to DDI modelling, including (but not limited to) machine learning, deep learning, graph learning (e.g., GNNs), knowledge-graph methods, contrastive/self-supervised learning, transformer/LLM-based methods, text mining/information extraction, QSAR/cheminformatics, pharmacovigilance signal detection, and multimodal evidence fusion.
- Task relevance: Addressed at least one DDI-relevant outcome such as (i) binary or multi-type DDI prediction, (ii) interaction event/type prediction (including severity/direction where defined), (iii) DDI extraction/classification from text corpora, (iv) enzyme/transporter-mediated interaction risk when explicitly framed as DDI-relevant, or (v) safety signal detection from real-world sources where outcomes are explicitly tied to drug combinations.
- Reporting sufficiency: Provided enough information to extract the study-level fields used in this review (computational approach, dataset/data source(s), feature/evidence types, and code availability).

We excluded:

- purely clinical case reports or small uncontrolled series without an explicit computational method,
- editorials, letters, short commentaries, abstracts-only records, and theses,
- papers where “interaction” was unrelated to drug combinations (e.g., protein–protein interactions without a DDI framing),
- generic ML papers that mention DDIs only as a minor example without DDI-specific analysis/evaluation.

### 2.3 Information sources and search strategy

We searched four bibliographic databases selected to cover biomedical/pharmacological and computer-science venues: Scopus, PubMed, IEEE Xplore, and the ACM Digital Library. Searches were restricted to 2022–2025 and applied to titles/abstracts/keywords where supported. The search strategy combined (i) DDI terms (e.g., drug–drug interaction, drug interaction): and (ii) computational terms (e.g., machine learning, deep learning, graph neural network, knowledge graph, natural language processing, text mining).

#### 2.3.1 Supplementary searches

Backward citation screening was conducted on relevant recent reviews/surveys/framework papers to reduce the risk of missing influential or newly indexed work. Any additional records were subjected to the same eligibility and screening criteria as database hits.

### 2.4 Study selection

All retrieved records were exported to a single library and deduplicated using persistent identifiers (e.g., DOI/PMID where available) and near-duplicate matching on title and author lists. Screening proceeded in two stages:

- Title/abstract screening: records were excluded if clearly non-computational, not DDI-relevant, or not a full research paper/review.
- Full-text screening: remaining articles were assessed for DDI-task relevance and reporting sufficiency for extraction.

The final included corpus comprised 238 distinct included studies, enumerated in Table 5).

#### 2.4.1 Clarifying “included studies” versus “total references”

In PRISMA terminology, the included corpus counts *studies* (unique eligible works included in extraction). The manuscript bibliography may include additional references that are not part of the included corpus (e.g., PRISMA guidance, dataset/resource papers, foundational ML methods, and background/context citations). Therefore, the total number of references in the bibliography is expected to exceed the number of included studies.

### 2.5 Data extraction

We used a structured extraction form aligned with the study-level comparison table. For each included study, we extracted the following fields:

- Reference key and publication year,
- Computational approach (as described by the authors),
- Dataset/data source(s) used for training and/or evaluation,
- Feature/evidence types (e.g., chemical structure, targets/pathways, KG/network signals, text/EHR/FAERS-derived evidence),
- Code availability (repository/link reported vs. not reported), summarized in Supplementary Table S1.

### 2.6 Deriving method and feature taxonomies

To enable consistent aggregation across heterogeneous study descriptions without altering any study-level facts:

- Each study was assigned a single primary method family (Table 2) by mapping the extracted “Computational Approach” text to the best-matching category using deterministic keyword rules;
- Each study was assigned one or more feature/evidence categories (Table 1) using multi-label keyword rules applied to the extracted “Feature Types” text (therefore category totals may exceed N ).

**TABLE 1:** Feature summary (N = 238). Counts are multi-label: a single study may be assigned to more than one feature category based on deterministic keyword matching of the extracted “Feature Types”. Percentages are reported as the proportion of included studies (N = 238) assigned to each category, not as percentages of total category assignments; therefore, the counts can sum to more than 238 and the percentages can sum to more than 100%.

Feature category	#Studies	% (of N)
Knowledge graph/network relations	111	46.6%
Other reported feature types	56	23.5%
Chemical structure/fingerprints	52	21.8%
Text/literature/EHR	40	16.8%
Proteins/targets	34	14.3%
Pathways/functional annotations	10	4.2%
Diseases/indications	7	2.9%
Omics/gene expression	7	2.9%
Side effects/ADRs	6	2.5%

### 2.7 Quality/risk of bias (RoB-ML)

Because conventional clinical risk-of-bias tools do not map cleanly to heterogeneous computational DDI studies, we used a tailored RoB-ML rubric with three domains: bias risk, data-source quality, and reproducibility. Bias risk captured concerns such as possible leakage-prone evaluation designs, limited visibility of negative-sampling or split strategies, and other reporting gaps that could make performance appear more optimistic than it would be under stricter validation. Data-source quality captured the transparency and credibility of the underlying datasets or source resources, including whether benchmark names, source provenance, and source granularity were reported clearly enough to support interpretation. Reproducibility captured whether the study reported an accessible code repository or executable resource and whether the study summary provided enough implementation transparency for readers to understand how the method was operationalised.

Each domain was rated at three levels: low concern, moderate concern, or high concern. For transparent synthesis, these levels were mapped to ordinal values of 2, 1, and 0, respectively, and summed to obtain a composite RoB-ML score ranging from 0 to 6 for each study. These scores were used only for structured comparison and narrative synthesis; they were not used as exclusion criteria and were not combined in a quantitative meta-analysis. A domain-level summary of the RoB-ML assessment is now provided in Table 3, and the study-level ratings are reported in Supplementary Table S2.

### 2.8 Synthesis methods

Given heterogeneity in datasets, interaction definitions, and evaluation protocols, we did not conduct a quantitative meta-analysis. Instead, we performed a structured narrative synthesis:

- Studies were summarized by method family (Table 2) and feature/evidence modality (Table 1),
- Study characteristics were summarized in Table 5, with the complete Code-level extraction provided in Supplementary Table S1, and RoB-ML quality assessments summarized in Table 3, and Supplementary Table S2.
- Comparisons were interpreted within the task definitions and evaluation assumptions reported by the original authors.

**TABLE 2:** Summary by method type (N = 238). Groups are assigned by deterministic keyword rules applied to the “Computational Approach”.

Method type	#Studies	%
Multimodal fusion	45	18.9%
Text mining/IE/corpus	29	12.2%
Deep learning (non-graph)	25	10.5%
Graph neural network	21	8.8%
Review/Survey	20	8.4%
Contrastive/metric/meta-learning	17	7.1%
Knowledge graph/embedding	14	5.9%
Transformer/LLM	12	5.0%
Representation learning/embedding models	11	4.6%
Heterogeneous network/meta-path	5	2.1%
Recommender systems	5	2.1%
Clinical study/trial/cohort	5	2.1%
Classical machine learning	5	2.1%
Pharmacovigilance signal detection	4	1.7%
Statistical/regression	4	1.7%
Bioinformatics/omics analysis	3	1.3%
Matrix factorization/tensor factorization	3	1.3%
Cheminformatics/QSAR	2	0.8%
Network-based (non-GNN)	2	0.8%
Resource/benchmark/comparison	2	0.8%
Reinforcement learning	2	0.8%
Computational modeling (general)	1	0.4%
Software tool/library	1	0.4%

**TABLE 3:** RoB-ML summary of the assessed corpus. The table highlights the dominant domain-level quality patterns and the most recurrent concerns identified during narrative appraisal.

RoB-ML domain	Dominant pattern	Typical concern in the assessed corpus	Interpretation for the review
Bias risk	Predominantly moderate concern	Split strategy, leakage control, and negative-sampling details are often not fully recoverable from condensed study summaries	Reported performance should be interpreted cautiously unless validation design is explicitly described elsewhere in the full paper
Data-source quality	Mixed low-to-moderate concern	Many studies rely on named public benchmarks, but some entries use generic dataset labels or incomplete source descriptions	Data transparency varies substantially across the literature, which affects comparability and auditability
Reproducibility	Predominantly moderate concern with recurring high-concern cases	Code or executable resources are not consistently reported, and condensed study tables do not always provide enough implementation detail for full reproduction	Reproduction and external checking remain uneven across recent computational DDI studies
Overall synthesis	Predominantly moderate overall concern	The strongest entries combine named datasets with code/resource links, whereas weaker entries remain difficult to audit because both data transparency and reproducibility are limited	The literature is methodologically active and promising, but still uneven in transparency and translational readiness

A completed PRISMA 2020 checklist has been added to the Supplementary Material. Methodological quality was appraised using the tailored RoB-ML rubric described below. A formal certainty-of-evidence framework was not applied because the included literature was highly heterogeneous in study design, task formulation, datasets, labels, and reported outcomes, and the review did not perform a pooled meta-analytic synthesis.

## 3 Taxonomy: features and methods

**FIGURE 3:**
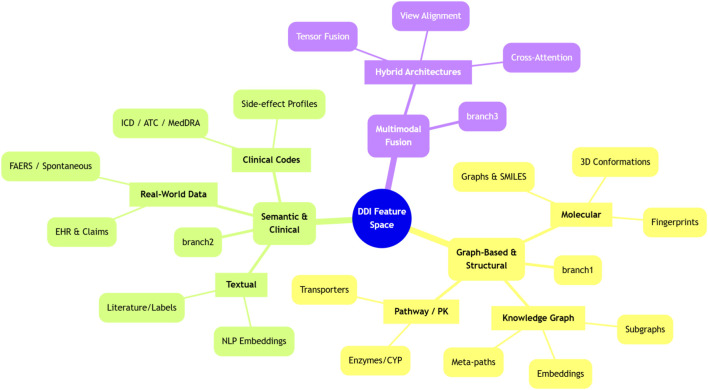
Taxonomy of feature representations utilized in DDI prediction studies.

This section organises the included studies into a concise taxonomy of feature families and methodological families for computational drug–drug interaction modelling. Feature families describe how drugs, interactions, and clinical context are represented, whereas method families summarise the main learning strategies applied to those features. Figure 3 summarizes the feature-representation taxonomy used in the included DDI prediction studies.

### 3.1 Feature taxonomy and representative studies

The feature taxonomy groups existing work according to the primary information sources used to represent drugs and interactions, including knowledge graphs, molecular structure, multimodal combinations, mechanistic and pathway information, real-world data, clinical code systems, and textual or semantic signals. Instead of describing every study in detail, we highlight common design choices and representative lines of work within each feature family.

#### 3.1.1 Knowledge-graph features

Knowledge-graph based approaches construct heterogeneous biomedical graphs where drugs, proteins, diseases, and adverse events are connected by typed relations and then learn node or subgraph representations using embedding, message-passing, or attention mechanisms (Tassallah, 2025; Wang Huan, 2024; Chen and Qu, 2025; Cheng Zihui et al., 2025; Feng et al., 2022; Gu et al., 2023; He et al., 2024; Jiang and Zhang, 2024; Ye et al., 2022; Kontsioti et al., 2022; Li, 2023; Xiang, 2024; Lu et al., 2022; Mydhili, 2025; Simone, 2024; Sun et al., 2022; Tanvir et al., 2024; Wang Hong et al., 2025; Yang, 2024; Yu et al., 2023a; Zhang, 2023; Zhang and Zhao, 2024; Zhao et al., 2025; Barkova and Mottin, 2025). Many studies exploit multi-hop paths, meta-paths, and higher-order neighbourhoods to capture mechanistic routes linking drug pairs to shared targets, pathways, or phenotypes, often using these structures as explanations for predicted interactions (Mir et al., 2025; Miwa and Sasaki, 2023; Cheng et al., 2024; Ding et al., 2025; Gao Peng, 2024; Guan et al., 2022; Hao et al., 2023; Jabarin et al., 2024; Jiang et al., 2024a; Su et al., 2023; Weisu, 2023; Yu, 2024; Lin Xiaoli, 2024; Tengfei, 2024; Peng and Ji, 2024; Su et al., 2025; Taneja et al., 2023; Tanvir, 2024; Wen, 2023; Zhang Ran et al., 2023; Ran, 2024; Zhao, 2025). Recent work integrates textual, molecular, and real-world evidence into knowledge-graph backbones and explores self-supervised, contrastive, or curriculum-style training strategies to improve generalisation and interpretability (Gao Peng, 2024; Amiri Souri et al., 2023; Chen, 2023; Kang and Yin, 2024; Guo et al., 2023; Hartung et al., 2022; Jiang, 2023; Jin, 2022; Zhong et al., 2024; Li Zhe et al., 2023; Xinyue, 2024; Mirthika et al., 2024; Pfeifer et al., 2022; Sun et al., 2022; Tanvir, 2023; Wang Yaqing et al., 2024; Yu et al., 2023b; Zhang Yuchen et al., 2022; Zhang Jing et al., 2023; Changpeng, 2025).

#### 3.1.2 Molecular graphs/fragments/fingerprints

Structure-based approaches encode each drug as a molecular graph, a set of chemical fragments, or a high-dimensional fingerprint, and then derive feature vectors through expert-crafted descriptors, graph neural encoders, or deep fingerprinting networks (Chen and Qu, 2025; Xiang, 2024; Zhao et al., 2025; Mir et al., 2025; Hao et al., 2023; Su et al., 2023; Guo et al., 2023; Zhong et al., 2024; Gao Jianliang, 2024; Das, 2025; Dong, 2024; Feng Yue-Hua et al., 2023; Jain et al., 2023; Jiang et al., 2024b; Lakizadeh and Babaei, 2022; Li Zimeng et al., 2023; Luo et al., 2025; Ali Muhammed, 2025; Pham et al., 2024; Qiao et al., 2025; Benedek, 2022; Sun Chang et al., 2023; Tang et al., 2024; Srinivasan and Pathak, 2024; Wang Guishen, 2024; Wang and Taylor, 2025; Xia et al., 2025; Yang et al., 2022; Yang et al., 2025; Yuan et al., 2025; Zhang, 2024). These representations are used in classifiers and ranking models to infer whether a pair of drugs is likely to interact, emphasising how substructure patterns, pharmacophore motifs, and physicochemical properties correlate with interaction risk (Cheng Zihui et al., 2025; Jiang and Zhang, 2024; Yu et al., 2023a; Ding et al., 2025; Wen, 2023; Zhang Ran et al., 2023; Ran, 2024; Li Zhe et al., 2023; Tanvir, 2023; Changpeng, 2025; Ali Sabir, 2025; Chen, 2024; Dou, 2024; Gong et al., 2025; Guo et al., 2025; He Haohuai et al., 2022; Huang An et al., 2023; Jiang et al., 2024a; Rogia, 2024; Lee et al., 2023; Li Tonglei et al., 2025; Yue et al., 2025; Li, 2025; Petschner, 2025; Priya et al., 2022; Ran et al., 2022; Ru et al., 2023; Shen Xiangzhen et al., 2023; Sun and Zheng, 2025; Wang Zhen et al., 2024; Hong, 2025; Jiang et al., 2022; Yang, 2023; Ye and Lin, 2023; Zhang and Jin, 2025; Chuang et al., 2024; Zhou et al., 2024). Several studies further combine multiple levels of structural information or augment molecular descriptors with auxiliary pharmacological features to improve predictive performance (Feng et al., 2022; Gu et al., 2023; Simone, 2024; Miwa and Sasaki, 2023; Jiang et al., 2024a; Yu, 2024; Kang and Yin, 2024; Zhang Jing et al., 2023; Ran et al., 2022; Chen et al., 2024; Cheng et al., 2025a; He et al., 2025; Iqbal, 2024; Jin, 2024; Kumari, 2024; Yue and Gu, 2025; Liu et al., 2023; Lv, 2023; Ning et al., 2023; Pham et al., 2024; Qian et al., 2022; Shtar et al., 2023; Tang, 2024; Tran, 2025; Wang Yingying et al., 2024; Xia, 2025; Guihua, 2022; Yang Zihao et al., 2023; Yin, 2023; Qi et al., 2024; Zhang Shuai, 2025; Zhao Ke et al., 2024; Liangwei, 2024; Shen and Lu, 2022).

#### 3.1.3 Multimodal fusion

Multimodal fusion approaches combine complementary views of each drug, such as chemical structure, protein targets, indications, pathways, knowledge-graph neighbourhoods, text, and observational clinical signals (Cheng Zihui et al., 2025; Gu et al., 2023; Miwa and Sasaki, 2023; Ding et al., 2025; Jabarin et al., 2024; Lin Xiaoli, 2024; Zhao, 2025; Xinyue, 2024; Zhang Jing et al., 2023; Lakizadeh and Babaei, 2022; Ali Sabir, 2025; Ru et al., 2023; Sun and Zheng, 2025; Yang, 2023; Chuang et al., 2024; Jin, 2024; Wang Yingying et al., 2024; Bayraktar et al., 2025; Dong et al., 2024; Gan et al., 2023; Gupta, 2023; Saha and Karmakar, 2023; Nasiri and Hooshmand, 2025; Qi et al., 2025; Zhang and Zhang, 2025). Typical architectures allocate a dedicated encoder to each modality and then fuse the resulting embeddings via concatenation, attention, gating, or tensor-based operators before making DDI predictions (Chen and Qu, 2025; Xiang, 2024; Wang Hong et al., 2025; Zhang and Zhao, 2024; Zhao et al., 2025; Gao Peng, 2024; Kang and Yin, 2024; Guo et al., 2023; Jiang, 2023; Li Zhe et al., 2023; Qiao et al., 2025; Wang Guishen, 2024; Yuan et al., 2025; He Haohuai et al., 2022; Cheng et al., 2025a; Pham et al., 2024; Tran, 2025; Xia, 2025; Yang Zihao et al., 2023; Liangwei, 2024; Fadwa, 2023; Astras and Vogiatzis, 2024; Huang Licai et al., 2022; Ha, 2022; Liu, 2025; Machado, 2025; Saad et al., 2025a). Across datasets, these multimodal designs consistently report gains over single-modality baselines, suggesting that heterogeneous evidence is important for robust and clinically meaningful interaction modelling (Jiang and Zhang, 2024; Mydhili, 2025; Simone, 2024; Yu, 2024; Wen, 2023; Zhang Ran et al., 2023; Ran, 2024; Changpeng, 2025; Dong, 2024; Feng Yue-Hua et al., 2023; Pham et al., 2024; Benedek, 2022; Huang An et al., 2023; Hong, 2025; Jiang et al., 2022; He et al., 2025; Kumari, 2024; Liu et al., 2023; Yin, 2023; Ilidio and Cerri, 2023; Kivanc, 2025; Cheng Junkai et al., 2025; Demirsoy and Karaibrahimoglu, 2023; Gao et al., 2025; Song and Yang, 2025; Eugene, 2024; Li Rongpei et al., 2025; Junyu, 2023; Wang, 2023).

#### 3.1.4 Pathway/PK/mechanistic

Mechanistic and pathway-oriented studies incorporate pharmacokinetic and pharmacodynamic knowledge, as well as biological pathway information, to ground interaction predictions in interpretable processes such as shared metabolism, transporter competition, or signalling crosstalk (Kontsioti et al., 2022; Tanvir et al., 2024; Huang Licai et al., 2022; Ha, 2022; Emami and Ferdousi, 2024; Gill et al., 2022; Olubamiwa et al., 2025; Jun et al., 2022; Zhang Teng et al., 2022; Tie, 2023). Models in this family encode drug–enzyme and drug–transporter relationships, metabolic routes, dose or exposure profiles, and sometimes time-dependent concentration curves and then relate these signals to interaction outcomes (Cheng Zihui et al., 2025; Simone, 2024; Gao Peng, 2024; Tanvir, 2024; Lakizadeh and Babaei, 2022; Sun Chang et al., 2023; Huang An et al., 2023; Xia, 2025; Eugene, 2024; Gill et al., 2023; Zheng et al., 2024; Zhu Hao et al., 2023). By aligning predictions with ADME mechanisms and pathway perturbations, these approaches support mechanistic explanations that are closer to how clinicians reason about DDIs (Taneja et al., 2023; He et al., 2025; Iqbal, 2024; Liangwei, 2024; Demirsoy and Karaibrahimoglu, 2023; Gao et al., 2024; Kim and Nam, 2022; Junlong, 2024; Shen Ziyi et al., 2023; Wang and Yu, 2023; Yang et al., 2024).

#### 3.1.5 Real-world/EHR/FAERS

Real-world data approaches mine longitudinal electronic health records, claims databases, registries, and spontaneous reporting systems such as FAERS to detect and validate potential DDIs (Simone, 2024; Weisu, 2023; Chen, 2023; Tanvir, 2023; Eugene, 2024; Junlong, 2024; Qamar et al., 2025; Ayman, 2025; Xu et al., 2024). They characterise co-prescription patterns, temporal treatment trajectories, laboratory measurements, and reported adverse events for drug pairs and then use statistical metrics or machine learning models to quantify interaction signals (Wang Huan, 2024; Kontsioti et al., 2022; Li, 2023; Sun et al., 2022; Chuang et al., 2024; Yongjian, 2022; Shoaib, 2024; Qin et al., 2024; Agarwal and Pandey, 2023). Many recent studies also link real-world signals with knowledge-driven or mechanistic features to reduce confounding and support more reliable risk assessment (Sun et al., 2022; Mirthika et al., 2024; He et al., 2025; Astras and Vogiatzis, 2024; Liu, 2025; Kunakorntham et al., 2022; Ren et al., 2024; Sven, 2022a; Zhu Wenjun et al., 2023).

#### 3.1.6 Side-effect/clinical codes

Side-effect and clinical-code based approaches represent drugs using their associated adverse-event profiles and structured clinical vocabularies such as ICD, ATC, or MedDRA (Cheng Zihui et al., 2025; Gu et al., 2023; Lakizadeh and Babaei, 2022; Srinivasan and Pathak, 2024; Guihua, 2022; Fadwa, 2023; Eugene, 2024; Junlong, 2024). Methods typically embed drugs and clinical events into a shared latent space using co-occurrence patterns, hierarchical relationships, or multi-label prediction tasks and then exploit distances or similarity scores to identify candidate DDIs (Tanvir et al., 2024; Weisu, 2023; He et al., 2025; Kumari, 2024; Qamar et al., 2025; Sven, 2022a; Abbas, 2023). This family demonstrates that rich phenotypic signatures and code-system structure can reveal interaction patterns even when detailed mechanistic data are limited (Tanvir, 2024; Wang and Taylor, 2025; Astras and Vogiatzis, 2024; Junyu, 2023; Yongjian, 2022; Kunakorntham et al., 2022).

#### 3.1.7 Textual/semantic

Textual and semantic approaches exploit unstructured or semi-structured biomedical text from drug labels, guidelines, clinical narratives, and scientific literature to represent drugs and interactions (Mydhili, 2025; Gao Peng, 2024; Jin, 2022; Xia et al., 2025; Dou, 2024; Chuang et al., 2024; He et al., 2025; Zhang and Zhang, 2025; Fadwa, 2023; Astras and Vogiatzis, 2024; Junyu, 2023; Shoaib, 2024; Agarwal and Pandey, 2023; Abbas, 2023; Jia, 2024; Keshavarz and Lakizadeh, 2025; Shi et al., 2022; Yang Jie et al., 2023). Pipelines range from traditional bag-of-words and dependency-based features to contextual language models that encode drug descriptions, indication narratives, and interaction sections into high-dimensional embeddings (Chen and Qu, 2025; Gu et al., 2023; Weisu, 2023; Feng Yue-Hua et al., 2023; Sun and Zheng, 2025; Iqbal, 2024; Pham et al., 2024; Shen and Lu, 2022; Kivanc, 2025; Junlong, 2024; Wang and Yu, 2023; Alrowais et al., 2023; Qiu et al., 2022; Molina et al., 2023; Yadav, 2022; Zhu et al., 2025). The resulting representations can be used directly for DDI classification or combined with structured knowledge, showing that language captures complementary pharmacological information relevant to interaction detection (Cheng Zihui et al., 2025; Jiang and Zhang, 2024; Lu et al., 2022; Miwa and Sasaki, 2023; Gao Peng, 2024; Wen, 2023; Dou, 2024; Yang, 2023; Tran, 2025; Bayraktar et al., 2025; Qi et al., 2025; Machado, 2025; Song and Yang, 2025; Ayman, 2025; KafiKang and Hendawi, 2023; Zaikis and Vlahavas, 2025; Zhang Ting et al., 2025).

### 3.2 Methods taxonomy and representative studies

**FIGURE 4:**
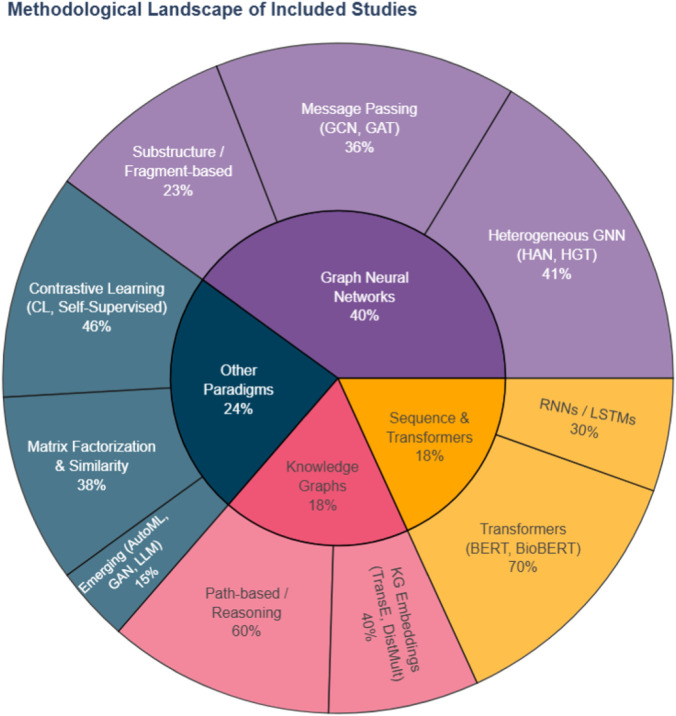
Distribution of computational methodologies across the reviewed studies (2022–2025).

The methods taxonomy groups the included studies according to the dominant learning paradigm or model class used for DDI prediction, rather than the underlying features alone. We distinguish adversarial and generative approaches, automated model search, contrastive and representation learning methods, graph neural networks, similarity-based models, text-mining pipelines, transformer architectures, and other relevant families, highlighting how they exploit the available data sources. Figure 4 summarizes the distribution of computational methodologies across the included studies (2022–2025).

#### 3.2.1 Adversarial (GAN)

Adversarial approaches cast parts of the DDI modelling task as a game between generators and discriminators, for example, synthesising realistic interaction patterns, augmenting minority classes, or denoising sparse signals (Yu et al., 2023a; Ali Sabir, 2025). Such models aim to improve generalisation by exposing the predictor to harder or more balanced examples, and they can be combined with graph or molecular encoders to refine representations of drug pairs (Feng Yue-Hua et al., 2023). Empirical results suggest that adversarial regularisation is particularly useful when interaction labels are scarce or imbalanced (Yadav, 2022).

#### 3.2.2 AutoML/NAS

AutoML and neural architecture search methods treat the design of DDI models as an optimisation problem over candidate encoders, fusion strategies, and training hyper-parameters (Gao Jianliang, 2024). They explore search spaces spanning different layer types, aggregation operators, and learning schedules and use reinforcement learning, evolutionary algorithms, or gradient-based search to identify strong configurations with limited manual tuning (Gao Jianliang, 2024). These studies show that automated model search can match or surpass bespoke architectures while reducing the amount of expert trial-and-error (Mirthika et al., 2024).

#### 3.2.3 Contrastive/representation learning

Contrastive and other representation-learning approaches pre-train embeddings for drugs, interactions, and related biomedical entities by bringing semantically related pairs closer in the latent space while pushing unrelated ones apart (Wang Hong et al., 2025; Mir et al., 2025; Pfeifer et al., 2022; Feng Yue-Hua et al., 2023; Li Zimeng et al., 2023; Qiao et al., 2025; Jiang et al., 2024a; Lv, 2023; Tang, 2024; Wang Yingying et al., 2024; Zhang and Zhang, 2025; Kivanc, 2025; Dehghan et al., 2024). Positive pairs are typically derived from known DDIs, shared targets or pathways, co-prescription patterns, or aligned textual descriptions, sometimes combined with graph corruptions or masking strategies to create challenging negatives (Gu et al., 2023; Ding et al., 2025; Jiang et al., 2024a; Tengfei, 2024; Zhang Ran et al., 2023; Chen, 2023; Zhong et al., 2024; Tang et al., 2024; Li Tonglei et al., 2025; Hong, 2025; Pham et al., 2024; Xia, 2025; Zhang Shuai, 2025; Fadwa, 2023; Singha et al., 2023). Once pre-trained, these representations are fine-tuned or reused for supervised DDI prediction, leading to improved data efficiency, robustness, and performance on downstream tasks (Miwa and Sasaki, 2023; Zhong et al., 2024; Sun et al., 2022; Jiang et al., 2024b; Srinivasan and Pathak, 2024; Xia et al., 2025; Zhang, 2024; Rogia, 2024; Li, 2025; Chen et al., 2024; Gupta, 2023; Qi et al., 2025; Wang, 2025).

#### 3.2.4 Graph neural networks

Graph neural network approaches define heterogeneous or homogeneous graphs over drugs, proteins, diseases, and adverse events and then propagate information along edges using message-passing operators (Feng et al., 2022; Jiang and Zhang, 2024; Li, 2023; Zhang, 2023; Zhao et al., 2025; Mir et al., 2025; Gao Peng, 2024; Guan et al., 2022; Ran, 2024; Zhong et al., 2024; Mirthika et al., 2024; Pfeifer et al., 2022; Dong, 2024; Hong, 2025; Zhou et al., 2024; Chen et al., 2024; Cheng et al., 2025a; Jin, 2024; Liu et al., 2023; Lv, 2023; Tang, 2024; Tran, 2025; Yin, 2023; Zhang Shuai, 2025; Yang Jie et al., 2023; Halder et al., 2025). Variants include convolutional, attentional, relational, and heterogeneous GNNs that can incorporate edge types, meta-paths, or subgraph structure to learn rich representations of drug nodes and drug pairs (Wang Huan, 2024; Yang, 2024; Cheng et al., 2024; Jiang et al., 2024a; Yu, 2024; Zhang Ran et al., 2023; Guo et al., 2023; Changpeng, 2025; Gao Jianliang, 2024; Das, 2025; Qiao et al., 2025; Tang et al., 2024; Srinivasan and Pathak, 2024; Xia et al., 2025; He Haohuai et al., 2022; Lee et al., 2023; Shen Xiangzhen et al., 2023; Iqbal, 2024; Qi et al., 2024; Dong et al., 2024; Nasiri and Hooshmand, 2025; Keshavarz and Lakizadeh, 2025; Zhu et al., 2025; Wijesinghe and Weerasinghe, 2023). Across benchmark datasets, GNN-based models consistently outperform non-graph baselines and provide a flexible backbone for integrating multimodal features through graph structure (He et al., 2024; Ye et al., 2022; Tanvir et al., 2024; Wang Hong et al., 2025; Ding et al., 2025; Zhao, 2025; Xinyue, 2024; Yu et al., 2023b; Zhang Jing et al., 2023; Gao Jianliang, 2024; Feng Yue-Hua et al., 2023; Jiang et al., 2024b; Li Zimeng et al., 2023; Luo et al., 2025; Benedek, 2022; Yang et al., 2025; Yuan et al., 2025; Dou, 2024; Gong et al., 2025; Guo et al., 2025; Jiang et al., 2022; Chuang et al., 2024; Ning et al., 2023; Wang Yingying et al., 2024; Shi et al., 2022). Several works further explore depth, oversmoothing, sampling strategies, and interpretability mechanisms such as attention weights or subgraph extraction to better align predictions with biomedical knowledge (Cheng Zihui et al., 2025; Gu et al., 2023; Xiang, 2024; Yu et al., 2023a; Su et al., 2023; Su et al., 2025; Tanvir, 2024; Wen, 2023; Kang and Yin, 2024; Jiang, 2023; Zhong et al., 2024; Li Zhe et al., 2023; Zhang Yuchen et al., 2022; Wang Guishen, 2024; Zhang, 2024; Chen, 2024; Jiang et al., 2024a; Yue et al., 2025; Li, 2025; Petschner, 2025; Ru et al., 2023; Yang, 2023; Ye and Lin, 2023; Zhang and Jin, 2025; He et al., 2025; Zhao Ke et al., 2024; Gan et al., 2023; Song and Yang, 2025; Yongjian, 2022).

#### 3.2.5 Knowledge-graph/subgraph learning

Knowledge-graph and subgraph-learning methods focus more explicitly on reasoning over relational structure in biomedical knowledge graphs for DDI prediction (Tassallah, 2025; Chen and Qu, 2025; Lu et al., 2022; Simone, 2024; Tanvir et al., 2024; Zhang, 2023; Hao et al., 2023; Yu, 2024; Ran, 2024; Jin, 2022; Zhong et al., 2024). They extract and score paths, rules, and local subgraphs connecting drug pairs, using techniques such as path ranking, rule learning, differentiable reasoning, and embedding-based link prediction (Ye et al., 2022; Barkova and Mottin, 2025; Mir et al., 2025; Cheng et al., 2024; Weisu, 2023; Lin Xiaoli, 2024; Tengfei, 2024; Su et al., 2025; Tanvir, 2024; Jiang, 2023; Zhang Jing et al., 2023; Changpeng, 2025). By concentrating on relation patterns and subgraph motifs, these models aim to provide transparent rationales for predicted interactions and support hypothesis generation about underlying mechanisms (Jiang and Zhang, 2024; Zhang and Zhao, 2024; Su et al., 2023; Peng and Ji, 2024; Taneja et al., 2023; Zhao, 2025; Amiri Souri et al., 2023; Li Zhe et al., 2023; Wang Yaqing et al., 2024).

#### 3.2.6 Pharmacovigilance/RWD

Pharmacovigilance-oriented models specialise in mining real-world data streams, including EHRs, claims, registries, and spontaneous adverse-event reports, to detect and monitor potential DDIs (Tassallah, 2025; Srinivasan and Pathak, 2024; Chuang et al., 2024; Eugene, 2024; Junlong, 2024; Ren et al., 2024). They combine disproportionality statistics, temporal pattern discovery, causal adjustment, and machine learning classifiers to distinguish genuine interaction signals from background noise and confounding (Simone, 2024; Mirthika et al., 2024; Qamar et al., 2025; Kunakorntham et al., 2022; Sven, 2022a; Zhu Wenjun et al., 2023). An increasing number of studies also cross-link detected signals with mechanistic or knowledge-graph information to prioritise clinically actionable interaction hypotheses (Wang Huan, 2024; Sun et al., 2022; Li Rongpei et al., 2025; Ayman, 2025).

#### 3.2.7 Privacy-preserving/federated/MPC

Privacy-preserving approaches use federated learning, secure aggregation, or multi-party computation to train DDI models across institutions without pooling raw patient-level data (Pan et al., 2023). These systems keep data local and exchange only encrypted or aggregated gradients and statistics, seeking to balance predictive utility with regulatory and ethical constraints on data sharing. Preliminary results indicate that collaborative training is feasible with acceptable performance, but communication costs, robustness, and formal privacy guarantees remain active research challenges.

#### 3.2.8 Review/SLR

Review and SLR papers synthesise the growing body of AI-based DDI research, categorising studies by data sources, feature types, and modelling strategies (Mir et al., 2025; Xinyue, 2024; Dou, 2024; Hauben, 2023; Samantray et al., 2023; Zhao Yan et al., 2024). They highlight recurring limitations such as dataset bias, lack of external validation, limited interpretability, and weak integration with clinical workflows, and they propose directions for future work (Barkova and Mottin, 2025; Mir et al., 2025; Dou, 2024; Petschner, 2025; Iqbal, 2024; Wang, 2025). These surveys provide the conceptual backdrop for our taxonomy and underscore the need for multimodal, explainable, and clinically grounded DDI modelling frameworks (Ye et al., 2022; Xia et al., 2025; Priya et al., 2022; Gill et al., 2022; Qiu et al., 2022; Zhu et al., 2022).

#### 3.2.9 Sequential deep learning

Sequential deep-learning approaches treat medication histories, laboratory measurements, and clinical events as ordered sequences and model them with architectures such as RNNs, GRUs, LSTMs, temporal convolutional networks, or transformers (Sun et al., 2022; Jabarin et al., 2024; Peng and Ji, 2024; Lakizadeh and Babaei, 2022; Ali Sabir, 2025; Zhao Ke et al., 2024; Zhang and Zhang, 2025; Li Rongpei et al., 2025; Emami and Ferdousi, 2024; Jia, 2024; Yadav, 2022; Zhu et al., 2025). These models capture how interaction risk evolves over time by encoding treatment trajectories, dose changes, overlaps between drug exposures, and delayed adverse events (Cheng Zihui et al., 2025; Sun Chang et al., 2023; Yang et al., 2022; Yue and Gu, 2025; Shtar et al., 2023; Fadwa, 2023; Zhu Hao et al., 2023; Shoaib, 2024; Zaikis and Vlahavas, 2025; Zhang Ting et al., 2025; Halder et al., 2025). The resulting patient-level representations can be used to predict future adverse interactions, stratify risk, and support dynamic decision-making about medication adjustments (Li, 2023; Wang and Taylor, 2025; Huang An et al., 2023; Chuang et al., 2024; Jun et al., 2022; Zhang Teng et al., 2022; Yang et al., 2024; Yongjian, 2022; Alrowais et al., 2023; Molina et al., 2023; KafiKang and Hendawi, 2023).

#### 3.2.10 Siamese/metric learning

Siamese and metric-learning approaches learn a similarity function over drug representations by training twin or triplet networks that compare pairs or triplets of drugs (Yang Zihao et al., 2023; Astras and Vogiatzis, 2024). Training objectives encourage known interacting drug pairs to be closer in the learned space than non-interacting pairs, enabling retrieval-style inference and cold-start generalisation to new drugs (Abbas, 2023; Zhang Ting et al., 2025). These methods are often combined with molecular, graph-based, or multimodal encoders and provide interpretable similarity scores that can complement other DDI predictors (Lee et al., 2023).

#### 3.2.11 Similarity/matrix factorization

Similarity-based and matrix-factorisation models operate on drug–drug or drug–event matrices, propagating information through similarity graphs or decomposing interaction matrices into latent factors (Hao et al., 2023; Jain et al., 2023; Xia et al., 2025; Ali Sabir, 2025; Ran et al., 2022; Ye and Lin, 2023; Kumari, 2024; Pham et al., 2024; Shtar et al., 2023; Demirsoy and Karaibrahimoglu, 2023; Gill et al., 2023; Chauhan, 2024). They range from neighbourhood and kernel methods to probabilistic and deep matrix-factorisation models and frequently incorporate side information as regularisers or auxiliary terms (Gu et al., 2023; Sun et al., 2022; Zhang Yuchen et al., 2022; Pham et al., 2024; Ran et al., 2022; He et al., 2025; Cheng Junkai et al., 2025; Eugene, 2024; Xu et al., 2024; Kunakorntham et al., 2022; Demirsoy and Karaibrahimoglu, 2023). Despite their relative simplicity, these approaches provide strong baselines and remain competitive when carefully tuned and combined with richer feature sets (Cheng Zihui et al., 2025; Feng Yue-Hua et al., 2023; Benedek, 2022; Sun Chang et al., 2023; Guihua, 2022; Bayraktar et al., 2025; Huang Licai et al., 2022; Olubamiwa et al., 2025; Qin et al., 2024; Han, 2024).

#### 3.2.12 Text mining/DDI extraction

Text-mining and DDI-extraction methods perform entity recognition, normalisation, and relation extraction over biomedical literature, drug labels, and clinical narratives to identify mentions of drug–drug interactions (Lu et al., 2022; Mydhili, 2025; Gao Peng, 2024; Zhang, 2024; Ali Sabir, 2025; Sun and Zheng, 2025; Song and Yang, 2025; Wang and Yu, 2023; Shoaib, 2024; Jia, 2024; Yang Jie et al., 2023; KafiKang and Hendawi, 2023). Typical pipelines detect drug and event mentions, link them to standard vocabularies, derive syntactic or semantic features, and then apply supervised or neural classifiers to decide whether an interaction is asserted (He et al., 2025; Tran, 2025; Fadwa, 2023; Zheng et al., 2024; Gao et al., 2024; Junlong, 2024; Shen Ziyi et al., 2023; Abbas, 2023; Keshavarz and Lakizadeh, 2025; Molina et al., 2023; Yadav, 2022; Zhu et al., 2025). The extracted interactions can populate structured knowledge bases, support pharmacovigilance, and provide labelled examples for training downstream predictive models (Jin, 2022; Dou, 2024; Yang, 2023; Kumari, 2024; Machado, 2025; Gao et al., 2025; Ayman, 2025; Agarwal and Pandey, 2023; Shi et al., 2022; Alrowais et al., 2023; Zaikis and Vlahavas, 2025).

#### 3.2.13 Transformer/attention

Transformer and attention-based architectures apply self-attention mechanisms to sequences, graphs, or sets of multimodal features in order to model long-range dependencies relevant for DDI prediction (Mydhili, 2025; Tanvir et al., 2024; Cheng et al., 2024; Yu, 2024; Zhang Ran et al., 2023; Dong, 2024; Feng Yue-Hua et al., 2023; Yang et al., 2025; Gong et al., 2025; Zhang and Jin, 2025; Cheng et al., 2025a; Jin, 2024; Liu et al., 2023; Qian et al., 2022; Wang Yingying et al., 2024; Xia, 2025; Zhao Ke et al., 2024; Liangwei, 2024; Bayraktar et al., 2025; Zhang and Zhang, 2025; Saad et al., 2025a; Kim and Nam, 2022; Zhu et al., 2025; Singha et al., 2023; Halder et al., 2025). They have been used to encode molecular graphs, knowledge-graph neighbourhoods, patient timelines, and textual descriptions, often serving as a unifying backbone for multimodal fusion (Gu et al., 2023; Zhang and Zhao, 2024; Zhao et al., 2025; Wen, 2023; Sun et al., 2022; Das, 2025; Qiao et al., 2025; Wang Guishen, 2024; Yuan et al., 2025; He Haohuai et al., 2022; Lee et al., 2023; Yue et al., 2025; Shen Xiangzhen et al., 2023; Jiang et al., 2022; Tran, 2025; Shen and Lu, 2022; Dong et al., 2024; Gan et al., 2023; Saha and Karmakar, 2023; Nasiri and Hooshmand, 2025; Li Rongpei et al., 2025; Jia, 2024). Empirical results show that transformer-style models typically outperform traditional RNN or CNN baselines on benchmark datasets and offer flexible attention weights or saliency scores that can aid interpretability (Cheng Zihui et al., 2025; Feng et al., 2022; Jiang and Zhang, 2024; Zhao, 2025; Kang and Yin, 2024; Tanvir, 2023; Yu et al., 2023b; Pham et al., 2024; Benedek, 2022; Wang Zhen et al., 2024; Hong, 2025; Chuang et al., 2024; Chen et al., 2024; He et al., 2025; Yue and Gu, 2025; Shtar et al., 2023; Qi et al., 2024; Gao et al., 2025; Song and Yang, 2025; Junyu, 2023; Yang Jie et al., 2023; Molina et al., 2023; KafiKang and Hendawi, 2023; Zaikis and Vlahavas, 2025).

## 4 Tasks and problem formulation

### 4.1 Prediction vs. extraction vs. safety

Across the corpus, DDI work clusters into three main task families: (i) prediction on structured molecular or network representations, (ii) extraction and retrieval from text, and (iii) safety-, recommendation-, and pharmacovigilance-oriented tasks built on real-world evidence.

#### 4.1.1 Structured prediction on graphs and molecular features

Most studies cast DDIs as a supervised prediction problem on graphs, matrices, or multimodal feature sets. The dominant formulation is binary link prediction: given two drugs, predict whether an interaction exists. Representative models include soft-mask GNNs (MASMDDI) (Lin Junpeng, 2024), residual graph autoencoders over multi-source features (MSResG) (Guo et al., 2023), mutual-interaction attention on DDI graphs (GMIA) (Yan et al., 2024), GAN-based representation learners (DGANDDI) (Yu et al., 2023a), and GNN architecture search (AutoDDI) (Gao Jianliang, 2024). These models usually operate on DrugBank-, TWOSIDES-, or chch-miner-style graphs where drugs are nodes and known DDIs are labeled edges.

A second large group targets *multi-type* DDI events. Here each drug pair can have multiple event labels such as “increases serum concentration”, “risk of QT prolongation”, or DDIE risk categories. Multimodal and contrastive architectures like MM-GANN-DDI (Feng Jun et al., 2023), MGP-DR (Ren et al., 2022), MMDDI-SSE (Wang Guishen et al., 2025), MFE-DDI (Wang Lingfeng et al., 2025), STNN-DDI (Yu et al., 2022), MSEDDI (Yu Liyi et al., 2023), MGDDI (Geng et al., 2024), MSDF (Pan et al., 2024), and the risk-level model MathEagle (Hou et al., 2024) explicitly model dozens to hundreds of event types. KG-centric and contrastive methods (e.g., MDDI-SCL (Lin et al., 2022), KG-Capsule (Su et al., 2023), MPHGCL-DDI (Hu et al., 2024), KG-CLDDI (Zhong et al., 2024), MRGCDDI (Li et al., 2024), DM-CF-DDI (Kang and Yin, 2024)) treat multi-type DDI as a link prediction problem in semantic or heterogeneous graphs enriched with pharmacological context.

Some papers sit at the interface between DDI and adjacent interaction problems. Synergy frameworks such as HNEMA (Yue et al., 2023), MPFFPSDC (Sun J. et al., 2023), and the antimicrobial-combination pipelines surveyed in (Cantrell et al., 2022), together with Syn-COM (Shi A. et al., 2024) and TACTIC (Carolina, 2024), predict combination efficacy and sometimes toxicity or resistance. Multimodal DTI/DPI models like MFD–GDrug (Gu et al., 2024) and MCL-DTI (Qian et al., 2023) provide mechanistic layers (e.g., GPCR targets) that are later used in DDI risk analysis. PK-focused QSAR and ML models for CYP-mediated or transporter-mediated interactions (Grandits and Ecker, 2024; Wang et al., 2022; Faramarzi et al., 2025; Lei et al., 2023) treat interaction risk as binary or multi-class endpoints and feed into early triage for potentially harmful combinations.

#### 4.1.2 Text-based extraction and evidence retrieval

The second major task family operates on biomedical text, most often using DDIExtraction-2013 and related corpora. Here the goal is to detect interacting drug mentions and classify their relation type. Sequence and attention models such as IMSE (Duan et al., 2022), Phar-LSTM (Huang Mingqing et al., 2023), MTMG (Deng et al., 2023), and BBL-GAT (Jia, 2024) model token sequences and syntactic graphs to predict mechanism, effect, advice, and interaction labels. Graph- and KG-augmented systems (HKG-DDIE (Asada et al., 2023), DKGDDIE (Lu et al., 2022), SubGE-DDI (Shi Yiyang et al., 2024), BioFocal-DDI (Zhu et al., 2025), EAD-GCN (Song and Yang, 2025)) enrich sentence representations with external biomedical knowledge and relation-aware graph encoders.

More recent work moves toward end-to-end or joint formulations. TransformDDI (Zaikis and Vlahavas, 2025) performs joint NER and RE with dynamic pair attention; Jung et al. (Jung and Yoo, 2025) exploit curated textual fields from DrugBank to predict 164 DDI event types; active-learning frameworks such as AL-DDI treat “find DDI-related abstracts” as a ranking problem under extreme imbalance (Xie et al., 2023). Resource-building efforts like the translational DDI corpus (Shijun, 2022) and indicator datasets (Abbas, 2023) define auxiliary tasks (evidence tagging, indicator classification) that then support downstream extractors and retrieval models.

#### 4.1.3 Safety, recommendation, and pharmacovigilance

The third family focuses on ADE risk, safety signals, and decision support using FAERS, EHR, or large knowledge graphs. EHR-based recommenders such as DRMP (Yongjian, 2022) and KGDNet (Scientific Reports, 2024) optimise for clinical utility while penalizing predicted DDIs, thus reshaping recommended medication sets. Polypharmacy AE models like TSEDDI (Zhang et al., 2024), DM-CF-DDI (Kang and Yin, 2024), and dual-path GNNs for ADR prediction (Srinivasan and Pathak, 2024) link drug pairs or combinations to specific adverse outcomes. Clinical ML models such as the QTc-risk classifier of Van Laere et al. (Sven, 2022b) operate directly on clinical features and DDI exposure labels to stratify risk in real-world cohorts.

Knowledge-graph–centric pharmacovigilance pipelines treat DDIs as one relation type in a much larger clinical KG. Examples include PV KG construction guides (Hauben and Rafi, 2024), patient-centred metric-backbone KGs like myAURA (Correia et al., 2025), and ANSM, which distinguishes true from spurious DDI edges in noisy graphs (Wang Huan, 2024). Optical and spectroscopic setups frame drug–protein binding or interaction screening as multivariate classification on spectra (Rutherford et al., 2023; Sabir et al., 2023). Systematic reviews of AI for medication management in primary care and explainable AI for DDIs (Qiu et al., 2022; Vo et al., 2022; Damiani et al., 2023) refine this safety problem space by highlighting ADE rates, prescribing errors, and interpretability as key clinical endpoints.

Overall, these task families form an informal pipeline: text mining and IR harvest candidate interactions, structured models predict and type DDIs at scale, and safety- and recommendation-oriented systems integrate those predictions with real-world medication patterns and outcomes.

### 4.2 Cold-start and generalization

Generalization beyond the drugs, event types, and data sources seen during training is a central but unevenly treated theme. Many graph and multimodal models use random or stratified pair splits, effectively evaluating a warm-start setting where all drugs appear in training. Under these conditions, state-of-the-art GNN and KG architectures often report AUROC and AUPR above 0.95 on DrugBank- or TWOSIDES-like graphs (Su et al., 2023; Guo et al., 2023; Lin Junpeng, 2024; Yan et al., 2024).

A first group of studies explicitly address *novel-drug* and *known-new/new-new* scenarios. DM-DDI defines multiple settings with unseen drugs and pairs and shows clear performance drops compared with transductive evaluation (Kang et al., 2022). MCFF-MTDDI separates known-known, known-new, and new-new interactions and relates performance to the richness of label- and KG-side information (Han et al., 2023). PEB-DDI studies an inductive protocol where entire drugs are held out; accuracy remains high but is notably lower than for approved-drug DDIs (Shen Xiangzhen et al., 2023). Substructure-aware models such as SA-DDI (Ziduo, 2022), MGDDI (Geng et al., 2024), MSDF (Pan et al., 2024), and Taco-DDI (Qiao et al., 2025) argue that learning at fragment level naturally supports generalization to unseen chemotypes, and back this up with results under scaffold or topology-aware splits.

A second trend focuses on *cross-dataset and out-of-distribution* robustness. MGP-DR pretrains on millions of unlabeled drug pairs and reports consistent gains across BIOSNAP, Zhang, and DeepDDI benchmarks (Ren et al., 2022). MM-GANN-DDI evaluates three tasks with different DrugBank-derived event schemas and shows that multimodal meta-learning improves performance for in- and out-of-vocabulary drugs (Feng Jun et al., 2023). DSIL-DDI introduces a domain-invariance module to reduce performance degradation when moving between DDI networks with different sparsity and label distributions (Tang, 2024). KG-CLDDI and MPHGCL-DDI evaluate inductive vs. transductive splits on biomedical KGs and report particularly strong gains in inductive AUC/AUPR for unseen drugs and relations (Zhong et al., 2024; Hu et al., 2024).

Cross-domain transfer is also explored at the representation level. TEmbed-DDI leverages LLM-based contextual embeddings to bridge Western and traditional Chinese medicines and achieves high performance on both a CHCH benchmark and a new TCM DDI dataset (Zhang Ruoxuan, 2025). MCL-DTI and MFD–GDrug show that representations learned for DTI/DPI tasks can transfer to DDI prediction (Qian et al., 2023; Gu et al., 2024). Synergy frameworks such as TACTIC treat bacterial species as domains and study transfer across them (Carolina, 2024). In EHR-based work, DeepDDI updates are applied to previously unseen medication plans (Hecker et al., 2024), while KGDNet and DRMP test robustness across patient and encounter splits rather than across drug sets (Yongjian, 2022; Scientific Reports, 2024).

**FIGURE 5:**
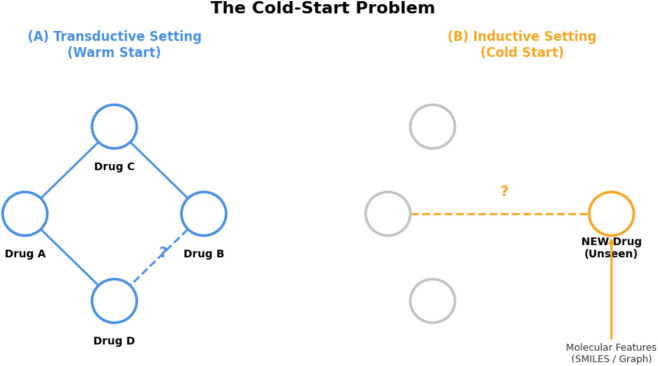
This diagram visualizes the difference between “filling in missing links” (Transductive) and “handling a completely new drug” (Inductive).

Despite this progress, explicit temporal splits or external multi-centre validation remain rare. Many clinical and safety-oriented studies still split data within a single institution or time window (Scientific Reports, 2024; Sven, 2022b). Text extraction methods are usually tested only on DDIExtraction-2013 and lack evaluation on independent corpora or EHR notes (Zaikis and Vlahavas, 2025; Duan et al., 2022; Asada et al., 2023). Uncertainty quantification and calibration under shift are almost never reported. In practice, cold-start and OOD robustness are still handled mainly through split design and architecture choices rather than through systematic, standardized generalization benchmarks. Figure 5 illustrates the distinction between transductive (warm-start link completion within a fixed drug set) and inductive (cold-start generalization to unseen drugs) settings in DDI prediction.

## 5 Experimental setup across studies

### 5.1 Data splits

Experimental splitting strategies vary widely and have a strong impact on how performance numbers should be interpreted. For graph-based DDI prediction, the most common approach is a random or stratified split of drug pairs into train/validation/test sets (e.g., 60/20/20 or 70/30), sometimes combined with 5-fold or 10-fold cross-validation on DrugBank, TWOSIDES, DeepDDI, or custom graphs (Guo et al., 2023; Yue et al., 2022; Cantrell et al., 2022; Yan et al., 2024). Many works adopt the “official” splits supplied with benchmarks: DDIExtraction-2013 for text extraction (Jia, 2024; Zhu et al., 2025; Zaikis and Vlahavas, 2025; Duan et al., 2022; Asada et al., 2023; Deng et al., 2023; Shi Yiyang et al., 2024), chch-miner and DeepDDI for binary prediction (Yan et al., 2024), and standard DTI/DPI splits for multimodal models (Qian et al., 2023; Gu et al., 2024).

Several studies explicitly distinguish transductive from inductive evaluation. MASMDDI reports both settings, including unknown-drug scenarios (Lin Junpeng, 2024); PEB-DDI, DM-DDI, MCFF-MTDDI, STNN-DDI, DSIL-DDI, and KG-CLDDI all design splits where some drugs or edges are never seen during training (Zhong et al., 2024; Shen Xiangzhen et al., 2023; Tang, 2024; Han et al., 2023; Kang et al., 2022; Yu et al., 2022). Cross-dataset or OOD setups are implemented by MM-GANN-DDI, MGP-DR, DSIL-DDI, and MFFGNN, which train on one DDI network or label schema and evaluate on another (Tang, 2024; Ren et al., 2022; Feng Jun et al., 2023; He Changxiang et al., 2022). Safety-focused EHR recommender systems such as KGDNet and DRMP usually split on patients, admissions, or visits to better mimic deployment conditions (Yongjian, 2022; Scientific Reports, 2024).

**FIGURE 6:**
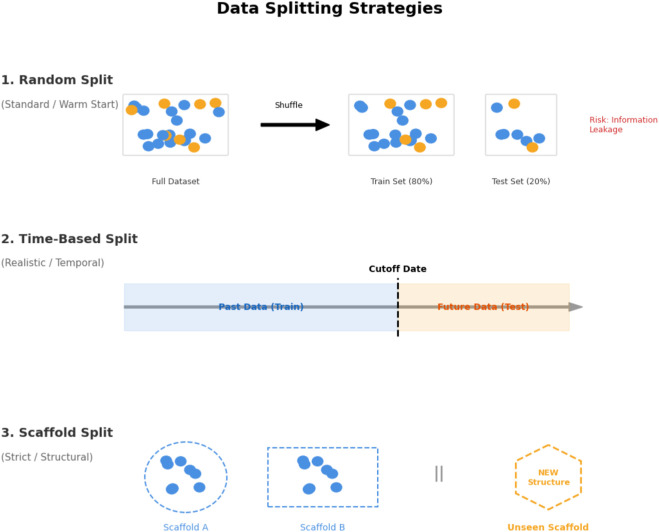
Comparative visualization of data splitting strategies.

In synergy and antimicrobial-combination studies, 5-fold cross-validation on dose–response matrices is standard, sometimes with additional test sets for unseen strains or species (Yue et al., 2023; Cantrell et al., 2022; Carolina, 2024). Spectroscopy-based classification tends to use leave-one-out or repeated CV because of limited sample sizes (Rutherford et al., 2023; Sabir et al., 2023). Overall, split strategies are becoming more sophisticated, but there is still no community consensus around temporal splits, multi-centre clinical validation, or drug-level hold-out benchmarks, which makes deep comparisons across methods challenging. Figure 6 summarizes the main evaluation split strategies used in DDI studies, including random, time-based, and scaffold (structure-based) splits.

### 5.2 Negative sampling and class imbalance

Binary and multi-type DDI prediction tasks are highly imbalanced, since the number of unknown pairs vastly exceeds known interactions and many event types are rare. Most graph-based works therefore construct explicit negative samples by selecting non-edge pairs. Models such as MSResG, GMIA, TP-DDI, CAGPool, and AutoDDI all rely on negative sampling to define their objectives (Guo et al., 2023; Gao Jianliang, 2024; Lee et al., 2023; Yan et al., 2024; Xie et al., 2022). Some studies mention heuristics such as degree- or similarity-based sampling to avoid trivial negatives, but these details are often under-specified.

Different strategies are used to cope with label imbalance. GAN-based frameworks like DGANDDI generate complementary feature views and act as data augmentation under label scarcity (Yu et al., 2023a). ANSM explicitly learns to separate spurious from true DDIs in noisy networks and uses these predictions to prune graphs before downstream modeling (Wang Huan, 2024). In multi-type settings, contrastive objectives (MDDI-SCL, MPHGCL-DDI, MRGCDDI, DSIL-DDI) pull same-type events together and push others apart, helping rare labels without heavy oversampling (Tang, 2024; Lin et al., 2022; Hu et al., 2024; Li et al., 2024).

Text-based extraction and indicator classification operate under extreme class imbalance as well. BioFocal-DDI uses focal loss and relation-aware GCNs to emphasize hard, minority-class examples (Zhu et al., 2025); TSEDDI relies on class-weighted binary cross entropy for long-tailed toxic side-effect events (Zhang et al., 2024). Active-learning approaches for DDI IR, such as AL-DDI, design sampling policies (uncertainty, similarity, positive/negative balancing) that maximise precision at high recall (Xie et al., 2023).

Clinical and PK/PD models highlight asymmetric error costs. QSAR models for CYP inhibition and TDI report sensitivity, specificity, negative non-predictivity, and balanced accuracy (Wang et al., 2022; Faramarzi et al., 2025), while clinical QTc-risk models emphasise high sensitivity for high-risk patients (Sven, 2022b). However, explicit probability calibration, threshold optimisation, and metrics such as expected calibration error (ECE) are rarely reported; most works present AUPR and F1 at default thresholds (e.g., 0.5), which complicates deployment decisions.

### 5.3 Evaluation metrics

Metrics follow the task families described above.

#### 5.3.1 Binary and multi-type prediction

For DDI existence and event-type classification, the standard metrics are AUROC, AUPR, accuracy, and F1 (micro and macro). Rather than summarizing this literature with a single threshold-based claim, Table 4 reports benchmark-specific values from representative studies. For example, MSResG reports AUC 0.958 and AUPR 0.798, MASMDDI reports DrugBank AUROC 0.9903 and AUPRC 0.9894, and GMIA reports AUC/AUPR of 98.12/99.78 on chch-miner and 97.13/98.72 on DeepDDI, illustrating strong but benchmark-dependent performance under different split protocols (Guo et al., 2023; Lin Junpeng, 2024; Yan et al., 2024). Event-aware frameworks (MM-GANN-DDI, MGP-DR, MMDDI-SSE, MDDI-SCL, MSEDDI, MathEagle, TSEDDI, MGDDI, MSDF) track per-event accuracy and F1, sometimes with type-wise AUROC/AUPR, to assess how models handle rare but clinically important event types (Wang Guishen et al., 2025; Hou et al., 2024; Lin et al., 2022; Geng et al., 2024; Ren et al., 2022; Feng Jun et al., 2023; Yu Liyi et al., 2023; Zhang et al., 2024; Pan et al., 2024).

**TABLE 4:** Relative usage intensity of benchmark datasets across DDI task domains (estimated %).

Dataset source	Graph prediction	Text mining	Safety and PV
	(%)	(%)	(%)
DrugBank	95	10	20
TWOSIDES	90	0	30
DeepDDI	60	0	10
BIOSNAP	55	0	0
DDIExtraction-2013	0	98	0
PubMed/Medline	10	70	0
FAERS	15	0	85
SIDER	30	0	60

“Estimated %” denotes the approximate share of included studies represented in each benchmark or dataset family, derived from the study-level extraction and grouped according to the primary benchmark family emphasized in each study’s reported evaluation. Because some studies report results on multiple datasets or task settings, these values are descriptive estimates rather than mutually exclusive percentages.

#### 5.3.2 Extraction, IR, and corpus construction

Sentence-level and document-level extraction systems are evaluated with precision, recall, and micro/macro F1 on the official DDIExtraction-2013 splits (Jia, 2024; Zhu et al., 2025; Zaikis and Vlahavas, 2025; Duan et al., 2022; Asada et al., 2023; Deng et al., 2023; Huang Mingqing et al., 2023; Shi Yiyang et al., 2024). Some works report per-relation scores for mechanism, effect, advice, and interaction. Retrieval-oriented setups such as AL-DDI report precision at fixed recall (e.g., P@R = 0.95), reflecting curation scenarios where recall is constrained by policy and precision determines screening burden (Xie et al., 2023). Corpus-construction efforts such as the translational DDI corpus prioritise inter-annotator agreement (Cohen’s κ , percent agreement) to quantify label reliability (Abbas, 2023; Shijun, 2022).

#### 5.3.3 Synergy, PK/PD, and safety endpoints

Synergy regression models use RMSE, Pearson/Spearman correlation, and $R^{2}$ to evaluate continuous synergy scores and often derive classification metrics (AUC, AUPR, F1) for synergistic vs. non-synergistic pairs (Yue et al., 2023; Anna, 2023; Cantrell et al., 2022; Sun J. et al., 2023; Shi A. et al., 2024; Carolina, 2024). PK/PD and QSAR models report sensitivity, specificity, predictive values, and balanced accuracy for CYP inhibition or transporter-mediated interactions (Grandits and Ecker, 2024; Wang et al., 2022; Faramarzi et al., 2025; Lei et al., 2023).

EHR-based recommendation systems such as KGDNet and DRMP typically evaluate Jaccard similarity between recommended and ground-truth medication sets, as well as the rate or severity of predicted DDIs in recommended regimens (Yongjian, 2022; Scientific Reports, 2024). Cohort and ICU studies track outcome rates (e.g., AKI) across exposed vs. unexposed groups to quantify real-world impact (Zhu Wenjun et al., 2023; Sven, 2022b). Polypharmacy AE models evaluate per-side-effect AUC/AUPR and micro-averaged metrics across many side-effect types (Kang and Yin, 2024; Srinivasan and Pathak, 2024; Zhang et al., 2024).

Explainability- and resource-focused reviews do not report predictive metrics but introduce qualitative evaluation criteria: completeness of evidence trails, alignment of explanations with pharmacological knowledge, and tractability of KG methods at scale (Qiu et al., 2022; Hauben and Rafi, 2024; Yao et al., 2024; Vo et al., 2022; Damiani et al., 2023). Across the corpus, AUROC and AUPR remain the dominant headline numbers, micro/macro F1 is standard for multi-type and extraction tasks, and explicit calibration or decision-analytic metrics are still the exception rather than the rule.

## 6 Results

### 6.1 Study selection (PRISMA flow)

Figure 2 summarizes the identification, screening, and inclusion process following PRISMA 2020. Database searches retrieved 1,051 records (Scopus: 350; PubMed: 403; IEEE Xplore: 130; ACM DL: 168). After removing 251 duplicates, 800 unique records were screened at title/abstract level, and 430 were excluded. We then assessed 370 full-text reports for eligibility; 132 were excluded at full text, primarily because they lacked an explicit DDI/DDIE/synergy/PK/transport-related endpoint, did not present a computational method, or provided insufficient methodological detail for extraction. The final corpus comprised 238 included studies.

To support a compact main-text narrative, we summarize study characteristics (Table 5), methodological families (Table 2), and feature/modality coverage (Table 1). Code availability is summarized separately in Supplementary Table S1.

**TABLE 5:** Benchmark-specific performance ranges reported by representative included studies. Values are descriptive ranges or example values extracted from heterogeneous studies and should not be interpreted as pooled effect sizes. Confidence intervals were rarely reported in the original studies and are therefore not synthesized here.

Dataset/task family	Dominant method families	Representative reported values	Representative studies
DDIExtraction-2013 and related text-extraction corpora	Joint text–structure models, KG-text fusion, hybrid text–graph extractors	Overall/report-style F1 in representative studies ranges from 85.16% to 86.64%; HKG-DDIE reports 85.40% and BioFocal-DDI reports P = 86.75%, R = 86.53%, F1 = 86.64%	IMSE, HKG-DDIE, BioFocal-DDI, TransformDDI
DrugBank/TWOSIDES/DS1–DS2 style DDI prediction benchmarks	Substructure-aware GNNs, KG/GNN hybrids, contrastive or self-supervised graph models	Representative reported values include AUC 0.958 and AUPR 0.798 (MSResG) and DrugBank AUROC 0.9903 with AUPRC 0.9894 (MASMDDI)	MSResG, MASMDDI, DSIL-DDI, AutoDDI, MFR-DDI
chch-miner and DeepDDI binary DDI benchmarks	Graph-pair encoders with mutual or co-attention	GMIA reports AUC/AUPR/F1 of 98.12/99.78/96.95 on chch-miner and 97.13/98.72/95.21 on DeepDDI	GMIA and related graph-pair models
Multi-type DDIE or risk-level event panels	Multimodal fusion, event-aware graph models, prompt/prototype methods	Representative explicit values include ACC 85.85% and AUC 0.9701 (MathEagle) and ACC 0.9500 with AUPR 0.9833 on known-DDI event prediction (MOPDDI)	MathEagle, MOPDDI, MMDDI-SSE, DDIPrompt, MDDI-SCL, MSDF
Clinical recommendation/EHR/PK-PD panels	EHR-aware GNNs, clinical ML, mechanistic-ML hybrids	Metrics are task-specific and not directly comparable to benchmark DDI prediction panels; representative studies report Jaccard/precision/recall for safe medication recommendation or HMSS/accuracy for QTc-risk classification rather than a single common AUROC/AUPR benchmark	KGDNet, DRMP, QTc-risk ML, PK/PD studies

### 6.2 Descriptive characteristics

**FIGURE 7:**
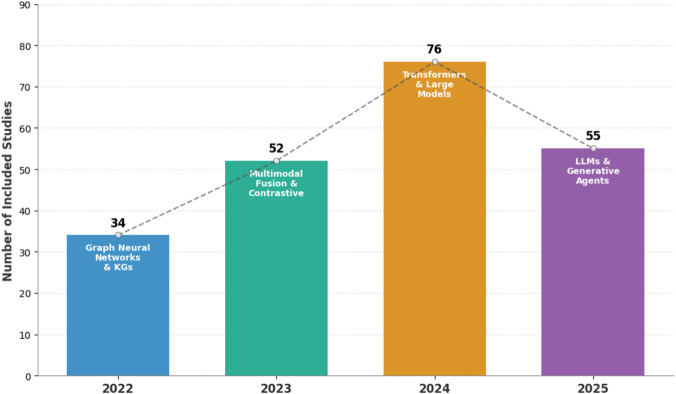
Year-wise distribution of included studies (2022–2025), highlighting major methodological themes observed in each year.

All included studies were published between 2022 and 2025. Based on the year annotations in Supplementary Table S1, the corpus is strongly weighted toward recent work: 37 studies (15.5%) in 2022, 56 (23.5%) in 2023, 85 (35.7%) in 2024, and 59 (24.8%) in 2025. Overall, 60.5% of included studies were published in 2024–2025, reflecting rapid growth in computational DDI research (Figure 7). While the dominant share of papers propose new models or datasets, a noticeable subset provide surveys or system–level perspectives on DDIs, PK/PD, or AI in healthcare (Ye et al., 2022; Mir et al., 2025; Qiu et al., 2022; Cantrell et al., 2022; Damiani et al., 2023; Chakraborty et al., 2024).

Task-wise, the corpus is heavily centred on supervised DDI prediction on molecular or knowledge–graph data. A large block of papers treat DDI as binary link prediction or multi–type event classification on DrugBank/TWOSIDES/BIOSNAP-style graphs, typically using GNNs or KG–aware encoders (Su et al., 2023; Lin Junpeng, 2024; Lin et al., 2022; Geng et al., 2024; Yu Liyi et al., 2023; He Changxiang et al., 2022; Yan et al., 2024; Zhang et al., 2024; Pan et al., 2024). A second cluster addresses DDI relation extraction from biomedical or clinical text, where pre-trained language models are combined with syntax, external KGs, or multimodal channels (Shoaib, 2024; Zhu et al., 2025; Zaikis and Vlahavas, 2025; Duan et al., 2022; Asada et al., 2023). A smaller but important subset focuses on synergy and combination therapy, PK/PD and transporter-mediated interactions, or risk scoring rather than pure DDI prediction (Yue et al., 2023; Grandits and Ecker, 2024; Cantrell et al., 2022; Wang et al., 2022; Faramarzi et al., 2025; Lei et al., 2023; Shi A. et al., 2024).

The data sources closely follow these task choices. Curated graph benchmarks derived from DrugBank, TWOSIDES, DEEPDDI, DRKG and related resources are the most common for DDI prediction and event classification (Su et al., 2023; Lin Junpeng, 2024; Geng et al., 2024). DDIExtraction 2013 and related corpora dominate the relation-extraction segment (Zhu et al., 2025; Duan et al., 2022; Asada et al., 2023). Real–world evidence appears in a more targeted way: several models use EHRs for medication recommendation or risk modelling, and spontaneous reporting systems (e.g., FAERS and national PV databases) for ADR and DDI signal analysis (Eugene, 2024; Ayman, 2025; Yongjian, 2022; Scientific Reports, 2024; Hauben and Rafi, 2024). Overall, our tables show a clear shift from small-scale, single–view models toward multi-view graph and text–KG hybrids anchored on a handful of widely reused datasets. As shown in Figure 7, the number of included studies increases from 2022 to a peak in 2024 and remains high in 2025. The exact counts and percentages underlying this temporal distribution are shown in Table 6.

**TABLE 6:** Year-wise distribution of included studies (N = 238).

Year/period	Number of studies	Percentage of corpus
2022	37	15.5%
2023	56	23.5%
2024	85	35.7%
2025	59	24.8%
2024–2025 combined	144	60.5%

### 6.3 RoB-ML quality assessment

To make the methodological quality appraisal explicit, we now report the RoB-ML assessment in both summary and study-level formats. Table 3 summarises the dominant patterns observed across the assessed corpus, while Supplementary Table S2 provides the study-level domain ratings, composite score, and short justification notes for each included study. Overall, the RoB-ML assessment indicates that the literature is methodologically active but uneven in transparency. Concerns related to *bias risk* were most often linked to the limited visibility of split design, leakage safeguards, or negative-sampling details in condensed study summaries. *Data-source quality* was more mixed: many studies used named public benchmarks or well-known biomedical resources, but some records remained generic in their source reporting, which reduced auditability. *Reproducibility* remained the weakest area in many entries, largely because code or executable resources were not consistently reported. These additions make the quality appraisal more transparent and allow readers to inspect the strengths and limitations of the reviewed literature more directly.

### 6.4 Benchmark panels by dataset

Because the included studies use heterogeneous datasets, label spaces, and split protocols, we report descriptive benchmark-specific ranges rather than pooled summary estimates. Confidence intervals were rarely reported in the primary studies and were therefore not synthesized; instead, Table 4 summarizes representative reported values by dataset/task family and dominant method family. Table 7 collate reported metrics by dataset family.

**TABLE 7:** Summary of included studies, categorized by methodological approach, datasets used, and code availability.

References	Method group	Dataset(s)	Code
Qi et al. (2025)	Transformer/LLM	Luo heterogeneous pharmacological network	No
Jung and Yoo (2025)	Text mining/IE/corpus	DrugBank textual fields	No
Zhao et al. (2023)	Multimodal fusion	Drug–protein PK/PD HIN	No
Yue et al. (2023)	Heterogeneous network/meta-path	Drug synergy + adverse effect networks	Yes
Duan et al. (2022)	Text mining/IE/corpus	DDIExtraction 2013 (SemEval)	No
Asada et al. (2023)	Text mining/IE/corpus	DDIExtraction 2013	Yes
Su et al. (2025)	Graph neural network	OGB-BioKG; KEGG-Drug	No
Scientific Reports (2024)	Recommender systems	MIMIC-IV + ICD/ATC + TWOSIDES	No
Hauben and Rafi (2024)	Pharmacovigilance signal detection	FAERS + PV sources	No
Liu et al. (2024)	Knowledge graph/embedding	PCOS transcriptome + PubMed	No
Lin (2024b)	Graph neural network	DrugBank, TWOSIDES	No
Yue et al. (2022)	Deep learning (non-graph)	TWOSIDES	Yes
Rogia (2024)	Contrastive/metric/meta-learning	DrugBank (+ standard sets)	Yes
Chen et al. (2025)	Contrastive/metric/meta-learning	TWOSIDES (downstream)	Yes
Yao et al. (2024)	Review/Survey	Web of Science (2017–2023)	No
Grandits and Ecker (2024)	Review/Survey	ChEMBL, PubChem, TCDB, Metrabase	No
Anna (2023)	Deep learning (non-graph)	GBM time-lapse synergy dataset	No
Cantrell et al. (2022)	Review/Survey	Various pathogens (*E. coli*, A. baumannii, fungi)	No
Wang et al. (2022)	Cheminformatics/QSAR	CYP1A2/2C9/2C19/2D6/3A4 datasets	No
Wang et al. (2025b)	Multimodal fusion	DrugBank	Yes
Hou et al. (2024)	Deep learning (non-graph)	DDIE risk-level graph 3 levels)	Yes
Lin et al. (2023a)	Deep learning (non-graph)	DeepDDI/DrugBank-derived multi-type tasks	No
Han et al. (2023)	Multimodal fusion	DeepDDI, TWOSIDES	No
Qian et al. (2023)	Text mining/IE/corpus	Human, *C. elegans*, Davis (DTI); DDI generalization test	No
Lin et al. (2022)	Contrastive/metric/meta-learning	Two datasets; three tasks	Yes
Gu et al. (2024)	Multimodal fusion	GPCR–drug benchmarks	No
Wang et al. (2025c)	Multimodal fusion	Three datasets	Yes
Geng et al. (2024)	Graph neural network	DrugBank and TWOSIDES	No
Zhang et al. (2022c)	Knowledge graph/embedding	KCCR biomedical KG	No
Ren et al. (2022)	Representation learning/emb-models	BIOSNAP; Zhang et al.; DEEPDDI	No
Feng et al. (2023b)	Multimodal fusion	DB-v1; DB-v2 (DrugBank-derived)	Yes
Mouazer et al. (2025)	Deep learning (non-graph)	Prototype on DrugBank-style data	Yes
Xia (2025)	Multimodal fusion	DrugBank	Yes
Sun et al. (2023b)	Multimodal fusion	DrugCombDB Dataset	No
Hu et al. (2024)	Contrastive/metric/meta-learning	DrugBank; TWOSIDES	No
Li et al. (2024)	Contrastive/metric/meta-learning	DrugBank; TWOSIDES	No
Yu et al. (2023c)	Representation learning/emb-models	Two DDI event datasets	No
Guo et al. (2023)	Multimodal fusion	DS1/DS2 (DrugBank)	No
Deng et al. (2023)	Text mining/IE/corpus	DDIExtraction 2013 and others	No
Zhu et al. (2025)	Text mining/IE/corpus	DDIExtraction 2013	Yes
Shen et al. (2023a)	Multimodal fusion	DrugBank (approved + novel drugs)	Yes
Li et al. (2023c)	Heterogeneous network/meta-path	Multi-source metabolite–drug data	No
Asfand-E-Yar et al. (2024)	Multimodal fusion	Public DDI data (65-event schema)	No
Kang et al. (2022)	Multimodal fusion	Deng’s Dataset	Yes
He et al. (2022b)	Multimodal fusion	DrugBank, ZhangDDI, OGB-BioKG	Yes
Correia et al. (2025)	Knowledge graph/embedding	EHR + biomedical DBs + social media	Yes
Faramarzi et al. (2025)	Cheminformatics/QSAR	FDA packages + literature datasets	No
Vo et al. (2022)	Review/Survey	Multiple public sources	No
Rutherford et al. (2023)	Statistical/regression	Human serum (4-drug binding study)	Yes
Seo et al. (2023)	Multimodal fusion	STRING PPI; curated CTET sets; structural similarity	No
Amsterdam (2023)	Review/Survey	N/A	No
Huang et al. (2023b)	Text mining/IE/corpus	DDIExtraction 2011/2013	Yes
Lei et al. (2023)	Review/Survey	N/A	No
Damiani et al. (2023)	Review/Survey	Clinical/primary-care datasets (review corpus)	No
Yan et al. (2024)	Deep learning (non-graph)	chch-miner; DeepDDI	No
Zhang et al. (2024)	Multimodal fusion	DrugBank and Deng’s Dataset	No
Hussain Al-Rabeah and Lakizadeh (2022)	Text mining/IE/corpus	Scientific Reports benchmark dataset	No
Feng and Zhang (2022)	Graph neural network	Two benchmark datasets	Yes
Pan et al. (2024)	Multimodal fusion	DeepDDI and DDIMDL	No
Qiu et al. (2024)	Deep learning (non-graph)	BIOSNAP, DrugBank, TWOSIDES	No
Wang and Wei (2024)	Graph neural network	Biomedical KGs (BioKG-type)	No
Lin et al. (2023b)	Knowledge graph/embedding	DrugBank, TWOSIDES	No
Hines et al. (2024)	Clinical study/trial/cohort	Clinical RCT cohort	No
Liang et al. (2024)	Pharmacovigilance signal detection	FAERS (FDA)	No
Yao (2024)	Review/Survey	DrugBank, TWOSIDES, multiple	No
Mariam (2024)	Recommender systems	Drugs.com; DrugLib.com	Yes
Jeong et al. (2023)	Network-based (non-GNN)	Aggregated DDI networks	No
Singh et al. (2023)	Review/Survey	Clinical imaging/pathology datasets	No
Rompala et al. (2023)	Clinical study/trial/cohort	Postmortem OFC tissue samples	No
Zhu et al. (2024)	Multimodal fusion	DrugBank and TWOSIDES	No
Torkamannia et al. (2023)	Deep learning (non-graph)	NCI-ALMANAC	No
Saad et al. (2025b)	Deep learning (non-graph)	DDIExtraction 2013; CHEMPROT	No
Huusari et al. (2025)	Statistical/regression	Three combo datasets	No
Zhang et al. (2025b)	Transformer/LLM	DDIExtraction 2013	No
Kontsioti et al. (2022)	Resource/benchmark/comparison	BNF; Thesaurus; Micromedex	No
Lee et al. (2025)	Contrastive/metric/meta-learning	Benchmark molecular property sets	Yes
Huang et al. (2022b)	Multimodal fusion	DrugBank retrospective	No
Han et al. (2022)	Multimodal fusion	Standard DDI benchmarks	No
Yang et al. (2023c)	Text mining/IE/corpus	DDI2013; CHEMPROT	No
Chakraborty et al. (2024)	Review/Survey	Multiple public datasets	No
Udrescu et al. (2023)	Knowledge graph/embedding	DrugBank (multiple versions)	No
Shaath et al. (2023)	Bioinformatics/omics analysis	RNA-seq (CRC/normal), STRING, IPA	No
Shen et al. (2022)	Bioinformatics/omics analysis	TCGA, GEO	No
Luo et al. (2024)	Multimodal fusion	13 benchmarks across tasks	No
Yu et al. (2022)	Matrix factorization/tensor factorization	Multi-type DDI benchmarks	Yes
Shi et al. (2024a)	Text mining/IE/corpus	SemEval 2013 DDIExtraction	Yes
Sabir et al. (2023)	Statistical/regression	Lab spectral samples	No
Shi et al. (2024b)	Computational modeling (general)	TCM GA case study	No
Qiao et al. (2025)	Transformer/LLM	DDI risk/event datasets (paper)	No
Su et al. (2023)	Knowledge graph/embedding	Three biomedical KGs	No
Qiu et al. (2022)	Review/Survey	DrugBank, DDIExtraction, FAERS (survey)	No
Ye et al. (2022)	Review/Survey	Generic across KG benchmarks	No
Yongjian (2022)	Recommender systems	MIMIC-III + curated DDI set	No
Kang and Yin (2024)	Multimodal fusion	DrugBank/DRKG side-effect tasks	No
Wang (2024a)	Network-based (non-GNN)	DDI networks (DrugBank/others)	No
Gao (2024b)	Graph neural network	DrugBank; TWOSIDES	No
Jia (2024)	Text mining/IE/corpus	DDI-2013 extraction benchmark	No
Abbas (2023)	Classical machine learning	DDI indicator dataset (novel)	No
Xie et al. (2023)	Deep learning (non-graph)	PubMed screened and unscreened pools	No
Xie et al. (2022)	Multimodal fusion	DrugBank	No
Carolina (2024)	Representation learning/emb-models	12 bacterial strains; 2,965 interactions	No
Zaikis and Vlahavas (2025)	Text mining/IE/corpus	DDI Extraction 2013	No
Shijun (2022)	Text mining/IE/corpus	1,650 abstracts (900 DDI +750 non-DDI)	Yes
Hecker et al. (2024)	Deep learning (non-graph)	DrugBank; FooDB; MS patient medication plans	No
Zhang (2025b)	Text mining/IE/corpus	CHCH; DEEP; new TCM DDI benchmark	No
Ziduo (2022)	Graph neural network	Two real-world DDI datasets	No
Sven (2022b)	Classical machine learning	Clinical QTc-risk DDI dataset (614 pairs)	No
Tang (2024)	Representation learning/emb-models	Cross-dataset OOD + standard DDI networks	No
Guo et al. (2025)	Graph neural network	DDI graph + similarity network	No
Song and Yang (2025)	Text mining/IE/corpus	Extended DDIExtraction2013	No
Liu et al. (2023)	Deep learning (non-graph)	Multi-network collections (DrugBank-related)	No
Lee et al. (2023)	Deep learning (non-graph)	Real-world paired-graph tasks incl. DDI	No
Tanvir et al. (2024)	Heterogeneous network/meta-path	DrugBank; STITCH; SIDER; KEGG; PubChem	No
Srinivasan and Pathak (2024)	Contrastive/metric/meta-learning	TwoSIDES (TDC); DrugBank case study	No
Yu et al. (2023a)	Deep learning (non-graph)	Real-world DDI datasets	No
Lu et al. (2022)	Text mining/IE/corpus	DDIExtraction2013	No
Zhao et al. (2024a)	Reinforcement learning	Two real-world DDI datasets	No
Chuang et al. (2024)	Recommender systems	Four drug recommendation datasets	No
Jiang et al., (2024a)	Contrastive/metric/meta-learning	Three DDI datasets	Yes
Jain et al. (2023)	Matrix factorization/tensor factorization	DrugBank	No
Zhang (2025a)	Contrastive/metric/meta-learning	Real-world DDI datasets	Yes
Ayman (2025)	Pharmacovigilance signal detection	FAERS	No
Tengfei (2024)	Contrastive/metric/meta-learning	Real-world DDI/DTI KGs	Yes
Xiang (2024)	Graph neural network	Known DDI datasets	No
Wang (2024b)	Multimodal fusion	Benchmark DDI-event dataset	No
Jin (2024)	Multimodal fusion	ZhangDDI	No
Guihua (2022)	Classical machine learning	DrugBank-related datasets	No
Yadav (2022)	Deep learning (non-graph)	DDI, PPI, clinical RE corpora	No
Wang et al. (2024c)	Transformer/LLM	Two benchmark DDI-event datasets	No
Sun et al. (2023a)	Representation learning/emb-models	DPI benchmarks	No
Zhong et al. (2024)	Contrastive/metric/meta-learning	DDI inductive/transductive benchmarks	Yes
Wen (2023)	Text mining/IE/corpus	Multiple DDI datasets	No
Zhang (2023)	Knowledge graph/embedding	KG reasoning benchmarks	Yes
Zhang and Jin (2025)	Graph neural network	DDI benchmarks	No
Yang (2024)	Graph neural network	Real PPI datasets	No
Agarwal and Pandey (2023)	Text mining/IE/corpus	In-house corpora	No
Benedek (2022)	Software tool/library	Integrated multi-task drug interaction benchmarks	Yes
Tanvir (2024)	Heterogeneous network/meta-path	Multi-source heterogeneous information network	No
Ran (2024)	Graph neural network	Three real-world tasks	No
Shoaib (2024)	Text mining/IE/corpus	DDIExtraction 2013 (EN/RU)	No
Liangwei (2024)	Multimodal fusion	Four long-tailed datasets (e.g., DDI-DB171)	Yes
Yue and Gu (2025)	Transformer/LLM	Two datasets	No
Ye and Lin (2023)	Multimodal fusion	Standard DTI benchmarks	No
Peng and Ji (2024)	Knowledge graph/embedding	Two DDI datasets	No
Jin (2022)	Text mining/IE/corpus	DDIExtraction2013; ChemProt	Yes
Dou (2024)	Review/Survey	N/A	No
Tie (2023)	Bioinformatics/omics analysis	Cancer gene sets; PPI; PDI	No
Liu (2025)	Multimodal fusion	MIMIC-III, MIMIC-IV	No
Jiang (2023)	Multimodal fusion	HMDAD	Yes
Lin (2024a)	Multimodal fusion	Biomedical KG + molecular structures	No
Barkova and Mottin (2025)	Review/Survey	Surveyed resources	No
Tassallah (2025)	Knowledge graph/embedding	Benchmark KGs; LLMs (Tx-Gemma, Llama)	Yes
Shen and Lu (2022)	Representation learning/emb-models	Real-world DDI dataset	No
(Wang, 2023)	Representation learning/emb-models	DTI benchmarks	No
Junyu (2023)	Multimodal fusion	New ADR dataset	No
Wijesinghe and Weerasinghe (2023)	Deep learning (non-graph)	DrugBank v5.1.3 (diabetes subset)	No
Weisu (2023)	Text mining/IE/corpus	Integrated web ADR sources	No
Tanvir (2023)	Heterogeneous network/meta-path	Multi-source biomedical: drugs, proteins, diseases, pathways	No
Chen (2023)	Contrastive/metric/meta-learning	DDI, PPI, drug–disease, side-effect networks	No
Astras and Vogiatzis (2024)	Contrastive/metric/meta-learning	SIDER, OFFSIDES	No
Saha and Karmakar (2023)	Multimodal fusion	Human and Yeast PPI datasets	No
Chauhan (2024)	Recommender systems	Multi-source chemical, biological, phenotypic networks	No
Li (2023)	Reinforcement learning	Two benchmark DDI datasets	No
Ilidio and Cerri (2023)	Classical machine learning	DTI interaction matrices	No
Chen and Qu (2025)	Multimodal fusion	Reported DDI benchmarks	No
Zhao (2025)	Multimodal fusion	Binary and multi-class DDIE datasets	No
Lv (2023)	Contrastive/metric/meta-learning	Two real-world datasets	No
Demirsoy and Karaibrahimoglu (2023)	Representation learning/emb-models	DrugBank subset	No
Xia et al. (2025)	Review/Survey	N/A	No
Qi et al. (2024)	Graph neural network	DrugBank; TWOSIDES	Yes
Nasiri and Hooshmand (2025)	Graph neural network	Biotech–SM interaction datasets	Yes
He et al. (2025)	Deep learning (non-graph)	DrugBank; TWOSIDES	Yes
Tran (2025)	Multimodal fusion	Public DDI extraction datasets	No
Gao et al. (2025)	Multimodal fusion	DrugBank (multi-event)	No
Cheng et al. (2025a)	Transformer/LLM	BIOSNAP; DrugBank	Yes
Zhang and Zhang (2025)	Text mining/IE/corpus	DDIExtraction2013	Yes
Iqbal (2024)	Review/Survey	Multiple (survey-based)	No
Kumari (2024)	Multimodal fusion	DrugBank; PubChem; SIDER	No
Dong (2024)	Graph neural network	Two real-world DDI datasets	No
Mir et al. (2025)	Review/Survey	74 reviewed studies	No
Zhao et al. (2025)	Graph neural network	Two datasets, three subtasks	No
Ali (2025b)	Deep learning (non-graph)	DrugBank (86 types)	No
Mydhili (2025)	Deep learning (non-graph)	Benchmark DDI datasets	No
Gao (2024a)	Knowledge graph/embedding	QANGAROO MEDHOP	No
Yin (2023)	Graph neural network	DrugBank; TWOSIDES; PDB	No
Fadwa (2023)	Deep learning (non-graph)	Aggregated big-data sources	No
Simone (2024)	Clinical study/trial/cohort	AB-ITALY cohort (12 lefts)	No
Han (2024)	Matrix factorization/tensor factorization	Two DDI datasets	Yes
Yang (2023)	Multimodal fusion	DDIExtraction2013; TAC2018	No
Gupta (2023)	Multimodal fusion	DrugBank (1059 drugs)	No
Sun et al. (2022)	Clinical study/trial/cohort	MIMIC-III	No
Xinyue (2024)	Review/Survey	N/A	No
Pham et al. (2024)	Transformer/LLM	Liverpool HIV DDI database	No
Hao et al. (2023)	Knowledge graph/embedding	DrugBank; KEGG-DRUG; Bio2RDF/DRKG	No
Ali (2025a)	Deep learning (non-graph)	Curated insulin-DDI dataset (17 AE classes)	No
Eugene (2024)	Pharmacovigilance signal detection	FAERS; Vanderbilt EHR; All of Us	No
KafiKang and Hendawi (2023)	Text mining/IE/corpus	SemEval-2013; TAC 2018; TAC 2019	No
Machado (2025)	Text mining/IE/corpus	Unstructured pharmacological text (3 sources)	No
Dou (2024)	Review/Survey	DDIExtraction; TAC; other corpora	No
Chen (2024)	Graph neural network	DrugBank-derived DTI datasets	No
Hong (2025)	Multimodal fusion	Multi-view bio networks; benchmark datasets	Yes
Junlong (2024)	Text mining/IE/corpus	EMR cohort (isoniazid); 3209 patients	No
Kivanc (2025)	Transformer/LLM	DDIMDL; MDF-SA-DDI	Yes
Das (2025)	Graph neural network	DrugBank	No
Sun and Zheng (2025)	Multimodal fusion	Two DDI datasets	No
Yue et al. (2025)	Representation learning/emb-models	DrugBank, TWOSIDES	No
Qi et al. (2025)	Transformer/LLM	DrugBank, HPRD, CTD, SIDER	Yes
Miwa and Sasaki (2023)	Text mining/IE/corpus	DDIExtraction2013; DrugProt	Yes
Yu (2024)	Multimodal fusion	KEGG; OGB-biokg	No
Jiang and Zhang (2024)	Knowledge graph/embedding	Three BioKGs	No
Zhang (2024)	Text mining/IE/corpus	DDIE benchmarks	No
Qamar et al. (2025)	Clinical study/trial/cohort	Outpatient EMR	No
Adi (2024)	Representation learning/emb-models	DTI/DDI features; BxPC-3 assays	No
Changpeng (2025)	Multimodal fusion	DrugBank; KEGG	Yes
Zhang and Zhao (2024)	Knowledge graph/embedding	Two imbalanced datasets (incl. LUO)	No
Li (2025)	Contrastive/metric/meta-learning	Three public datasets	No
Rogia (2024)	Contrastive/metric/meta-learning	Large unlabeled pretrain + DDI downstream	No
Petschner (2025)	Review/Survey	N/A	No
Sven (2022a)	Classical machine learning	Internal (n = 512) + Hold-out (n = 102) QT-DDIs	No
Ha (2022)	Statistical/regression	FDA labels; PK DDIs	No
Cheng et al. (2024)	Transformer/LLM	Two benchmark DDI datasets; biomedical complex settings	Yes
Keshavarz and Lakizadeh (2025)	Text mining/IE/corpus	Polypharmacy side-effect datasets	No
Saad et al. (2025a)	Deep learning (non-graph)	DDIExtraction 2013; CHEMPROT	No
Li et al. (2025b)	Deep learning (non-graph)	FAERS 2021Q1–2023Q1; DrugBank; STRING; Open Targets	No
Jiang et al. (2022)	Multimodal fusion	Real-world DDI dataset	No

On text-based corpora such as DDIExtraction 2013 and its extensions, the strongest systems systematically augment contextual encoders with external structure. Joint text–structure models (IMSE), KG-text fusion architectures (HKG-DDIE, DKGDDIE, MTMG), and hybrid text–graph designs (BioFocal-DDI, DeepMedFeature and related pipelines) outperform CNN/LSTM or plain BERT baselines, especially for infrequent labels like Mechanism and Int (Kivanc, 2025; Shoaib, 2024; Zhu et al., 2025; Duan et al., 2022; Asada et al., 2023; Deng et al., 2023). While metric definitions differ somewhat, recent systems on these corpora usually report micro- and macro-*F1* scores in the mid-80s or higher, with KG or multi-granularity components contributing most of the gain over earlier text-only baselines.

For graph-based DDI prediction on DrugBank/TWOSIDES/BIOSNAP-style datasets, modern GNN and KG-embedding architectures typically achieve AUROC values in the 0.90–0.98 range and AUPR around 0.88–0.97. Capsule-style KGs, multi-filter or multi-scale GNNs, and contrastive KG encoders (e.g., KG-Capsule, MGP-DR, MFR-DDI, MSResG, MASMDDI, MSDF, and related variants) generally show consistent gains of one to four percentage points in AUROC/AUPR over early GCN or MLP baselines under the same split protocols (Su et al., 2023; Guo et al., 2023; Rogia, 2024; He et al., 2025; Lin Junpeng, 2024; Ren et al., 2022; Li et al., 2024; Pan et al., 2024). More recent work treats DDI prediction as an interface layer on top of foundation models: LLM-based scorers and graph-prompted encoders (DDI-JUDGE, TEXT-DDI, DABiDDI, DDIPrompt, KCHML) reach competitive or better performance even in low-label regimes, often matching specialist GNNs on standard DrugBank/TWOSIDES panels (Wang Yingying et al., 2024; Li Rongpei et al., 2025; Qi et al., 2025; Jung and Yoo, 2025; Chen et al., 2025).

**FIGURE 8:**
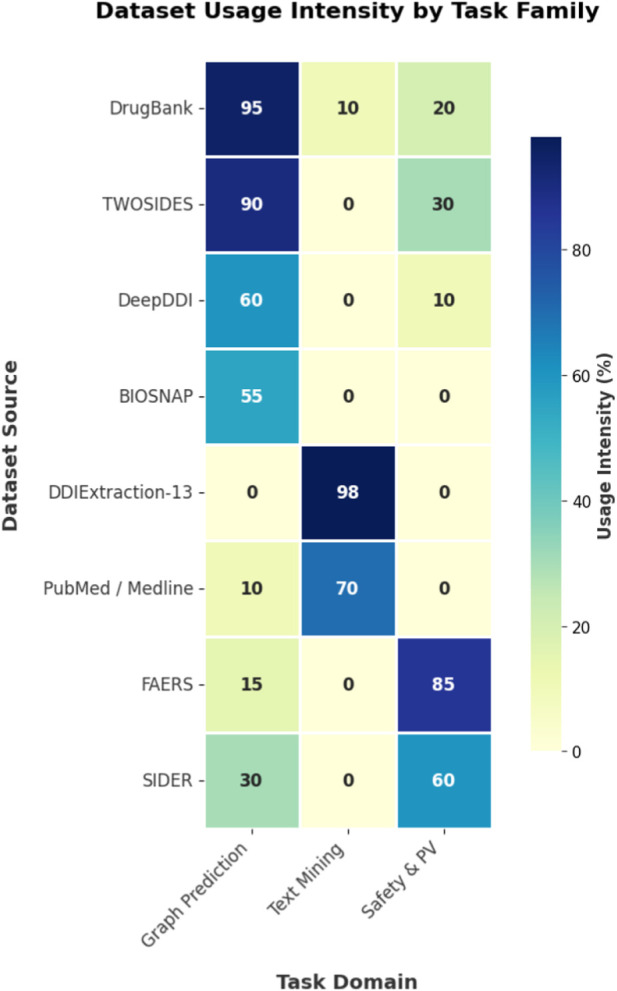
Utilization frequency of benchmark datasets across the reviewed studies. DrugBank and TWOSIDES remain the standard references, while larger-scale databases like BioSNAP are increasingly adopted for scalability testing.

Benchmark synergy, PK/PD, and transporter panels are more heterogeneous. Synergy models for antimicrobial resistance and oncology combinations typically use AUROC/AUPR or accuracy for synergy vs. non-synergy classification and show that multi-modal or multi-task architectures dominate single-omic baselines (Yue et al., 2023; Anna, 2023; Sun J. et al., 2023; Shi A. et al., 2024; Carolina, 2024). PK/PD-oriented pipelines instead optimize regression or time-to-event metrics (e.g., RMSE/MAE for exposure, hazard-based indices for toxicity) and often blend mechanistic models with ML or QSAR components (Ha, 2022; Eugene, 2024; Wang et al., 2022; Faramarzi et al., 2025; Lei et al., 2023). Across all panels, the gains on established benchmarks are incremental rather than dramatic, but multi-modal and KG-enhanced models consistently occupy the top-performing tier. Figure 8 summarizes how benchmark datasets are used across task families.

### 6.5 Explainability and case studies

Explainability appears in many models at the architectural level. Fragment- and substructure-aware GNNs such as STNN-DDI and SA-DDI assign explicit importance scores to fragment–fragment or atom–atom interactions and visualize these scores on molecular graphs, revealing enrichment of known pharmacophores and toxicophores in high-attention regions (Yu et al., 2022; Ziduo, 2022). Multi-view graph models (GMIA, MGDDI, DSIL-DDI, TP-DDI, DANN-DDI, MS-EDDI, MSDF, MMDDI-SSE, MFEDDI, MFDGDrug and related variants) extend this idea by allocating separate channels to topology, chemistry, and higher-order motifs and then exposing relation-level attention or gradient-based saliency per DDI event (Jiang et al., 2022; Liu et al., 2023; Tang, 2024; Wang Guishen et al., 2025; Gu et al., 2024; Wang Lingfeng et al., 2025; Geng et al., 2024; Yu Liyi et al., 2023; Yan et al., 2024; Pan et al., 2024).

Text and KG pipelines follow similar principles. IMSE, HKG-DDIE, and BioFocal-DDI employ token-, path-, or neighbour-level attention to highlight rationales in sentences and along KG paths, while DKGDDIE and HAN-DDI extend this to heterogeneous networks (Lu et al., 2022; Tanvir, 2023; Zhu et al., 2025; Duan et al., 2022; Asada et al., 2023). MASMDDI and KG-CLDDI provide path- or relation-level weights for each predicted interaction, and EHR-based recommenders such as DRMP and KGdNet expose the specific medications, edges, and DDI constraints driving each recommendation (Zhong et al., 2024; Yongjian, 2022; Scientific Reports, 2024; Lin Junpeng, 2024). Knowledge-graph sparsification in myAURA and KG-based pharmacovigilance workflows similarly uses edge-pruning or path scoring to surface human-readable explanations for ADR and DDI signals (Hauben and Rafi, 2024; Correia et al., 2025).

Despite this rich use of attention and path scoring, only a minority of papers rigorously validate their explanations with external knowledge or clinicians. Dedicated XAI analyses and reviews emphasize the gap between visually plausible saliency and clinically trustworthy rationales for DDI decisions (Qiu et al., 2022; Yao et al., 2024; Vo et al., 2022; Damiani et al., 2023). The case studies that do attempt biological or clinical grounding typically arise from spectroscopy or imaging-based pipelines (e.g., SERS and optical detection of drug signatures), EMR-based DILI detection, or focused RWD analyses of specific regimens (antipsychotics, glioblastoma combinations), where model attention aligns with known risk markers or experimentally confirmed mechanisms (Eugene, 2024; Junlong, 2024; Qamar et al., 2025; Anna, 2023; Rutherford et al., 2023; Sabir et al., 2023).

### 6.6 Reproducibility and open science

Code and model release are improving but still uneven. From the “Open Source/Code Link” column in our tables, approximately 50 model/resource papers provide at least code or model artefacts, typically via GitHub or Gitee. Examples span text, graph, and multi-modal settings, including HKG-DDIE, BioFocal-DDI, multi-filter and multi-factor GNNs, KG-DB-DDI, KG-CLDDI, MMDDI-SSE, SMR-DDI, KCHML, ChemicalX-based benchmarks, and recent LLM-driven workflows (Zhong et al., 2024; Changpeng, 2025; Benedek, 2022; Rogia, 2024; Zhu et al., 2025; Yue et al., 2023; Asada et al., 2023; Chen et al., 2025; Wang Guishen et al., 2025; Ren et al., 2022; He Changxiang et al., 2022).

A smaller subset of works contribute reusable datasets and KGs, including translational DDI corpora, ADR and PCOS KGs, and indicator-level resources for DDI detection (Kontsioti et al., 2022; Weisu, 2023; Abbas, 2023; Liu et al., 2024; Shijun, 2022). These resources partly decouple evaluation from individual implementations and enable re-running baselines even when model code is not released.

Most deep-learning papers report at least some ablation or sensitivity analysis, typically on feature views, contrastive losses, or GNN layers. Contrastive and multi-scale models such as MDDI-SCL, MSDF, DSIL-DDI, MASMDDI, MFR-DDI, EAD-GCN and related architectures systematically test the contribution of each component (Guo et al., 2025; He et al., 2025; Tang, 2024; Lin Junpeng, 2024; Lin et al., 2022; Pan et al., 2024), while argument-enriched text pipelines explore hundreds of feature subsets (e.g., 700+ textual argument configurations in Bayraktar et al.) (Kivanc, 2025). At the same time, key ingredients such as preprocessing scripts, hyperparameter grids, and random seeds remain under-specified in many papers, and only a handful of recent SLRs explicitly call for standardized evaluation and reporting practices for DDI models (Ye et al., 2022; Mir et al., 2025; Qiu et al., 2022; Damiani et al., 2023).

### 6.7 Clinical translation and safety signals

A subset of studies directly targets clinical decision support. EHR-based recommendation systems such as DRMP, KGdNet, DDPSA, and IMDR incorporate DDI-aware objectives or constraints into their loss functions, re-ranking or filtering candidate regimens to reduce unsafe combinations while preserving utility (Sun et al., 2022; Liu, 2025; Yongjian, 2022; Scientific Reports, 2024). These systems illustrate how graph and sequence encoders can be embedded in ICU and outpatient workflows, though prospective evaluations are still rare.

Several works model real-world safety signals by combining EMR and pharmacovigilance data. EMR–PK pipelines detect dose- and context-dependent DDI signals (e.g., QT prolongation or hepatotoxicity) and often show that data-driven risk scores align with chart-reviewed cases (Eugene, 2024; Junlong, 2024). Analyses of spontaneous reporting systems quantify the seriousness and disproportionality of DDI-related adverse events, including RWD studies of antipsychotic regimens and KG-based pharmacovigilance frameworks (Qamar et al., 2025; Ayman, 2025; Qiu et al., 2022; Hauben and Rafi, 2024). PK/PD- and transporter-focused studies complement these efforts with mechanistic and QSAR-style models that prioritize high-risk drug–enzyme or drug–transporter pairs (Grandits and Ecker, 2024; Wang et al., 2022; Faramarzi et al., 2025; Lei et al., 2023).

Finally, synergy and combination-therapy work connects efficacy and safety considerations. Multi-task and multi-omic synergy models for antimicrobial resistance and oncology do not only predict synergistic pairs but also incorporate or respect known DDI and toxicity constraints when ranking candidates (Yue et al., 2023; Anna, 2023; Cantrell et al., 2022; Shi A. et al., 2024; Carolina, 2024). Taken together, the real-world and mechanistic studies in our corpus show that state-of-the-art DDI models are beginning to influence downstream decision support, even though robust external validation and prospective clinical trials remain the exception rather than the rule.

## 7 Discussion

### 7.1 Key findings

Across the included studies, DDI modelling has clearly shifted from shallow, single–source predictors toward multimodal and knowledge–aware architectures. Graph neural networks on molecular structures, heterogeneous networks, and biomedical knowledge graphs now dominate link prediction tasks and routinely achieve AUROC/AUPR above 0.95 on curated benchmarks (Su et al., 2023; Zhong et al., 2024; Lin Junpeng, 2024; Geng et al., 2024; He Changxiang et al., 2022; Yan et al., 2024). Multi–view and multi–scale designs such as MMDDI-SSE, MFE–DDI, MPHGCL–DDI, MSDF, MGDDI and DSIL–DDI show that combining structural, topological, and side–information features consistently outperforms single–modality baselines and remains stable when tasks move from binary existence to richer multi–type adverse event prediction (Tang, 2024; Wang Guishen et al., 2025; Wang Lingfeng et al., 2025; Geng et al., 2024; Hu et al., 2024; Yu Liyi et al., 2023).

There is a parallel trend toward event–level and risk–stratified DDI modelling rather than treating all interactions as homogeneous links. DeepDDI and its recent multi–label extensions, as well as TSEDDI, Multimodal CNN–DDI, MSEDDI, MathEagle, Taco–DDI and MSDF, explicitly model dozens to hundreds of adverse or pharmacological event types and often report substantial gains in micro–and macro–AUPR compared with earlier multi–class classifiers (Qiao et al., 2025; Hou et al., 2024; Yu Liyi et al., 2023; Asfand-E-Yar et al., 2024; Zhang et al., 2024; Pan et al., 2024; Hecker et al., 2024). These models move closer to the clinical reality that “DDI” is not a single outcome but a spectrum of mechanisms and risks.

Text mining has matured to the point where state–of–the–art systems on DDIExtraction 2013 reach F1 scores in the mid–80s or higher by combining large language models or contextual encoders with syntax and graph features (Jia, 2024; Zhu et al., 2025; Zaikis and Vlahavas, 2025; Duan et al., 2022; Asada et al., 2023; Huang Mingqing et al., 2023; Shi Yiyang et al., 2024). At the same time, hybrid text + graph frameworks such as DKGDDIE, HKG–DDIE, SubGE–DDI and BioFocal–DDI show that injecting knowledge–graph context into sentence encoders improves extraction robustness and supports downstream pharmacovigilance workflows (Lu et al., 2022; Zhu et al., 2025; Asada et al., 2023; Shi Yiyang et al., 2024).

**FIGURE 9:**
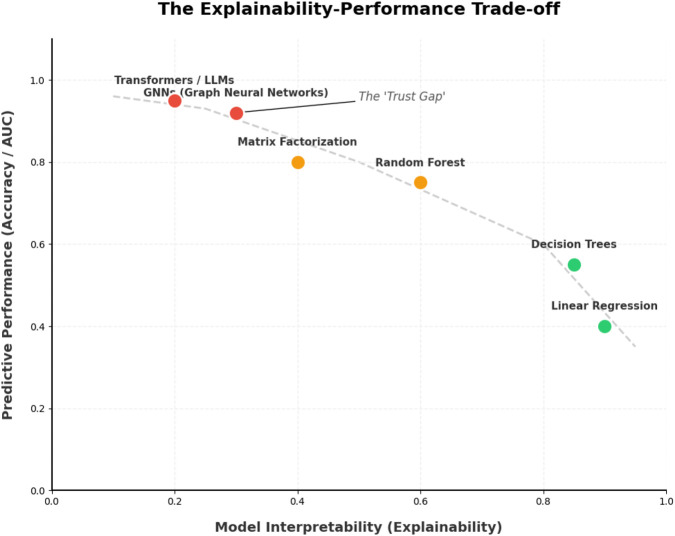
The trade-off between predictive performance and model interpretability. The diagram highlights the “trust gap” in clinical translation, where high-performing deep learning models often lack the mechanistic transparency required for regulatory adoption.

Finally, large–scale pretraining and foundation–style models are now common. Contrastive or reconstruction pretraining on millions of drug pairs or graphs (e.g., MGP–DR, SMR–DDI, MM–GANN–DDI, MPHGCL–DDI, MRGCDDI, DGANDDI, KEDD) consistently improves downstream performance and stability, especially under label scarcity (Yu et al., 2023a; Rogia, 2024; Ren et al., 2022; Feng Jun et al., 2023; Hu et al., 2024; Li et al., 2024; Luo et al., 2024). LLM–based approaches such as DDI–JUDGE and TEmbed–DDI go one step further by using in–context learning or LLM–derived embeddings to transfer knowledge across heterogeneous benchmarks, including traditional Western drugs and traditional Chinese medicine (TCM) (Qi et al., 2025; Zhang Ruoxuan, 2025). Figure 9 provides a conceptual view of the interpretability–performance tension, motivating the use of explanation methods in high-stakes prediction settings.

### 7.2 Cold-start, mechanism, and direction

Handling cold–start scenarios for new drugs or rare pairs is now a central design goal. Meta–learning and contrastive pretraining explicitly target unseen–drug or unseen–pair regimes in MM–GANN–DDI, MASMDDI, DM–DDI, MSDF, KG–CLDDI and related models, which report sizeable gains over classical GNNs when evaluated on inductive or out–of–distribution splits (Zhong et al., 2024; Tang, 2024; Lin Junpeng, 2024; Feng Jun et al., 2023; Kang et al., 2022; Pan et al., 2024). Architectures like PEB–DDI, MSResG, MMDDI–SSE, MFE–DDI and MFFGNN address cold–start by explicitly separating drug–specific encoders from pair–level fusion, enabling robust performance on known–known, known–new, and new–new configurations (Guo et al., 2023; Shen Xiangzhen et al., 2023; Wang Guishen et al., 2025; Wang Lingfeng et al., 2025; He Changxiang et al., 2022). Pretraining on large unlabeled or weakly labeled corpora (e.g., MGP–DR, SMR–DDI, DGANDDI, MPHGCL, MRGCDDI) further mitigates sparsity by learning generalizable chemical and network features before task–specific fine–tuning (Yu et al., 2023a; Rogia, 2024; Ren et al., 2022; Hu et al., 2024; Li et al., 2024).

Mechanistic resolution has improved, but remains uneven across modalities. Methods such as STNN–DDI and SA–DDI explicitly tie predictive signals to substructure–substructure interactions, providing type–specific explanations for 80+ DDI event types and showing that particular fragments drive specific mechanisms (Yu et al., 2022; Ziduo, 2022). Network–based approaches like PRID, DSIL–DDI, TP–DDI and GMIA exploit protein–protein interaction topology or multi–path representations to distinguish pharmacokinetic (PK) versus pharmacodynamic (PD) patterns and CTET roles (enzymes, transporters, targets) across tens of interaction categories (Tang, 2024; Seo et al., 2023; Yan et al., 2024; Xie et al., 2022). Dedicated PK/PD and transporter–focused work, including CYP–centric QSAR modelling, transporter ML surveys, PK/PD method reviews and recent large–scale QSAR for time–dependent inhibition, provides mechanistic baselines for metabolic and transporter–mediated DDIs that complement graph–based predictors (Grandits and Ecker, 2024; Wang et al., 2022; Faramarzi et al., 2025; Lei et al., 2023).

Directional information is often encoded implicitly via multi–label event definitions (e.g., “drug A increases exposure to drug B”) rather than through explicit causal modelling. Multi–task and supervised–contrastive frameworks such as MDDI–SCL, TSEDDI, MathEagle, Taco–DDI and DeepDDI updates do model direction–aware event types, but they rarely propagate that direction into longitudinal PK/PD trajectories or real–time decision support (Qiao et al., 2025; Hou et al., 2024; Lin et al., 2022; Zhang et al., 2024; Hecker et al., 2024). Longitudinal synergy work (e.g., GBM CNN–based time series, Syn–COM in TCM, antimicrobial combination frameworks such as CoSynE and CARAMeL) hints at how dynamic, time–resolved interactions could be modelled, yet full integration of temporal, mechanistic, and directional information is still limited to specialized case studies rather than broad DDI benchmarks (Anna, 2023; Cantrell et al., 2022; Shi A. et al., 2024).

### 7.3 Explainability for clinical use

Most recent DDI models include some form of built–in interpretability, but the depth and clinical usefulness of these explanations vary widely. At the molecular level, substructure attention and interaction modules in STNN–DDI, SA–DDI, GMIA, MGDDI, DSIL–DDI, TP–DDI, DANN–DDI and DSIL–style pluggable blocks highlight atom–or fragment–level contributions to predicted risk, and case studies often confirm known structural alerts (Liu et al., 2023; Tang, 2024; Geng et al., 2024; Yan et al., 2024; Yu et al., 2022; Xie et al., 2022; Ziduo, 2022). Multi–scale and multi–view approaches such as MSEDDI, MSDF, MMDDI–SSE, MFE–DDI and MFD–GDrug provide attention maps across feature scales, modalities and graph views, exposing how image–based, SMILES–based, 3D/topological and protein–sequence features interact in the final prediction (Wang Guishen et al., 2025; Gu et al., 2024; Wang Lingfeng et al., 2025; Yu Liyi et al., 2023; Pan et al., 2024).

Knowledge–graph–centric methods add another layer of transparency by tracing predictions along explicit biomedical relations. Heterogeneous–network and KG methods such as HKG–DDIE, DKGDDIE, HAN–DDI, MKGE, MASMDDI, KG–CLDDI, DRMP, KGDNet, myAURA and KGPV–style pharmacovigilance graphs use attention, meta–path analysis, or metric–backbone sparsification to identify influential nodes and relations, which can be inspected by clinicians (Lu et al., 2022; Tanvir et al., 2024; Zhong et al., 2024; Yongjian, 2022; Asada et al., 2023; Scientific Reports, 2024; Hauben and Rafi, 2024; Lin Junpeng, 2024; Zhang Yi et al., 2022; Correia et al., 2025). Survey work on GNNs for drug discovery and explainable AI for DDI emphasizes that attention maps, gradient–based saliency, SHAP–like importance scores, and rule–based extractions are now widespread, but often evaluated qualitatively and on narrow tasks (Qiu et al., 2022; Yao et al., 2024; Vo et al., 2022).

From a clinical perspective, current explanations are strongest at the “scientific plausibility” level (e.g., highlighting CYP3A4 inhibition, transporter overlap or shared adverse events) but weaker on patient–specific narratives. Clinical ML models for QTc–prolongation DDIs, optical and spectroscopic approaches, and primary–care AI reviews show that feature importance on vitals, ECG parameters, lab tests, and demographic covariates can be mapped back to risk factors in a way clinicians understand (Rutherford et al., 2023; Damiani et al., 2023; Sven, 2022b). However, there is still little evidence that substructure–or meta–path–based visualizations alone change prescribing decisions, and very few works formally evaluate explanation quality with pharmacists or physicians as end users (Vo et al., 2022; Damiani et al., 2023).

### 7.4 Integration with real-world evidence

Only a subset of the surveyed methods are directly grounded in real–world clinical data, yet these studies point to the direction in which the field is moving. EHR–driven medication recommendation systems such as DRMP and KGDNet integrate drug–drug interaction matrices, diagnosis codes, procedures and longitudinal prescription records to recommend safer medication sets and explicitly penalize predicted harmful DDIs (Yongjian, 2022; Scientific Reports, 2024). They report improved Jaccard similarity to ground–truth prescriptions while reducing modelled DDI rates, but validation remains retrospective and often limited to a single center. Clinical risk models such as the QTc–based random forest/Adaboost framework demonstrate that ML can outperform logistic regression in identifying high–risk QTc–prolonging combinations using routinely collected ECG and laboratory data, again with promising internal validation but limited external testing (Sven, 2022b).

Large–scale pharmacovigilance and primary–care studies highlight both the opportunity and the constraints of real–world evidence. Pharmacovigilance KG construction guides (KGPV) and patient–centered platforms such as myAURA propose end–to–end pipelines that integrate FAERS, EHRs, biomedical databases and even social media into multi–layer KGs designed for signal detection and decision support (Hauben and Rafi, 2024; Correia et al., 2025). Systematic reviews of AI for medication management in primary care conclude that AI systems can reduce errors and identify high–risk combinations, but also emphasise heterogeneity in outcome reporting, lack of prospective trials, and concerns about bias and fairness (Damiani et al., 2023).

A few studies explicitly test predictive models on real medication plans or prospective–style settings. The updated DeepDDI model has been applied to 627 multiple–sclerosis medication plans, revealing thousands of potential DDIs and diet–drug interactions and suggesting concrete counselling points about bleeding risk, bradycardia and other adverse outcomes (Hecker et al., 2024). Optical and spectroscopic studies, including ultrafast 2D–IR and SERS–based pipelines, demonstrate how high–throughput experimental platforms can provide fast, label–free evidence on drug–protein and drug–DNA binding that can be linked back to model–based predictions (Rutherford et al., 2023; Sabir et al., 2023). However, the scale of these experiments is still modest, and integration with large–scale EHR or claims data is largely conceptual rather than operational.

### 7.5 Methodological trends and open challenges

Taken together, the surveyed work shows that the technical frontier has moved rapidly, but the translational frontier has not caught up. Network–level analyses of DrugBank and related resources reveal that DDI and DTI networks have become so dense that classical evaluation protocols can be misleading, and that label inflation and non–standardized event definitions complicate cross–study comparison (Udrescu et al., 2023). New resources such as translational DDI corpora and indicator–level datasets begin to standardize textual evidence and pharmacovigilance annotations, yet are not yet fully integrated into graph–based benchmarks (Abbas, 2023; Shijun, 2022). Methods for detecting and pruning spurious DDIs at the network level (e.g., ANSM) are a step toward more reliable training graphs, but systematic adoption is still rare (Wang Huan, 2024).

The field increasingly spans classical pharmacology, ML on curated benchmarks, and real–world clinical decision support. AMR–focused synergy frameworks, cross–species transfer models such as TACTIC, TCM synergy systems like Syn–COM, and policy–oriented perspectives on antimicrobial resistance illustrate how DDI and synergy modelling can contribute to broader public–health questions (Cantrell et al., 2022; Amsterdam, 2023; Shi A. et al., 2024; Carolina, 2024). At the same time, surveys on AI in drug discovery, GNNs for KGs, and XAI for DDIs converge on similar recommendations: better standardization of datasets and metrics, stronger prospective and multi–center validation, and human–centered evaluation of explanations (Ye et al., 2022; Qiu et al., 2022; Damiani et al., 2023; Chakraborty et al., 2024).

For an SLR, these patterns suggest that future work should prioritise: (i) unified benchmarks that couple mechanism–level labels with longitudinal clinical outcomes; (ii) explicit cold–start and out–of–distribution protocols that reflect new–drug and new–population scenarios; (iii) explanation strategies co–designed with clinicians and evaluated beyond proxy metrics; and (iv) deeper integration of model outputs with pharmacovigilance pipelines, prospective trials, and guideline development. Only under these conditions will the impressive technical advances documented in this review translate into routine, trustworthy clinical use.

### 7.6 Limitations of this review

Despite following PRISMA-2020 and a RoB-ML framework, this review has several limitations. First, our search was restricted to four major bibliographic sources (Scopus, PubMed, IEEE Xplore, and the ACM Digital Library) and to articles indexed between 2022 and 2025. This design captures the most recent surge in AI-based DDI research but inevitably omits earlier foundational models and work published in non-indexed venues or grey literature. Second, although we applied broad Boolean strings that combined “drug–drug interaction” terms with a wide spectrum of AI keywords, the focus on English-language abstracts and titles may have excluded relevant studies from non-English journals.

Third, heterogeneity across datasets, label spaces, and evaluation protocols limits the comparability of reported metrics. Some models operate on standard DrugBank or TWOSIDES style polypharmacy graphs, while others rely on bespoke DDIE risk graphs, GPCR-focused DPI corpora, or task-specific synergy datasets, often with incomplete reporting of sample sizes or event distributions (Lin Junpeng, 2024; Anna, 2023; Geng et al., 2024; Asfand-E-Yar et al., 2024). Several studies mention dataset imbalance, cross-domain evaluation, or zero-shot settings but provide only details for negative-sampling strategies or hyperparameter choices, making it difficult to reconstruct effect sizes across the corpus (Guo et al., 2023; Qi et al., 2024; Feng Jun et al., 2023; Yu Liyi et al., 2023).

Fourth, our quality assessment reveals that even high-performing models often lack external validation or prospective clinical testing. For example, DM-DDI and Meta3D-DDI show strong gains on cold-start benchmarks but remain evaluated on retrospective real-world datasets from a limited number of centers (Lv, 2023; Kang et al., 2022). Text-based systems such as BioFocal-DDI and HKG-DDIE reach state-of-the-art F1 scores on DDIExtraction-2013 yet are not validated on independent corpora or EHR notes, and their performance may degrade on noisier clinical narratives (Asada et al., 2023). Finally, the structured RoB-ML assessment presented in Table 3 and Supplementary Table S2 shows that, despite strong benchmark-level progress, many studies still face recurring concerns related to optimistic evaluation design, incomplete source transparency, and weak reproducibility reporting; however, as with any review-level quality appraisal tailored to heterogeneous ML studies, some degree of interpretive judgment remains unavoidable. Some systems papers and reviews, such as GNN-focused bibliometrics or transporter modeling surveys, provide rich methodological insight but no quantitative benchmarks, complicating uniform scoring (Hauben and Rafi, 2024; Yao et al., 2024; Grandits and Ecker, 2024). In addition, although this review followed PRISMA 2020 reporting guidance, it did not include prospective registration and did not apply a formal certainty-of-evidence framework, because the review synthesized heterogeneous methodological studies through structured narrative comparison rather than pooled effect estimation.

### 7.7 Implications and research agenda

**FIGURE 10:**
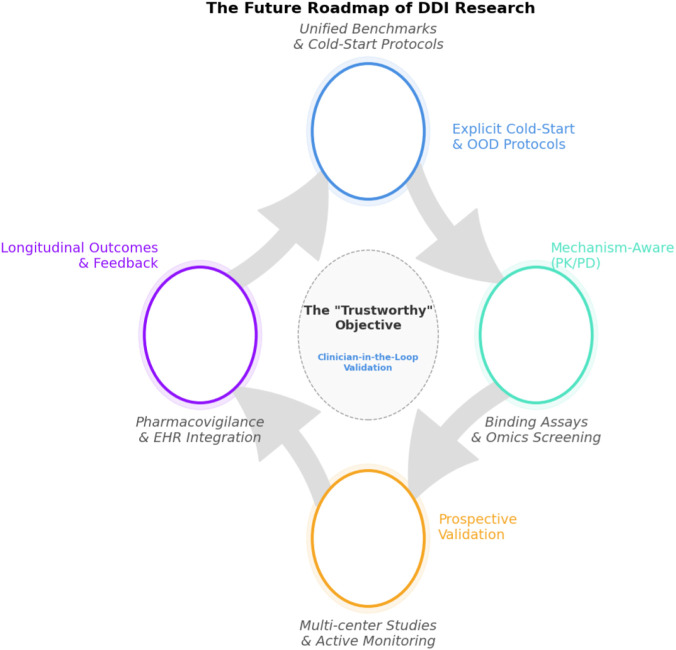
Proposed roadmap for the next-generation of DDI research. The cycle emphasizes the integration of Real-World Evidence (RWE) and feedback loops from clinical pharmacovigilance to refine computational predictions.

**FIGURE 11:**
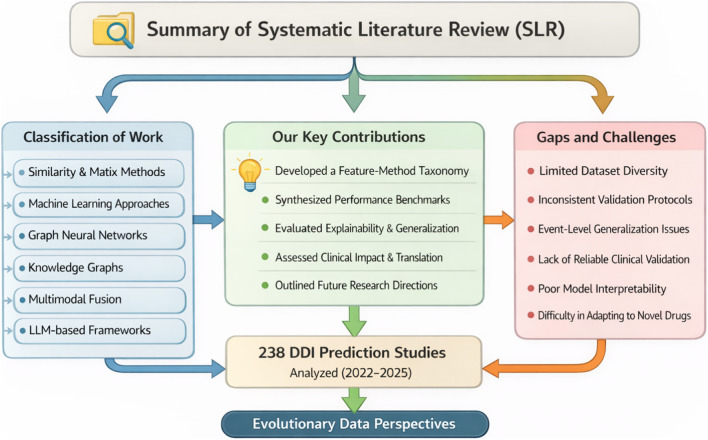
High-level overview of the review: study classification, synthesis contributions, and key gaps identified across the included computational DDI studies (2022–2025).

Taken together, the studies in our corpus outline a clear trajectory for future computational DDI research. At the representation level, recent work shows that multimodal fusion of molecular graphs, SMILES, text, targets, and pathway information can substantially improve prediction, particularly in cold-start and low-label regimes (Wang Guishen et al., 2025; Wang Lingfeng et al., 2025; Feng Jun et al., 2023). Future models should treat these modalities not as optional add-ons but as first-class citizens, with explicit mechanisms for handling missing channels and quantifying uncertainty when critical evidence (e.g., transporter annotations or ADE profiles) is absent. Meta-learning and pretraining strategies, such as Meta3D-DDI and MGP-DR, illustrate how task-agnostic representations can transfer across datasets and even to related tasks like drug synergy, but their computational cost and potential domain shift must be carefully managed (Lv, 2023; Ren et al., 2022). Figure 10 summarizes the main research directions emerging from the included literature and our synthesis. Figure 11 provides an overview of our classification scheme, core contributions, and the main gaps identified across the included studies.

On the methodological side, contrastive and event-aware objectives are emerging as key tools to disentangle interaction mechanisms and directionality. Methods like MDDI-SCL, MPHGCL-DDI, and SAGAN leverage supervised or hybrid contrastive losses, meta-path semantics, or domain-adversarial training to separate pharmacodynamic/pk mechanisms, tackle cross-domain generalization, and alleviate label imbalance (Qi et al., 2024; Lin et al., 2022; Hu et al., 2024). An immediate research opportunity is to combine these objectives with calibrated probabilistic outputs and uncertainty estimates, so that high-confidence predictions can be routed to automated workflows while ambiguous pairs trigger human review.

For text mining and knowledge-graph construction, our synthesis suggests three priorities. First, open-domain LLMs and retrieval-augmented pipelines, such as DDI-JUDGE and Proto-DDI frameworks, should be coupled with structured evidence schemas to ensure that extracted interactions are traceable and auditable (Qi et al., 2025; Mouazer et al., 2025). Second, pharmaco-safety KGs (e.g., PV-focused KGs and patient-centered backbones like myAURA) show how DDIs can be integrated with diseases, procedures, and social determinants to support holistic decision support (Scientific Reports, 2024; Hauben and Rafi, 2024; Correia et al., 2025). Third, linking these KGs to mechanistic resources on CYP enzymes, transporters, and DILI risk can help transform black-box predictions into clinically actionable explanations (Olubamiwa et al., 2025; Grandits and Ecker, 2024; Wang et al., 2022).

From a clinical and regulatory perspective, only a minority of models are currently embedded into treatment-recommendation systems or evaluated against EHR-derived outcomes. Systems such as KGDNet, MathEagle, and ICU/EHR safety analyses demonstrate that DDI-aware modeling can reduce predicted interaction rates, re-stratify risk levels, and surface novel signals, yet they are mostly limited to single-center or retrospective settings (Zhu Wenjun et al., 2023; Scientific Reports, 2024; Hou et al., 2024). Future work should prioritize multi-site validation, prospective pilot studies, and human–AI collaboration designs that allow clinicians and pharmacovigilance experts to override, annotate, and feed back into models. Finally, our RoB-ML tables highlight a simple but powerful agenda: mandatory reporting of data splits, negative-sampling protocols, and code, along with at least one external validation dataset, should become baseline expectations for DDI modeling papers.

## 8 Conclusion

This systematic review mapped the landscape of computational drug–drug interaction (DDI) research published between 2022 and 2025, spanning DDI prediction, interaction extraction from text, and safety-signal detection across molecular, graph/knowledge-based, textual, and real-world evidence modalities. By structuring the literature through a feature–method taxonomy and examining task formulations, data sources, and evaluation protocols, we find that recent progress is largely driven by richer representations (e.g., knowledge graphs and multimodal fusion) and more expressive learning paradigms (e.g., graph neural networks, contrastive learning, and transformer-based models). However, the field remains constrained by recurring limitations that hinder clinical translation: reliance on a small set of benchmark datasets, inconsistent and sometimes unrealistic validation designs, limited mechanism- and direction-aware modeling, and scarce prospective or external validation on real-world cohorts.

We therefore outline a practical agenda for the next-generation of DDI research: (i) adopt standardized benchmarks with explicit cold-start and out-of-distribution protocols; (ii) develop event direction, and mechanism-aware models that integrate structured pharmacology (e.g., enzymes/transporters, pathways) with multimodal evidence; (iii) evaluate models using calibration and uncertainty reporting in addition to discrimination metrics; (iv) strengthen reproducibility through open code, documented preprocessing, and transparent negative sampling; and (v) validate clinically through prospective studies and integration with EHR and pharmacovigilance pipelines. Advancing along these axes can move computational DDI modeling from retrospective leaderboard performance toward trustworthy decision-support tools that improve medication safety in real-world polypharmacy.

## Data Availability

The original contributions presented in the study are included in the article/Supplementary Material, further inquiries can be directed to the corresponding authors.
