# Supplementary material for: Computational approaches for drug–drug interaction prediction: a systematic review of data sources, modeling strategies, and evaluation frameworks
Source: Front Pharmacol. 2026 Apr 29;17:1816394. doi: 10.3389/fphar.2026.1816394 (PMC13168182; doi:10.3389/fphar.2026.1816394)
Supplement: Supplementary file 1 [file DataSheet1.pdf]

Supplementary Material

1 SUPPLEMENTARY DATA

Table S1: Unique public code repositories/links reported by the included studies.

Ref(s)	Repository/Link
[1] (2023)	Code Link
[2] (2023)	Code Link
[3] (2022)	Code Link
[4] (2024)	Code Link
[5] (2025)	Code Link
[6] (2025)	Code Link
[7] (2024);	Code Link
[8] (2022)	Code Link
[9] (2025)	Code Link
[10] (2023)	Code Link
[11] (2023)	Code Link
[12] (2025)	Code Link
[13] (2025)	Code Link
[14] (2024)	Code Link
[15] (2022)	Code Link
[16] (2022)	Code Link
[17] (2025)	Code Link
[18] (2023)	Code Link
[19] (2023)	Code Link
[20] (2022)	Code Link
[21] (2024)	Code Link
[22] (2025)	Code Link
[23] (2022)	Code Link
[24] (2024)	Code Link
[25] (2024)	Code Link
[25] (2024)	Code Link
[26] (2022)	Code Link
[27] (2024)	Code Link
[28] (2025)	Code Link
[29] (2024)	Code Link
[30] (2024)	Code Link
[31] (2023)	Code Link
[32] (2022)	Code Link

Continued on next page

[33] (2024)	Code Link
[34] (2022)	Code Link
[35] (2023)	Code Link
[36] (2025)	Code Link
[37] (2024)	Code Link
[38] (2025)	Code Link
[39] (2024)	Code Link
[40] (2025)	Code Link
[41] (2025)	Code Link
[42] (2024)	Code Link
[43] (2025)	Code Link
[44] (2025)	Code Link
[45] (2025)	Code Link
[46] (2023)	Code Link
[47] (2025)	Code Link

2 ROB-ML STUDY-LEVEL ASSESSMENT

Table S2: Supplementary Table S2. Study-level RoB-ML assessment for the included studies summarised in the manuscript tables.

Ref	Year	Method group	Bias risk	Data-source quality	Reproducibility	Score
[48]	2025	Transformer / LLM	Moderate concern	Moderate concern	Moderate concern	3
[49]	2025	Text mining / IE / corpus	Moderate concern	Low concern	Moderate concern	4
[50]	2023	Multimodal fusion	Moderate concern	Moderate concern	Moderate concern	3
[1]	2023	Heterogeneous network / meta-path	Moderate concern	Moderate concern	Low concern	4
[51]	2022	Text mining / IE / corpus	Moderate concern	Low concern	Moderate concern	4
[2]	2023	Text mining / IE / corpus	Moderate concern	Low concern	Low concern	5
[52]	2024	Graph neural network	Moderate concern	Low concern	Moderate concern	4
[53]	2024	Recommender systems	Moderate concern	Low concern	Moderate concern	4
[54]	2024	Pharmacovigilance signal detection	Moderate concern	Low concern	Moderate concern	4
[55]	2024	Knowledge graph / embedding	Moderate concern	Moderate concern	High concern	2
[56]	2024	Graph neural network	Moderate concern	Low concern	Moderate concern	4
[3]	2022	Deep learning (non-graph)	Moderate concern	Low concern	Low concern	5
[4]	2024	Contrastive / metric / meta-learning	Moderate concern	Low concern	Low concern	5
[5]	2025	Contrastive / metric / meta-learning	Moderate concern	Low concern	Low concern	5
[57]	2024	Review / Survey	Moderate concern	Moderate concern	Moderate concern	3
[58]	2024	Review / Survey	Moderate concern	Low concern	Moderate concern	4
[59]	2023	Deep learning (non-graph)	Moderate concern	Moderate concern	Moderate concern	3
[60]	2022	Review / Survey	Moderate concern	Moderate concern	Moderate concern	3
[61]	2022	Cheminformatics / QSAR	Moderate concern	Moderate concern	Moderate concern	3
[6]	2025	Multimodal fusion	Moderate concern	High concern	Low concern	3
[7]	2024	Deep learning (non-graph)	Moderate concern	Moderate concern	Low concern	4
[62]	2023	Deep learning (non-graph)	Moderate concern	Low concern	Moderate concern	4
[63]	2023	Multimodal fusion	Moderate concern	High concern	High concern	1
[64]	2023	Text mining / IE / corpus	Moderate concern	Low concern	Moderate concern	4
[8]	2022	Contrastive / metric / meta-learning	Moderate concern	High concern	Low concern	3
[65]	2024	Multimodal fusion	Moderate concern	Moderate concern	Moderate concern	3

Continued on next page

Ref	Year	Method group	Bias risk	Data-source quality	Reproducibility	Score
[9]	2025	Multimodal fusion	Moderate concern	High concern	Low concern	3
[66]	2024	Graph neural network	Moderate concern	High concern	High concern	1
[67]	2022	Knowledge graph / embedding	Moderate concern	Moderate concern	Moderate concern	3
[68]	2022	Representation learning / emb-models	Moderate concern	Low concern	Moderate concern	4
[10]	2023	Multimodal fusion	Moderate concern	Low concern	Low concern	5
[11]	2023	Deep learning (non-graph)	High concern	Moderate concern	Low concern	3
[12]	2025	Multimodal fusion	Moderate concern	High concern	Low concern	3
[69]	2023	Multimodal fusion	Moderate concern	Moderate concern	Moderate concern	3
[70]	2024	Contrastive / metric / meta-learning	Moderate concern	Low concern	Moderate concern	4
[71]	2024	Contrastive / metric / meta-learning	Moderate concern	Low concern	Moderate concern	4
[72]	2023	Representation learning / emb-models	Moderate concern	Moderate concern	Moderate concern	3
[73]	2023	Multimodal fusion	Moderate concern	Moderate concern	Moderate concern	3
[74]	2023	Text mining / IE / corpus	Moderate concern	High concern	High concern	1
[13]	2025	Text mining / IE / corpus	Moderate concern	Low concern	Low concern	5
[14]	2023	Multimodal fusion	Moderate concern	Low concern	Low concern	5
[75]	2023	Heterogeneous network / meta-path	Moderate concern	Moderate concern	Moderate concern	3
[76]	2024	Multimodal fusion	Moderate concern	High concern	High concern	1
[15]	2022	Multimodal fusion	Moderate concern	Moderate concern	Low concern	4
[16]	2022	Multimodal fusion	Moderate concern	High concern	Low concern	3
[17]	2025	Knowledge graph / embedding	Moderate concern	Moderate concern	Low concern	4
[77]	2025	Cheminformatics / QSAR	Moderate concern	Low concern	Moderate concern	4
[78]	2022	Review / Survey	Moderate concern	High concern	High concern	1
[18]	2023	Statistical / regression	High concern	Moderate concern	Low concern	3
[79]	2023	Multimodal fusion	Moderate concern	Low concern	Moderate concern	4
[80]	N/A	Review / Survey	Moderate concern	High concern	High concern	1
[19]	2023	Text mining / IE / corpus	Moderate concern	Low concern	Low concern	5
[81]	2023	Review / Survey	Moderate concern	High concern	High concern	1
[82]	2023	Review / Survey	Moderate concern	High concern	High concern	1
[83]	2024	Deep learning (non-graph)	Moderate concern	Low concern	Moderate concern	4
[84]	2024	Multimodal fusion	Moderate concern	High concern	High concern	1
[85]	2022	Text mining / IE / corpus	Moderate concern	Moderate concern	Moderate concern	3
[20]	2022	Graph neural network	Moderate concern	High concern	Low concern	3
[86]	2024	Multimodal fusion	Moderate concern	High concern	High concern	1
[87]	2024	Deep learning (non-graph)	Moderate concern	Low concern	Moderate concern	4
[88]	2024	Graph neural network	Moderate concern	Moderate concern	Moderate concern	3
[89]	2023	Knowledge graph / embedding	Moderate concern	Low concern	Moderate concern	4
[90]	2024	Clinical study / trial / cohort	High concern	Moderate concern	High concern	1
[91]	2024	Pharmacovigilance signal detection	Moderate concern	Low concern	Moderate concern	4
[92]	2024	Review / Survey	Moderate concern	High concern	High concern	1
[21]	2024	Recommender systems	Moderate concern	Low concern	Low concern	5
[93]	2023	Network-based (non-GNN)	Moderate concern	Moderate concern	Moderate concern	3
[94]	2023	Review / Survey	Moderate concern	Moderate concern	Moderate concern	3
[95]	2024	Clinical study / trial / cohort	High concern	Moderate concern	High concern	1
[96]	2024	Multimodal fusion	Moderate concern	High concern	High concern	1
[97]	2023	Deep learning (non-graph)	Moderate concern	Moderate concern	Moderate concern	3
[98]	2025	Deep learning (non-graph)	Moderate concern	Low concern	Moderate concern	4
[99]	2025	Statistical / regression	Moderate concern	Moderate concern	Moderate concern	3
[100]	2025	Transformer / LLM	Moderate concern	Low concern	Moderate concern	4
[101]	2022	Resource / benchmark / comparison	Moderate concern	Low concern	Moderate concern	4

Continued on next page

Ref	Year	Method group	Bias risk	Data-source quality	Reproducibility	Score
[22]	2025	Contrastive / metric / meta-learning	Moderate concern	High concern	Low concern	3
[102]	2022	Multimodal fusion	Moderate concern	Low concern	Moderate concern	4
[103]	2022	Multimodal fusion	Moderate concern	High concern	High concern	1
[104]	2023	Text mining / IE / corpus	Moderate concern	Low concern	Moderate concern	4
[105]	2024	Review / Survey	Moderate concern	High concern	High concern	1
[106]	2023	Knowledge graph / embedding	Moderate concern	High concern	High concern	1
[107]	2023	Bioinformatics / omics analysis	Moderate concern	Low concern	Moderate concern	4
[108]	2022	Bioinformatics / omics analysis	Moderate concern	Low concern	Moderate concern	4
[109]	2024	Multimodal fusion	Moderate concern	Moderate concern	Moderate concern	3
[23]	2022	Matrix factorization / tensor factorization	Moderate concern	Moderate concern	Low concern	4
[24]	2024	Text mining / IE / corpus	Moderate concern	Low concern	Low concern	5
[110]	2023	Statistical / regression	High concern	Moderate concern	High concern	1
[111]	2024	Computational modeling (general)	Moderate concern	Moderate concern	Moderate concern	3
[112]	2025	Transformer / LLM	Moderate concern	Moderate concern	Moderate concern	3
[113]	2022	Knowledge graph / embedding	Moderate concern	Moderate concern	Moderate concern	3
[114]	2022	Review / Survey	Moderate concern	Low concern	Moderate concern	4
[115]	2022	Review / Survey	Moderate concern	Moderate concern	Moderate concern	3
[116]	2022	Recommender systems	Moderate concern	Low concern	Moderate concern	4
[117]	2024	Multimodal fusion	Moderate concern	Low concern	Moderate concern	4
[118]	2024	Network-based (non-GNN)	Moderate concern	High concern	High concern	1
[119]	2024	Graph neural network	Moderate concern	Low concern	Moderate concern	4
[120]	2024	Text mining / IE / corpus	Moderate concern	Moderate concern	Moderate concern	3
[121]	2023	Classical machine learning	Moderate concern	Moderate concern	Moderate concern	3
[122]	2023	Deep learning (non-graph)	Moderate concern	Low concern	Moderate concern	4
[123]	2022	Multimodal fusion	Moderate concern	High concern	High concern	1
[124]	2024	Representation learning / emb-models	Moderate concern	Moderate concern	Moderate concern	3
[125]	2025	Text mining / IE / corpus	Moderate concern	Low concern	Moderate concern	4
[26]	2022	Text mining / IE / corpus	Moderate concern	Moderate concern	Low concern	4
[126]	2024	Deep learning (non-graph)	Moderate concern	Low concern	Moderate concern	4
[127]	2025	Text mining / IE / corpus	Moderate concern	Moderate concern	Moderate concern	3
[128]	2022	Graph neural network	High concern	High concern	High concern	0
[129]	2022	Classical machine learning	Moderate concern	Moderate concern	Moderate concern	3
[130]	2024	Representation learning / emb-models	Moderate concern	Moderate concern	Moderate concern	3
[131]	2025	Graph neural network	Moderate concern	Moderate concern	Moderate concern	3
[132]	2025	Text mining / IE / corpus	Moderate concern	Low concern	Moderate concern	4
[133]	2023	Deep learning (non-graph)	Moderate concern	Low concern	Moderate concern	4
[134]	2023	Deep learning (non-graph)	Moderate concern	Moderate concern	Moderate concern	3
[135]	2024	Heterogeneous network / meta-path	Moderate concern	Low concern	Moderate concern	4
[136]	2024	Contrastive / metric / meta-learning	Moderate concern	Low concern	Moderate concern	4
[137]	2023	Deep learning (non-graph)	High concern	High concern	High concern	0
[138]	2022	Text mining / IE / corpus	Moderate concern	Low concern	Moderate concern	4
[139]	2024	Reinforcement learning	High concern	High concern	High concern	0
[140]	2024	Recommender systems	Moderate concern	Moderate concern	Moderate concern	3
[27]	2024	Contrastive / metric / meta-learning	Moderate concern	Moderate concern	Low concern	4
[141]	2023	Matrix factorization / tensor factorization	Moderate concern	Low concern	Moderate concern	4
[28]	2025	Contrastive / metric / meta-learning	High concern	High concern	Low concern	2
[142]	2025	Pharmacovigilance signal detection	Moderate concern	Low concern	Moderate concern	4
[29]	2024	Contrastive / metric / meta-learning	Moderate concern	Moderate concern	Low concern	4

Continued on next page

Ref	Year	Method group	Bias risk	Data-source quality	Reproducibility	Score
[143]	2024	Graph neural network	Moderate concern	Moderate concern	Moderate concern	3
[144]	2024	Multimodal fusion	Moderate concern	Moderate concern	Moderate concern	3
[145]	2024	Multimodal fusion	Moderate concern	Moderate concern	Moderate concern	3
[146]	2022	Classical machine learning	Moderate concern	Low concern	Moderate concern	4
[147]	2022	Deep learning (non-graph)	Moderate concern	Moderate concern	Moderate concern	3
[148]	2024	Transformer / LLM	Moderate concern	Moderate concern	Moderate concern	3
[149]	2023	Representation learning / emb-models	Moderate concern	Moderate concern	Moderate concern	3
[30]	2024	Contrastive / metric / meta-learning	Moderate concern	Moderate concern	Low concern	4
[150]	2023	Text mining / IE / corpus	Moderate concern	High concern	High concern	1
[31]	2023	Knowledge graph / embedding	Moderate concern	Moderate concern	Low concern	4
[151]	2025	Graph neural network	Moderate concern	Moderate concern	Moderate concern	3
[152]	2024	Graph neural network	Moderate concern	Moderate concern	Moderate concern	3
[153]	2023	Text mining / IE / corpus	High concern	Moderate concern	High concern	1
[32]	2022	Software tool / library	Moderate concern	Moderate concern	Low concern	4
[154]	2024	Heterogeneous network / meta-path	Moderate concern	Moderate concern	Moderate concern	3
[155]	2024	Graph neural network	Moderate concern	Moderate concern	Moderate concern	3
[156]	2024	Text mining / IE / corpus	Moderate concern	Low concern	Moderate concern	4
[33]	2024	Multimodal fusion	Moderate concern	Moderate concern	Low concern	4
[157]	2025	Transformer / LLM	Moderate concern	High concern	High concern	1
[158]	2023	Multimodal fusion	Moderate concern	Moderate concern	Moderate concern	3
[159]	2024	Knowledge graph / embedding	Moderate concern	Moderate concern	Moderate concern	3
[34]	2022	Text mining / IE / corpus	Moderate concern	Low concern	Low concern	5
[160]	2024	Review / Survey	Moderate concern	High concern	High concern	1
[161]	2023	Bioinformatics / omics analysis	Moderate concern	Moderate concern	Moderate concern	3
[162]	2025	Multimodal fusion	Moderate concern	Low concern	Moderate concern	4
[35]	2023	Multimodal fusion	Moderate concern	Moderate concern	Low concern	4
[163]	2024	Multimodal fusion	Moderate concern	Moderate concern	Moderate concern	3
[164]	2025	Review / Survey	Moderate concern	Moderate concern	Moderate concern	3
[36]	2025	Knowledge graph / embedding	Moderate concern	Moderate concern	Low concern	4
[165]	2022	Representation learning / emb-models	High concern	High concern	High concern	0
[166]	2023	Representation learning / emb-models	Moderate concern	Moderate concern	Moderate concern	3
[167]	2023	Multimodal fusion	Moderate concern	Moderate concern	Moderate concern	3
[168]	2023	Deep learning (non-graph)	Moderate concern	Low concern	Moderate concern	4
[169]	2023	Text mining / IE / corpus	Moderate concern	Moderate concern	Moderate concern	3
[170]	2023	Heterogeneous network / meta-path	Moderate concern	Moderate concern	Moderate concern	3
[171]	2023	Contrastive / metric / meta-learning	Moderate concern	Moderate concern	Moderate concern	3
[172]	2024	Contrastive / metric / meta-learning	Moderate concern	Moderate concern	Moderate concern	3
[173]	2023	Multimodal fusion	Moderate concern	Moderate concern	Moderate concern	3
[174]	2024	Recommender systems	Moderate concern	Moderate concern	Moderate concern	3
[175]	2023	Reinforcement learning	Moderate concern	Moderate concern	Moderate concern	3
[176]	2023	Classical machine learning	Moderate concern	Moderate concern	Moderate concern	3
[177]	2025	Multimodal fusion	Moderate concern	Moderate concern	Moderate concern	3
[178]	2025	Multimodal fusion	Moderate concern	Moderate concern	Moderate concern	3
[179]	2023	Contrastive / metric / meta-learning	Moderate concern	Moderate concern	Moderate concern	3
[180]	2023	Representation learning / emb-models	Moderate concern	Low concern	Moderate concern	4
[181]	2025	Review / Survey	Moderate concern	High concern	High concern	1
[37]	2024	Graph neural network	Moderate concern	Low concern	Low concern	5
[38]	2025	Graph neural network	Moderate concern	Moderate concern	Low concern	4

Continued on next page

Ref	Year	Method group	Bias risk	Data-source quality	Reproducibility	Score
[39]	2024	Deep learning (non-graph)	Moderate concern	Low concern	Low concern	5
[182]	2025	Multimodal fusion	Moderate concern	Low concern	Moderate concern	4
[183]	2025	Multimodal fusion	Moderate concern	Low concern	Moderate concern	4
[40]	2025	Transformer / LLM	Moderate concern	Low concern	Low concern	5
[41]	2025	Text mining / IE / corpus	Moderate concern	Low concern	Low concern	5
[184]	2024	Review / Survey	Moderate concern	High concern	High concern	1
[185]	2024	Multimodal fusion	Moderate concern	Low concern	Moderate concern	4
[186]	2024	Graph neural network	High concern	High concern	High concern	0
[187]	2025	Review / Survey	Moderate concern	Moderate concern	Moderate concern	3
[188]	2025	Graph neural network	Moderate concern	High concern	High concern	1
[189]	2025	Deep learning (non-graph)	Moderate concern	Low concern	Moderate concern	4
[190]	2025	Deep learning (non-graph)	Moderate concern	Moderate concern	Moderate concern	3
[191]	2024	Knowledge graph / embedding	Moderate concern	Moderate concern	Moderate concern	3
[192]	2023	Graph neural network	Moderate concern	Low concern	Moderate concern	4
[193]	2023	Deep learning (non-graph)	Moderate concern	Moderate concern	Moderate concern	3
[194]	2024	Clinical study / trial / cohort	High concern	Moderate concern	High concern	1
[42]	2024	Matrix factorization / tensor factorization	Moderate concern	Moderate concern	Low concern	4
[195]	2023	Multimodal fusion	Moderate concern	Low concern	Moderate concern	4
[196]	2023	Multimodal fusion	Moderate concern	Low concern	Moderate concern	4
[197]	2022	Clinical study / trial / cohort	Moderate concern	Low concern	Moderate concern	4
[198]	2024	Review / Survey	Moderate concern	High concern	High concern	1
[199]	2024	Transformer / LLM	Moderate concern	Low concern	Moderate concern	4
[200]	2022	Knowledge graph / embedding	Moderate concern	Low concern	Moderate concern	4
[201]	2025	Deep learning (non-graph)	Moderate concern	Moderate concern	High concern	2
[202]	2024	Pharmacovigilance signal detection	Moderate concern	Low concern	Moderate concern	4
[203]	2023	Text mining / IE / corpus	Moderate concern	Low concern	Moderate concern	4
[204]	2025	Text mining / IE / corpus	Moderate concern	Moderate concern	Moderate concern	3
[205]	2024	Review / Survey	Moderate concern	Low concern	Moderate concern	4
[206]	2024	Graph neural network	Moderate concern	Low concern	Moderate concern	4
[43]	2025	Multimodal fusion	Moderate concern	High concern	Low concern	3
[207]	2024	Text mining / IE / corpus	High concern	Moderate concern	High concern	1
[44]	2025	Transformer / LLM	Moderate concern	Moderate concern	Low concern	4
[208]	2025	Graph neural network	Moderate concern	Low concern	Moderate concern	4
[209]	2025	Multimodal fusion	Moderate concern	Moderate concern	Moderate concern	3
[210]	2025	Representation learning / emb-models	Moderate concern	High concern	High concern	1
[45]	2025	Transformer / LLM	Moderate concern	Moderate concern	Low concern	4
[46]	2023	Text mining / IE / corpus	Moderate concern	Low concern	Low concern	5
[211]	2024	Multimodal fusion	Moderate concern	Low concern	Moderate concern	4
[212]	2024	Knowledge graph / embedding	Moderate concern	Moderate concern	Moderate concern	3
[213]	2024	Text mining / IE / corpus	Moderate concern	Moderate concern	Moderate concern	3
[214]	2025	Clinical study / trial / cohort	High concern	Moderate concern	High concern	1
[215]	2024	Representation learning / emb-models	High concern	Moderate concern	High concern	1
[47]	2025	Multimodal fusion	Moderate concern	Low concern	Low concern	5
[216]	2024	Knowledge graph / embedding	Moderate concern	Moderate concern	Moderate concern	3
[217]	2025	Contrastive / metric / meta-learning	Moderate concern	Moderate concern	Moderate concern	3
[218]	2025	Review / Survey	Moderate concern	High concern	High concern	1
[219]	2022	Classical machine learning	High concern	Moderate concern	High concern	1
[220]	2022	Statistical / regression	Moderate concern	Low concern	Moderate concern	4
[25]	2024	Transformer / LLM	Moderate concern	Moderate concern	Low concern	4

Continued on next page

Ref	Year	Method group	Bias risk	Data-source quality	Reproducibility	Score
[221]	2025	Text mining / IE / corpus	Moderate concern	Moderate concern	Moderate concern	3
[222]	2025	Deep learning (non-graph)	Moderate concern	Low concern	Moderate concern	4
[223]	2025	Deep learning (non-graph)	Moderate concern	Low concern	Moderate concern	4
[224]	2022	Multimodal fusion	High concern	High concern	High concern	0

REFERENCES

[1] Q. Yue et al. Improving therapeutic synergy score predictions with adverse effects using multi-task heterogeneous network learning. *Briefings in Bioinformatics*, 2023. Code: `https://github.com/Arantir123/HNEMA`.

[2] M. Asada et al. Integrating heterogeneous knowledge graphs into drug–drug interaction extraction from the literature. *Bioinformatics*, 2023. Code: `https://github.com/ZJUNLP/HKG-DDIE`.

[3] Yue Hong, Pengyu Luo, Shuting Jin, and Xiangrong Liu. Lagat: link-aware graph attention network for drug–drug interaction prediction. *Bioinformatics*, 38(24):5406–5412, 2022.

[4] Rogia Kpanou and others. Learning self-supervised molecular representations for drug–drug interaction prediction. *BMC Bioinformatics*, 2024.

[5] Mukun Chen, Jia Wu, Shirui Pan, Fu Lin, Bo Du, Xiuwen Gong, and Wenbin Hu. Knowledge-aware contrastive heterogeneous molecular graph learning. *PLOS Computational Biology*, 21(5):e1013008, 2025.

[6] Guishen Wang, Honghan Chen, Handan Wang, Hairong Gao, Xiaowen Hu, and Chen Cao. Mmddi-sse: A novel multi-modal feature fusion model with static subgraph embedding for drug-drug interaction event prediction. *IEEE Journal of Biomedical and Health Informatics*, 2025.

[7] Lin-Xuan Hou, Hai-Cheng Yi, Zhu-Hong You, Shi-Hong Chen, Jia Zheng, and Chee Keong Kwoh. Matheagle: Accurate prediction of drug-drug interaction events via multi-head attention and heterogeneous attribute graph learning. *Computers in Biology and Medicine*, 177:108642, 2024.

[8] Shenggeng Lin, Weizhi Chen, Gengwang Chen, Songchi Zhou, Dong-Qing Wei, and Yi Xiong. Mddi-scl: predicting multi-type drug-drug interactions via supervised contrastive learning. *Journal of Cheminformatics*, 14:81, 2022.

[9] Lingfeng Wang, Yinghong Li, Yaozheng Zhou, Liping Guo, and Congzhou Chen. Mfe-ddi: A multi-view feature encoding framework for drug–drug interaction prediction. *Computational and Structural Biotechnology Journal*, 27:2473–2480, 2025.

[10] Jun Feng, Mengshi Yu, Yuchen Chen, Jing Zeng, Xu Chen, Shenxin Guo, and Xiongwen Quan. Mm-gann-ddi: Multimodal graph-agnostic neural networks for predicting drug–drug interaction events. *Computers in Biology and Medicine*, 166:107492, 2023.

[11] Qusay Mouazer, Ayad Malek, and Philip Lewis. Model and strategy for predicting and discovering drug–drug interactions. In *Studies in Health Technology and Informatics*, 2023. Prototype pipeline and structured output schema.

[12] J. Xia and collaborators. Mopddi: Predicting drug–drug interaction events based on multimodal mutual orthogonal projection and intermodal consistency loss. *IEEE Journal of Biomedical and Health Informatics*, 29(3), 2025. Early access 2023; issue 2025.

[13] Zhu Yuan, Shuailiang Zhang, Huiyun Zhang, Ping Xie, and Yaxun Jia. Optimized drug-drug interaction extraction with biogpt and focal loss-based attention. *IEEE Journal of Biomedical and Health Informatics*, 2025.

[14]Xiangzhen Shen, Zimeng Li, Yuansheng Liu, Bosheng Song, and Xiangxiang Zeng. Peb-ddi: a task-specific dual-view substructural learning framework for drug–drug interaction prediction. *IEEE Journal of Biomedical and Health Informatics*, 28(1):569–579, 2023.

[15]Li-Ping Kang, Kai-Biao Lin, Ping Lu, Fan Yang, and Jin-Po Chen. Multitype drug interaction prediction based on the deep fusion of drug features and topological relationships. *PLOS ONE*, 17(8):e0273764, 2022. Code: `https://github.com/kk12321/DM-DDI`.

[16]Changxiang He, Yuru Liu, Hao Li, Hui Zhang, Yaping Mao, Xiaofei Qin, Lele Liu, and Xuedian Zhang. Multi-type feature fusion based on graph neural network for drug-drug interaction prediction. *BMC bioinformatics*, 23(1):224, 2022.

[17]Rion Brattig Correia, Jordan C Rozum, Leonard Cross, Jack Felag, Michael Gallant, Ziqi Guo, Bruce W Herr, Aehong Min, Jon Sanchez-Valle, Deborah Stungis Rocha, et al. myaura: a personalized health library for epilepsy management via knowledge graph sparsification and visualization. *Journal of the American Medical Informatics Association*, 33(1):167–181, 2025.

[18]Samantha H Rutherford, Christopher DM Hutchison, Gregory M Greetham, Anthony W Parker, Alison Nordon, Matthew J Baker, and Neil T Hunt. Optical screening and classification of drug binding to proteins in human blood serum. *Analytical Chemistry*, 95(46):17037–17045, 2023.

[19]Mingqing Huang, Zhenchao Jiang, and Shun Guo. Phar-lstm: a pharmacological representation-based lstm network for drug–drug interaction extraction. *PeerJ*, 11:e16606, 2023.

[20]Yue-Hua Feng and Shao-Wu Zhang. Prediction of drug-drug interaction using an attention-based graph neural network on drug molecular graphs. *Molecules*, 27(9):3004, 2022.

[21]Mariam Zomorodi and others. Recomed: A comprehensive pharmaceutical recommendation system. *Artificial Intelligence in Medicine*, 157:102981, 2024.

[22]Chae Eun Lee, Jin Sob Kim, Jin Hong Min, and Sung Won Han. Simson: simple contrastive learning of smiles for molecular property prediction. *Bioinformatics*, 41(5):btaf275, 2025.

[23]Hui Yu, ShiYu Zhao, and JianYu Shi. Stnn-ddi: a substructure-aware tensor neural network to predict drug–drug interactions. *Briefings in Bioinformatics*, 23(4):bbac209, 2022.

[24]Yiyang Shi, Mingxiu He, Junheng Chen, Fangfang Han, and Yongming Cai. Subge-ddi: A new prediction model for drug–drug interaction established through biomedical texts and drug-pairs knowledge subgraph enhancement. *PLOS Computational Biology*, 20(4):e1011989, 2024.

[25]Junkai Cheng, Yijia Zhang, Hengyi Zhang, Shaoxiong Ji, and Mingyu Lu. Transfol: A logical query model for complex relational reasoning in drug-drug interaction. *IEEE Journal of Biomedical and Health Informatics*, 28(8):4975–4985, 2024.

[26]Shijun Zhang and others. Translational drug–interaction corpus. *Database*, page baac031, 2022.

[27]Zhenyu Jiang and others. Geometric molecular graph representation learning model for drug-drug interactions prediction. *IEEE Journal of Biomedical and Health Informatics*, 2024.

[28]Shuai Zhang and others. Iib-ddi: Invariant information bottle theory for out-of-distribution drug-drug interaction prediction. *IEEE Transactions on Computational Biology and Bioinformatics*, 2025.

[29]Tengfei Ma and others. Learning to denoise biomedical knowledge graph for robust molecular interaction prediction. *IEEE Transactions on Knowledge and Data Engineering*, 36(12):8682–8694, 2024.

[30]Jian Zhong, Haochen Zhao, Qichang Zhao, and Jianxin Wang. A knowledge graph-based method for drug-drug interaction prediction with contrastive learning. *IEEE/ACM Transactions on Computational Biology and Bioinformatics*, 21(6):2485–2498, 2024.

[31]Yongqi Zhang and others. Adaprop: Learning adaptive propagation for graph neural network based knowledge graph reasoning. In *Proceedings of the 29th ACM SIGKDD (KDD '23)*, 2023. Code: https://github.com/LARS-research/AdaProp.

[32]Benedek Rozemberczki and others. Chemicalx: A deep learning library for drug pair scoring. In *Proceedings of the 28th ACM SIGKDD (KDD '22)*, 2022. Code: https://github.com/astrazeneca/chemicalx.

[33]Liangwei Nathan Zheng and others. Devil in the tail: A multi-modal framework for drug-drug interaction prediction in long tail distinction. In *Proceedings of the 33rd ACM CIKM (CIKM '24)*, 2024. Code: https://github.com/IcurasLW/TFMD$_{L}ongtailed_{D}DI.$

[34]Xin Jin and others. Extracting drug-drug interactions from biomedical texts using knowledge graph embeddings and multi-focal loss. In *Proceedings of the 31st ACM CIKM (CIKM '22)*, 2022. Code: https://github.com/NWU-IPMI/DDIE-KGE-MFL.

[35]Changzhi Jiang and others. Kgnmda: A knowledge graph neural network method for predicting microbe-disease associations. *IEEE/ACM TCBB 2023*, 2023. Code: https://github.com/ChangzhiJiang/KGNMDA$_{m}aster.$

[36]Tassallah Abdullahi and others. K-paths: Reasoning over graph paths for drug repurposing and drug interaction prediction. In *KDD 2025*, 2025. Code: https://github.com/rsinghlab/K-Paths.

[37]Qi Zhang, Yuxiao Wei, and Liwei Liu. A domain adaptive interpretable substructure-aware graph attention network for drug–drug interaction prediction. *Interdisciplinary Sciences: Computational Life Sciences*, 2024.

[38]Fatemeh Nasiri and Mohsen Hooshmand. A graph attention–based deep learning network for predicting biotech–small-molecule drug interactions. *Bioinformatics Advances*, 2025. Code: https://github.com/BioinformaticsIASBS/BSI-Net.

[39]Jiaxi He, Yuping Sun, and Jie Ling. A molecular fragment representation learning framework for drug–drug interaction prediction. *Interdisciplinary Sciences: Computational Life Sciences*, 17:42–58, 2025.

[40]Zihui Cheng and others. A multi-view feature-based interpretable deep learning framework for drug-drug interaction prediction. *Interdisciplinary Sciences: Computational Life Sciences*, 2025. Code: https://github.com/ZihuiCheng/MI-DDI.

[41]Changqing Yu Zhang, Ting and Shanwen Zhang. Ca-sqbg: Cross-attention guided siamese quantum bigru for drug–drug interaction extraction. *Computers in Biology and Medicine*, 2025. Code: https://github.com/xaycq/CA-SQBG.

[42]Guosheng Han and others. Ctf-ddi: Constrained tensor factorization for drug–drug interactions prediction. *Future Generation Computer Systems*, 2024. Code: https://github.com/angelfacedac/CTF$_{D}DI.$

[43]Hong Wang and others. Eddinet: Enhancing drug–drug interaction prediction via information flow and consensus constrained multi-graph contrastive learning. *Artificial Intelligence in Medicine*, 2025. Code: https://github.com/95LY/EDDINet$_{c}ode.$

[44]Kivanc Bayraktar and others. Enhancing drug-drug interaction classification by leveraging textual drug arguments. *Computers in Biology and Medicine*, 2025. Code: https://github.com/kivancbayraktar/enhancing-ddi-classification-via-textual-arguments.

[45]He Qi and others. Improving drug-drug interaction prediction via in-context learning and judging with large language models. *Frontiers in Pharmacology*, 2025. Code: https://github.com/zcc1203/ddi-judge.

[46]Makoto Miwa Asada, Masaki and Yutaka Sasaki. Integrating heterogeneous knowledge graphs into drug–drug interaction extraction from the literature. *Bioinformatics*, 2023. Code: https://github.com/tticoin/HKG-DDIE.git.

[47]Changpeng Zhao and others. Kgdb-ddi: Knowledge graph-based drug background data fusion model for drug–drug interaction prediction. *Artificial Intelligence in Medicine*, 2025.

[48]X. Qi et al. Improving drug–drug interaction prediction via in-context learning and judging with large language models. *Frontiers in Pharmacology*, 2025.

[49]Seonwoo Jung and Choongkoo Yoo. Interpretable prediction of drug–drug interactions via text embedding in biomedical literature. *Computers in Biology and Medicine*, 185:109496, 2025.

[50]Weizhong Zhao et al. Improving drug–drug interactions prediction with interpretability via meta-path-based information fusion. *Briefings in Bioinformatics*, 2023.

[51]Y. Duan et al. Imse: interaction information attention and molecular structure based drug–drug interaction extraction. *BMC Bioinformatics*, 2022.

[52]Xiaorui Su, Bowei Zhao, Guodong Li, Jun Zhang, Pengwei Hu, Lun Hu, and Zhu-Hong You. Knowledge graph neural network with spatial-aware capsule for drug–drug interaction prediction. *IEEE Journal of Biomedical and Health Informatics*, 2025.

[53]R. M. and S. S. Knowledge graph driven medicine recommendation system using graph neural networks on longitudinal medical records. *Scientific Reports*, 14:25449, 2024.

[54]M. Hauben and M. Rafi. Knowledge graphs in pharmacovigilance: A step-by-step guide. *Clinical Therapeutics*, 46:538–543, 2024.

[55]Wensu Liu, Tianyu Tang, Jianwei Feng, Chunyu Wang, Lin Lin, Shengli Wang, Kai Zeng, Renlong Zou, Zeyu Yang, and Yue Zhao. Knowledge graph construction based on granulosa cells transcriptome from polycystic ovary syndrome with normoandrogen and hyperandrogen. *Journal of Ovarian Research*, 17:38, 2024.

[56]Junpeng Lin and others. Masmddi: multi-layer adaptive soft-mask graph neural network for drug–drug interaction prediction. *Frontiers in Pharmacology*, 2024.

[57]Rufan Yao, Zhenhua Shen, Xinyi Xu, Guixia Ling, Rongwu Xiang, Tingyan Song, Fei Zhai, and Yuxuan Zhai. Knowledge mapping of graph neural networks for drug discovery: a bibliometric and visualized analysis. *Frontiers in Pharmacology*, 15:1393415, 2024.

[58]Melanie Grandits and Gerhard F Ecker. Ligand-and structure-based approaches for transmembrane transporter modeling. *Current Drug Research Reviews Formerly: Current Drug Abuse Reviews*, 16(2):81–93, 2024.

[59]Anna Giczewska and et al Pastuszak. Longitudinal drug synergy assessment using convolutional neural network image-decoding of glioblastoma single-spheroid cultures. 5(1):vdad134, 2023.

[60]Jennifer M Cantrell, Carolina H Chung, and Sriram Chandrasekaran. Machine learning to design antimicrobial combination therapies: Promises and pitfalls. *Drug Discovery Today*, 27(6):1639–1651, 2022.

[61]Ning-Ning Wang, Xiang-Gui Wang, Guo-Li Xiong, Zi-Yi Yang, Ai-Ping Lu, Xiang Chen, Shao Liu, Ting-Jun Hou, and Dong-Sheng Cao. Machine learning to predict metabolic drug interactions related to cytochrome p450 isozymes. *Journal of Cheminformatics*, 14(1):23, 2022.

[62]Shenggeng Lin, Xueying Mao, Liang Hong, Shuangjun Lin, Dong-Qing Wei, and Yi Xiong. Matt-ddi: Predicting multi-type drug-drug interactions via heterogeneous attention mechanisms. *Methods*, 220:1–10, 2023.

[63]Chen-Di Han, Chun-Chun Wang, Li Huang, and Xing Chen. Mcff-mtddi: multi-channel feature fusion for multi-typed drug–drug interaction prediction. *Briefings in Bioinformatics*, 24(4):bbad215, 2023.

[64]Ying Qian, Xinyi Li, Jian Wu, and Qian Zhang. Mcl-dti: using drug multimodal information and bi-directional cross-attention learning method for predicting drug–target interaction. *BMC Bioinformatics*, 24:323, 2023.

[65]Xingyue Gu, Junkai Liu, Yue Yu, Pengfeng Xiao, and Yijie Ding. Mfd–gdrug: multimodal feature fusion-based deep learning for gpcr–drug interaction prediction. *Methods*, 223:75–82, 2024.

[66]Guannan Geng, Lizhuang Wang, Yanwei Xu, Tianshuo Wang, Wei Ma, Hongliang Duan, Jiahui Zhang, and Anqiong Mao. Mgddi: A multi-scale graph neural networks for drug–drug interaction prediction. *Methods*, 228:22–29, 2024.

[67]Yi Zhang, Zhouhan Li, Biao Duan, Lei Qin, and Jing Peng. Mkge: Knowledge graph embedding with molecular structure information. *Computational Biology and Chemistry*, 100:107730, 2022.

[68]Yu Ren, Zhenhua Gao, Zhihui Gao, et al. Multidrug representation learning based on pretraining model and molecular graph for drug interaction and combination prediction. *Bioinformatics*, 38(18):4387–4394, 2022.

[69]J. Sun, Q. Zhao, et al. A multi-pooling feature fusion model for predicting synergistic drug combinations. *Methods*, 217:1–9, 2023.

[70]Baofang Hu, Zhenmei Yu, and Mingke Li. Mphgcl-ddi: meta-path-based heterogeneous graph contrastive learning for drug-drug interaction prediction. *Molecules*, 29(11):2483, 2024.

[71]Yu Li, Lin-Xuan Hou, Zhu-Hong You, Yang Yuan, Cheng-Gang Mi, Yu-an Huang, and Hai-Cheng Yi. Mrgcddi: Multi-relation graph contrastive learning without data augmentation for drug-drug interaction events prediction. *IEEE Journal of Biomedical and Health Informatics*, 2024.

[72]Liyi Yu, Zhaochun Xu, Meiling Cheng, Weizhong Lin, Wangren Qiu, and Xuan Xiao. Mseddi: Multi-scale embedding for predicting drug—drug interaction events. *International Journal of Molecular Sciences*, 24(5), 2023.

[73]Lin Guo, Xiujuan Lei, Ming Chen, and Yi Pan. Msresg: Using gae and residual gcn to predict drug–drug interactions based on multi-source drug features. *Interdisciplinary Sciences: Computational Life Sciences*, 15:171–188, 2023.

[74]Haohan Deng, Qiaoqin Li, Yongguo Liu, and Jiajing Zhu. Mtmg: A multi-task model with multi-granularity information for drug-drug interaction extraction. *Heliyon*, 9(6), 2023.

[75]Xiuhong Li, Hao Yuan, Xiaoliang Wu, Chengyi Wang, Meitao Wu, Hongbo Shi, and Yingli Lv. Multidsmda: Integrating multiple data sources into heterogeneous network for predicting novel metabolite-drug associations. *Computers in Biology and Medicine*, 162:107067, 2023.

[76]Muhammad Asfand-E-Yar, Qadeer Hashir, Asghar Ali Shah, Hafiz Abid Mahmood Malik, Abdullah Alourani, and Waqar Khalil. Multimodal cnn-ddi: using multimodal cnn for drug to drug interaction associated events. *Scientific Reports*, 14(1):4076, 2024.

[77]Sadegh Faramarzi, Arianna Bassan, Kevin P Cross, Xinning Yang, Glenn J Myatt, Donna A Volpe, and Lidiya Stavitskaya. Novel (q) sar models for prediction of reversible and time-dependent inhibition of cytochrome p450 enzymes. *Frontiers in Pharmacology*, 15:1451164, 2025.

[78]Thanh Hoa Vo, Ngan Thi Kim Nguyen, Quang Hien Kha, and Nguyen Quoc Khanh Le. On the road to explainable ai in drug-drug interactions prediction: A systematic review. *Computational and Structural Biotechnology Journal*, 20:2112–2123, 2022.

[79]Jiwon Seo, Hyein Jung, and Younhee Ko. Prid: prediction model using rwr for interactions between drugs. *Pharmaceutics*, 15(10):2469, 2023.

[80]Daniel Amsterdam. Perspective: limiting antimicrobial resistance with artificial intelligence/machine learning. *BME frontiers*, 4:0033, 2023.

[81]Lei Sun, Kun Mi, Yixuan Hou, Tianyi Hui, Lan Zhang, Yanfei Tao, Zhenli Liu, and Lingli Huang. Pharmacokinetic and pharmacodynamic drug–drug interactions: research methods and applications. *Metabolites*, 13(8):897, 2023.

[82]Gianfranco Damiani, Gerardo Altamura, Massimo Zedda, Mario Cesare Nurchis, Giovanni Aulino, Aurora Heidar Alizadeh, Francesca Cazzato, Gabriele Della Morte, Matteo Caputo, Simone Grassi, et al. Potentiality of algorithms and artificial intelligence adoption to improve medication management in primary care: a systematic review. *BMJ open*, 13(3):e065301, 2023.

[83]Xiaoying Yan, Chi Gu, Yuehua Feng, and Jiaxin Han. Predicting drug-drug interaction with graph mutual interaction attention mechanism. *Methods*, 223:16–25, 2024.

[84]Liyuan Zhang, Yongxin Sheng, Jinxiang Yang, Zuhai Hu, and Bin Peng. Predicting the toxic side effects of drug interactions using chemical structures and protein sequences. *Scientific Reports*, 14(1):31503, 2024.

[85]Mohammad Hussain Al-Rabeah and Amir Lakizadeh. Prediction of drug-drug interaction events using graph neural networks based feature extraction. *Scientific Reports*, 12(1):15590, 2022.

[86]Dawei Pan, Ping Lu, Yunbing Wu, Liping Kang, Fengxin Huang, Kaibiao Lin, and Fan Yang. Prediction of multiple types of drug interactions based on multi-scale fusion and dual-view fusion. *Frontiers in Pharmacology*, 15:1354540, 2024.

[87]Jiayue Qiu, Xiao Yan, Yanan Tian, Qin Li, Xiaomeng Liu, Yuwei Yang, Henry HY Tong, and Huanxiang Liu. Ptb-ddi: an accurate and simple framework for drug–drug interaction prediction based on pre-trained tokenizer and bilstm model. *International Journal of Molecular Sciences*, 25(21):11385, 2024.

[88]Zhenxing Wang and Zhongyu Wei. Pt-kgnn: A framework for pre-training biomedical knowledge graphs with graph neural networks. *Computers in Biology and Medicine*, 178:108768, 2024.

[89]Jiacheng Lin, Lijun Wu, Jinhua Zhu, Xiaobo Liang, Yingce Xia, Shufang Xie, Tao Qin, and Tie-Yan Liu. R2-ddi: relation-aware feature refinement for drug–drug interaction prediction. *Briefings in bioinformatics*, 24(1), 2023.

[90]Hines and others. Rationale and design for a pragmatic randomized trial to assess gene-based prescribing for ssris in the treatment of depression. *Clinical and Translational Science*, 17(6):e13822, 2024.

[91]S. Liang, Y. Wang, H. Chen, et al. Real-world safety of levetiracetam: Mining and analysis of its adverse drug reactions based on the faers database. *Frontiers in Pharmacology*, 2024.

[92]Yao and others. Recent development of machine learning models for the prediction of drug–drug interactions. *Frontiers in Pharmacology*, 2024.

[93]Eugene Jeong, Bradley Malin, Scott D Nelson, Yu Su, Lang Li, and You Chen. Revealing the dynamic landscape of drug-drug interactions through network analysis, 2023.

[94]Arun Singh, Shivani B Paruthy, Vivek Belsariya, Sunil Kumar Singh, Sri Saran Manivasagam, Sushila Choudhary, M Anil Kumar, Dhananjay Khera, Vaibhav Kuraria, Shivani B Paruthy Sr, et al. Revolutionizing breast healthcare: harnessing the role of artificial intelligence. *Cureus*, 15(12), 2023.

[95]Rompala and others. Profiling neuronal methylome and hydroxymethylome of opioid use disorder in the human orbitofrontal cortex. *Nature Communications*, 14(1):4544, 2023.

[96]Jing Zhu, Chao Che, Hao Jiang, Jian Xu, Jiajun Yin, and Zhaoqian Zhong. Ssf-ddi: a deep learning method utilizing drug sequence and substructure features for drug–drug interaction prediction. *BMC bioinformatics*, 25(1):39, 2024.

[97]Anna Torkamannia, Yadollah Omidi, and Reza Ferdousi. Syndeep: a deep learning approach for the prediction of cancer drugs synergy. *Scientific Reports*, 13:6184, 2023.

[98]Eman Saad, Sherif Kishk, Amr Ali-Eldin, and Ahmed I. Saleh. Sb-agt: A stochastic beam search-enhanced attention-based gumbel tree framework for drug–drug interaction extraction from biomedical literature. *Computers in Biology and Medicine*, 189:110011, 2025.

[99]Riikka Huusari, Tianduanyi Wang, Sandor Szedmak, Diogo Dias, Tero Aittokallio, and Juho Rousu. Scaling up drug combination surface prediction. *Briefings in Bioinformatics*, 26(2):bbaf099, 2025.

[100]Shanwen Zhang, Changqing Yu, and Chuanlei Zhang. Scatrans: semantic cross-attention transformer for drug–drug interaction predication through multimodal biomedical data. *BMC Bioinformatics*, 26(157), 2025.

[101]Elpida Kontsioti, Simon Maskell, Amina Bensalem, Bhaskar Dutta, and Munir Pirmohamed. Similarity and consistency assessment of three major online drug–drug interaction resources. *British Journal of Clinical Pharmacology*, 88:4067–4079, 2022.

[102]Dingkai Huang, Hongjian He, Jiaming Ouyang, Chang Zhao, Xin Dong, and Jiang Xie. Small molecule drug and biotech drug interaction prediction based on multi-modal representation learning. *BMC Bioinformatics*, 23:561, 2022.

[103]Xueting Han, Ruixia Xie, Xutao Li, and Junyi Li. Smilegnn: Drug–drug interaction prediction based on the smiles and graph neural network. *Life*, 12(319), 2022.

[104]Chenglin Yang, Jiamei Deng, Xianlai Chen, and Ying An. Spbere: Boosting span-based pipeline biomedical entity and relation extraction via entity information. *Journal of Biomedical Informatics*, 145:104456, 2023.

[105]Chiranjib Chakraborty, Manojit Bhattacharya, Sang-Soo Lee, Zhi-Hong Wen, and Yi-Hao Lo. The changing scenario of drug discovery using ai to deep learning: Recent advancement, success stories, collaborations, and challenges. *Molecular Therapy – Nucleic Acids*, 35:102295, 2024.

[106]Mihai Udrescu, Sebastian Mihai Ardelean, and Lucreţia Udrescu. The curse and blessing of abundance—the evolution of drug interaction databases and their impact on drug network analysis. *GigaScience*, 12:1–22, 2023.

[107]Hibah Shaath et al. Therapeutic targeting of the tpx2/ttk network in colorectal cancer. *Cell Communication and Signaling*, 21:265, 2023.

[108]Qinyan Shen, Jiang Wang, and Liangying Zhao. To investigate the internal association between sars-cov-2 infections and cancer through bioinformatics. *Mathematical Biosciences and Engineering*, 19(11):11172–11194, 2022.

[109]Yizhen Luo, Xing Yi Liu, Kai Yang, Kui Huang, Massimo Hong, Jiahuan Zhang, Yushuai Wu, and Zaiqing Nie. Toward unified ai drug discovery with multimodal knowledge. *Health Data Science*, 4:0113, 2024.

[110]Amina Sabir, Muhammad Irfan Majeed, et al. Surface-enhanced raman spectroscopy for studying the interaction of n-propyl substituted imidazole compound with salmon sperm dna. *Photodiagnosis and Photodynamic Therapy*, 41:103262, 2023.

[111]Shi and others. Syn-com: A multi-level predictive synergy framework for innovative drug combinations. *Pharmaceuticals*, 17(9):1230, 2024.

[112]Jianbo Qiao, Xu Guo, Junru Jin, Ding Wang, Kefei Li, Wenjia Gao, Feifei Cui, Zilong Zhang, Hua Shi, and Leyi Wei. Taco-ddi: accurate prediction of drug–drug interaction events using graph transformer-based architecture and dynamic co-attention matrices. *Neural Networks*, 189:107655, 2025.

[113]Xiaorui Su, Bowei Zhao, Zhuhong You, Deshuang Huang, Lei Wang, Leon Wong, and Boya Ji. Biomedical knowledge graph embedding with capsule network for multi-label drug–drug interaction prediction. *IEEE Transactions on Knowledge and Data Engineering*, 35(6):5640–5654, 2023.

[114]Yang Qiu, Yang Zhang, Yifan Deng, Shichao Liu, and Wen Zhang. A comprehensive review of computational methods for drug–drug interaction detection. *IEEE/ACM Transactions on Computational Biology and Bioinformatics*, 19(4):1968–1985, 2022.

[115]Ting Wu et al. A comprehensive survey of graph neural networks for knowledge graphs. *IEEE Access*, 2022.

[116]Yongjian Ren and others. A drug recommendation model based on message propagation and ddi gating mechanism. *IEEE Journal of Biomedical and Health Informatics*, 2022.

[117]Han Zhang Kang, Chuanze and Yanbin Yin. A dual-modality complex-valued fusion method for predicting side effects of drug–drug interactions. *IEEE Journal of Biomedical and Health Informatics*, 2024.

[118]Huan Wang and others. A network enhancement method to identify spurious drug–drug interactions. *IEEE/ACM Transactions on Computational Biology and Bioinformatics*, 2024.

[119]Jianliang Gao and others. Autoddi: Drug–drug interaction prediction with automated graph neural network. *IEEE Journal of Biomedical and Health Informatics*, 2024.

[120]Yaxun Jia and others. Bbl-gat: A novel method for drug–drug interaction extraction from biomedical literature. *IEEE Access*, 2024.

[121]Sidra Abbas and others. A novel drug–drug indicator dataset and ensemble stacking model for detection and classification of drug–drug interaction indicators. *IEEE Access*, 2023.

[122]Weixin Xie and others. Multiple sampling schemes and deep learning improve active learning performance in drug–drug interaction information retrieval analysis from the literature. *Journal of Biomedical Semantics*, 14(5), 2023.

[123]Jiang Xie and others. Tp-ddi: A two-pathway deep neural network for drug–drug interaction prediction. *Interdisciplinary Sciences: Computational Life Sciences*, 14:895–905, 2022.

[124]Carolina H. Chung and others. Transfer learning predicts species-specific drug interactions in emerging pathogens. *bioRxiv*, 2024.

[125]Dimitrios Zaikis and Ioannis Vlahavas. Transformddi: The transformer-based joint multi-task model for end-to-end drug–drug interaction extraction. *IEEE Journal of Biomedical and Health Informatics*, 29(4):3045–3058, 2025.

[126]Michael Hecker, Niklas Frahm, and Uwe K. Zettl. Update and application of a deep learning model for the prediction of interactions between drugs used by patients with multiple sclerosis. *Pharmaceutics*, 16(3), 2024.

[127]Ruoxuan Zhang and others. When east meets west: Cross-domain drug interaction annotations with large language models and bidirectional neural networks. *IEEE Journal of Biomedical and Health Informatics*, 2025.

[128]Ziduo Yang and others. Learning size-adaptive molecular substructures for explainable drug–drug interaction prediction by substructure-aware graph neural network. *Chemical Science*, 13:8693–8703, 2022.

[129]Sven Van Laere and others. Machine learning techniques outperform conventional statistical methods in the prediction of high risk qtc prolongation related to a drug–drug interaction. *Journal of Medical Systems*, 46:100, 2022.

[130]Zhenchao Tang and others. Dsil-ddi: Domain-invariant substructure interaction learning for generalizable drug–drug interaction prediction. *IEEE Transactions on Neural Networks and Learning Systems*, 35(8):10552–10566, 2024.

[131]Lin Guo and others. Dualc: Drug–drug interaction prediction based on dual latent feature extractions. *IEEE Transactions on Emerging Topics in Computational Intelligence*, 9(1):946–958, 2025.

[132]Dandan Song Guo, Xiechao and Fang Yang. Enhanced attention-driven dynamic graph convolutional network for extracting drug–drug interaction. *Big Data Mining and Analytics*, 8(1):257–271, 2025.

[133]Shichao Liu and others. Enhancing drug–drug interaction prediction using deep attention neural networks. *IEEE/ACM Transactions on Computational Biology and Bioinformatics*, 20(2):976–988, 2023.

[134]Junhyun Lee and others. Co-attention graph pooling for efficient pairwise graph interaction learning. *IEEE Access*, 11:78549–78563, 2023.

[135]Farhan Tanvir and others. Ddi prediction with heterogeneous information network – meta-path based approach. *IEEE/ACM Transactions on Computational Biology and Bioinformatics*, 21(5):1168–1182, 2024.

[136]Srinitish Srinivasan ChandraUmakantham, Omkumar and Varenya Pathak. Detecting side effects of adverse drug reactions through drug–drug interactions using graph neural networks and self-supervised learning. *IEEE Access*, 12:93823–93839, 2024.

[137]Hui Yu, KangKang Li, and JianYu Shi. Dganddi: Double generative adversarial networks for drug-drug interaction prediction. *IEEE/ACM Transactions on Computational Biology and Bioinformatics*, 20(3):1854–1867, 2023.

[138]Hongbin Lu, Dingxin Song, Yu Zhu, and Lishuang Li. Drug–drug interaction extraction using drug knowledge graph. In *Proceedings of the IEEE International Conference on Bioinformatics and Biomedicine (BIBM)*, pages 3846–3853, 2022.

[139]Ke Zhao and others. Molecular substructure-aware network with reinforcement pooling and deep attention mechanism for drug-drug interaction prediction. *IEEE Access*, 12:34877–34888, 2024.

[140]Chuang Zhao, Hongke Zhao, Xiaofang Zhou, and Xiaomeng Li. Enhancing precision drug recommendations via in-depth exploration of motif relationships. *IEEE Transactions on Knowledge and Data Engineering*, 2024.

[141]Stuti Jain and others. Graph regularized probabilistic matrix factorization for drug-drug interactions prediction. *IEEE Journal of Biomedical and Health Informatics*, 27(5):2565–2574, 2023.

[142]Ayman Mohamed Mostafa and others. Innovative tailored semantic embedding and machine learning for precise prediction of drug-drug interaction seriousness. *IEEE Access*, 2025.

[143]Xiang Li and others. Mfhg-ddi: An enhanced hybrid graph method leveraging multiple features for predicting drug–drug interactions. *IEEE Access*, 2024.

[144]Guishen Wang and others. Mmddi-mgpff: Multi-modal drug representation learning with molecular graph and pharmacological feature fusion for drug-drug interaction event prediction. In *IEEE BIBM 2024*, 2024.

[145]Mengyuan Jin and others. Multimodal feature fusion-based node representation learning for drug-drug interaction prediction. In *IEEE CEI 2024 (International Conference on Computer Science, Electronic Information Engineering and Intelligent Control Technology)*, 2024.

[146]Guihua Yan C and others. Predicting drug-drug interactions based on integrated similarity and semi-supervised learning (ddi-is-sl). *IEEE/ACM Transactions on Computational Biology and Bioinformatics*, 2022.

[147]Shweta Yadav and others. Relation extraction from biomedical and clinical text: Unified multitask learning framework. *IEEE/ACM Transactions on Computational Biology and Bioinformatics*, 2022.

[148]Yingying Wang, Yun Xiong, Xixi Wu, Xiangguo Sun, Jiawei Zhang, and GuangYong Zheng. Ddiprompt: Drug-drug interaction event prediction based on graph prompt learning. In *Proceedings of the 33rd ACM International Conference on Information and Knowledge Management*, pages 2431–2441, 2024.

[149]Chang Sun, Rong Tang, Jipeng Huang, Jin-mao Wei, and Jian Liu. A deep neural network-based co-coding method to predict drug-protein interactions by analyzing the feature consistency between drugs and proteins. *IEEE/ACM Transactions on Computational Biology and Bioinformatics*, 20(3):2200–2209, 2023.

[150]Qianlong Wen and others. A multi-modality framework for drug-drug interaction prediction by harnessing multi-source data. In *Proceedings of the 32nd ACM CIKM (CIKM '23)*, 2023.

[151]Zhengmao Yang Zhang, Shuai and Sen Jin. Ai-ddi: An attention-based substructure interactive model for predicting drug-drug interaction. In *Proceedings of BIC 2025*, 2025.

[152]Jie Yang and others. An end-to-end knowledge graph fused graph neural network for accurate protein-protein interactions prediction. *IEEE/ACM Transactions on Computational Biology and Bioinformatics*, 2024.

[153]Tushar Agarwal Sinha, Shweta and Pratiyush Pandey. Artificial intelligence in health care: Medical named entity recognition-based audio prescription generator. *ICIMMI 2023*, 2023.

[154]Farhan Tanvir and others. Ddi prediction with heterogeneous information network - meta-path based approach. *IEEE/ACM Transactions on Computational Biology and Bioinformatics*, 2024.

[155]Ran Zhang and others. H2d: Hierarchical heterogeneous graph learning framework for drug-drug interaction prediction. In *Proceedings of the 33rd ACM CIKM (CIKM '24)*, 2024.

[156]M. Shoaib Malik and others. Deepmedfeature: An accurate feature extraction and drug-drug interaction model for clinical text in medical informatics. *ACM Transactions on Asian and Low-Resource Language Information Processing*, 2024.

[157]Yue Leng and Haiming Gu. Drug–drug interaction prediction with interpretable efficient channel attention mechanism and transformer. *IC-BIS 2025*, 2025.

[158]Xiaolong Zhang Ye, Qing and Xiaoli Lin. Drug-target interaction prediction via graph auto-encoder and multi-subspace deep neural networks (gaemsdnn). *IEEE/ACM Transactions on Computational Biology and Bioinformatics*, 2023.

[159]Guodong Peng and Xiangmin Ji. Effective knowledge graph embeddings based on cnn-lstm for drug-drug interactions prediction. In *Proceedings of CFIMA 2024*, 2024.

[160]Mingliang Dou and others. Drug–drug interaction relation extraction based on deep learning: A review. *ACM Computing Surveys*, 2024.

[161]Tie Hua Zhou and others. Predicting drug target genes based on ppi and pdi networks. In *HP3C 2023*, 2023.

[162]Ning Liu and others. Incomplete multi-view drug recommendation via multi-level representation learning and curriculum learning. In *KDD 2025*, 2025.

[163]Xiaoli Lin and others. Kgrlff: Detecting drug-drug interactions based on knowledge graph representation learning and feature fusion. *IEEE/ACM TCBB 2024*, 2024.

[164]Anastasia Barkova Zhong, Zhiqiang and Davide Mottin. Knowledge-augmented graph machine learning for drug discovery: A survey. *ACM Computing Surveys 2025*, 2025.

[165]Yongliang Shen Zhu, Xinyu and Weiming Lu. Molecular substructure-aware network for drug-drug interaction prediction. In *CIKM 2022*, 2022.

[166]Huan Wang and others. Multitype perception method for drug-target interaction prediction. *IEEE/ACM TCBB 2023*, 2023.

[167]Junyu Luo and others. padr: Towards personalized adverse drug reaction prediction by modeling multi-sourced data. In *CIKM 2023*, 2023.

[168]Rupika Wijesinghe Maduka, Rashini and Ruvan Weerasinghe. Predicted drug-drug interactions in diabetes mellitus with graph convolutional autoencoder. In *ICCBB 2023*, 2023.

[169]Weisu Li and others. Visualization of adverse drug reactions based on the knowledge graphs. In *SPML 2023*, 2023.

[170]Farhan Tanvir and others. Predicting drug-drug interactions using heterogeneous graph attention networks. In *ACM-BCB 2023*, 2023.

[171]Jiatao Chen and others. Predicting drug-target interaction via self-supervised learning. *IEEE/ACM TCBB 2023*, 2023.

[172]Nikolaos Astras and Dimitrios Vogiatzis. Prediction of drug-drug interactions with zero-shot learning. *SETN 2024*, 2024.

[173]Sriparna Saha Jha, Kanchan and Sourav Karmakar. Prediction of protein-protein interactions using vision transformer and language model. *IEEE/ACM TCBB 2023*, 2023.

[174]Drishika Chauhan and others. Predictive modeling of drug-drug interactions: A link prediction approach. In *IC3 2024*, 2024.

[175]Xinhang Li and others. Rest: Drug-drug interaction prediction via reinforced student-teacher curriculum learning. In *CIKM 2023*, 2023.

[176]Pedro Ilidio Alves, Andre and Ricardo Cerri. Semi-supervised hybrid predictive bi-clustering trees for drug-target interaction prediction. In *SAC 2023*, 2023.

[177]Ming Zhang Chen, Mengjie and Cunquan Qu. Towards interpretable drug-drug interaction prediction: Molecular and network-level explanations. In *KDD 2025*, 2025.

[178]Linqian Zhao and others. A mutual-guided co-attention mechanism and heterogeneous attribute graph-based framework for drug-drug interaction event prediction. *Chemometrics and Intelligent Laboratory Systems*, 2025.

[179]Qiujie Lv and others. 3d graph neural network with few-shot learning for predicting drug–drug interactions in scaffold-based cold start scenario. *Neural Networks*, 2023.

[180]Idris Demirsoy and Adnan KARAİBRAHİMOĞLU. Identifying drug interactions using machine learning. *Advances in Clinical and Experimental Medicine*, 2023.

[181]Yan Xia, An Xiong, Zilong Zhang, Quan Zou, and Feifei Cui. A comprehensive review of deep learning-based approaches for drug–drug interaction prediction. *Briefings in Functional Genomics*, 24:elae052, 2025.

[182]Ba-Hoang Tran and others. A multifaceted approach to drug–drug interaction extraction with fusion strategies. *Journal of Biomedical Informatics*, 2025.

[183]Shiwei Gao, Jingjing Xie, and Yizhao Zhao. A multi-source drug combination and omnidirectional feature fusion approach for predicting drug-drug interaction events. *Journal of Biomedical Informatics*, 162:104772, 2025.

[184]Aga Basit Iqbal and others. A review of deep learning algorithms for modeling drug interactions. *Multimedia Systems*, 2024.

[185]Deepa Kumari and others. A study on improving drug–drug interactions prediction using convolutional neural networks. *Applied Soft Computing*, 2024.

[186]Liangcheng Dong and others. A substructure-aware graph neural network incorporating relation features for drug–drug interaction prediction. *Quantitative Biology*, 2024.

[187]Mir Mansoor Ahmad, Zuraini Binti Ali Shah, and Hui Wen Nies. A systematic review of molecular structures, knowledge graphs, and cold-start scenario in drug-drug interaction prediction. *Computers in Biology and Medicine*, 190:110122, 2025.

[188]Linqian Zhao and others. Adaptive multi-kernel graph neural network for drug-drug interaction prediction. *Interdisciplinary Sciences: Computational Life Sciences*, 17:409–423, 2025.

[189]Sabir Ali and others. An accurate prediction of drug–drug interactions and side effects by using integrated convolutional and bilstm networks. *Chemometrics and Intelligent Laboratory Systems*, 2025.

[190]S. K. Mydhili and others. An optimised mobilenet v2 attention parallel network for predicting drug–drug interactions through combining local and global features. *Biopharmaceutics Drug Disposition*, 2025.

[191]Peng Gao and others. Medical knowledge graph question answering for drug–drug interaction prediction based on multi-hop machine reading comprehension. *CAAI Transactions on Intelligence Technology*, 2024.

[192]Qijin Yin and others. Deepdrug: A general graph-based deep learning framework for drug-drug interactions and drug-target interactions prediction. *Quantitative Biology*, 2023.

[193]Fadwa Alrowais and others. Clinical decision support systems to predict drug–drug interaction using multilabel long short-term memory with an autoencoder. *International Journal of Environmental Research and Public Health*, 2023.
[194]Simone Scagnoli and others. Clinical impact of drug-drug interactions on abemaciclib in the real-world experience of ab-italy study. *npj Breast Cancer*, 2024.
[195]Jie Yang and others. Ddi-mug: Multi-aspect graphs for drug-drug interaction extraction. *Frontiers in Digital Health*, 2023.
[196]Pooja Gupta and others. Decondffuse: Predicting drug–drug interaction using joint deep convolutional transform learning and decision forest fusion framework. *Expert Systems with Applications*, 2023.
[197]Zhaohong Sun and others. Deep dynamic patient similarity analysis: Model development and validation in icu. *Computer Methods and Programs in Biomedicine*, 2022.
[198]Xinyue Li and others. Deep learning for drug-drug interaction prediction: A comprehensive review. *Quantitative Biology*, 2024.
[199]Thao Pham and others. Deeparv: ensemble deep learning to predict drug-drug interaction of clinical relevance with antiretroviral therapy. *npj Systems Biology and Applications*, 2024.
[200]Xinkun Hao and others. Enhancing drug–drug interaction prediction by three-way decision and knowledge graph embedding. *Granular Computing*, 8:67–76, 2023.
[201]Muhammed Ali. Pala. Deepinsulin-net: A deep learning model for identifying drug interactions leading to specific insulin-related adverse events. *Sakarya University Journal of Computer and Information Sciences*, 2025.
[202]Eugene Jeong and others. Discovering clinical drug-drug interactions with known pharmacokinetics mechanisms using spontaneous reporting systems and electronic health records. *Journal of Biomedical Informatics*, 2024.
[203]Maryam KafiKang and Abdeltawab Hendawi. Drug-drug interaction extraction from biomedical text using relation biobert with blstm. *Machine Learning and Knowledge Extraction (MDPI)*, 2023.
[204]José Machado and others. Drug–drug interaction extraction-based system: A natural language processing approach. *Expert Systems*, 2025.
[205]Mingliang Dou and others. Drug–drug interaction relation extraction based on deep learning: A review. *ACM Computing Surveys*, 2024.
[206]Ming Chen and others. Drug-target interactions prediction based on signed heterogeneous graph neural networks. *Chinese Journal of Electronics*, 2024.
[207]Junlong Ma and others. Efficient analysis of drug interactions in liver injury: a retrospective study leveraging natural language processing and machine learning. *BMC Medical Research Methodology*, 2024.
[208]Bihter Das and others. Gainet: Enhancing drug–drug interaction predictions through graph neural networks and attention mechanisms. *Chemometrics and Intelligent Laboratory Systems*, 2025.
[209]Jinchen Sun and Haoran Zheng. Hdn-ddi: a novel framework for predicting drug-drug interactions using hierarchical molecular graphs and enhanced dual-view representation learning. *BMC Bioinformatics*, 2025.
[210]Yue Luo, Lei Deng, and Zhijian Huang. Hln-ddi: hierarchical molecular representation learning with co-attention mechanism for drug–drug interaction prediction. *BMC Bioinformatics*, 26(152), 2025.
[211]Yu Li and others. Attention-based learning for predicting drug-drug interactions in knowledge graph embedding based on multisource fusion information. *International Journal of Intelligent Systems*, 2024.
[212]Lizhen Jiang and Sensen Zhang. Enhancing knowledge graph embedding with hierarchical self-attention and graph neural network techniques for drug-drug interaction prediction in virtual reality environments. *Symmetry*, 2024.

[213]Lianwei Zhang and others. Fsrm-ddie: few-shot learning methods based on relation metrics for the prediction of drug-drug interaction events. *Applied Intelligence*, 2024.
[214]Qamar M. Aljanadi Alsfouk, Bshra A. and Mona M. Almutairi. Evaluation of drug interactions in outpatients taking antipsychotic medications. *Frontiers in Pharmacology*, 2025.
[215]Adi Jabarin and others. Eravacycline, an antibacterial drug, repurposed for pancreatic cancer therapy: insights from a molecular-based deep learning model. *Briefings in Bioinformatics*, 2024.
[216]Tianyi Zang Zhang, Chengcheng and Tianyi Zhao. Kge-unit: toward the unification of molecular interactions prediction based on knowledge graph and multi-task learning on drug discovery. *Briefings in Bioinformatics*, 2024.
[217]Li Meng and others. Learning personalized drug features and differentiated drug-pair interaction information for drug–drug interaction prediction. *Neural Networks*, 2025.
[218]Peter Petschner and others. Machine learning for predicting drug–drug interactions: Graph neural networks and beyond. *Current Opinion in Systems Biology*, 2025.
[219]Sven Van Laere and others. Machine learning techniques outperform conventional statistical methods in the prediction of high risk qtc prolongation related to a drug-drug interaction. *Journal of Medical Systems*, 2022.
[220]Ha Young Jang and others. Machine learning-based quantitative prediction of drug exposure in drug-drug interactions using drug label information. *npj Digital Medicine*, 2022.
[221]Abedin Keshavarz and Amir Lakizadeh. Pu-mlp: A pu-learning based method for polypharmacy side-effects detection based on multi-layer perceptron and feature extraction techniques. *Intelligence-Based Medicine*, 2025.
[222]Eman Saad, Sherif Kishk, Amr Ali-Eldin, and Ahmed I. Saleh. Sb-agt: A stochastic beam search-enhanced attention-based gumbel tree framework for drug-drug interaction extraction from biomedical literature. *Computers in Biology and Medicine*, 2025.
[223]Rongpei Li, Yufang Zhang, Heqi Sun, Shenggeng Lin, Guihua Jia, Yitian Fang, Chen Zhang, et al. Towards interpretable drug interaction prediction via dual-stage attention and bayesian calibration with active learning. *PeerJ Computer Science*, 2025.
[224]Jiang Xie, Chang Zhao, Jiaming Ouyang, Hongjian He, Dingkai Huang, Mengjiao Liu, Jiao Wang, and Wenjun Zhang. Tp-ddi: A two-pathway deep neural network for drug–drug interaction prediction. *Interdisciplinary Sciences: Computational Life Sciences*, 2022.
